# Recommendations for the management of MPS VI: systematic evidence- and consensus-based guidance

**DOI:** 10.1186/s13023-019-1080-y

**Published:** 2019-05-29

**Authors:** Mehmet Umut Akyol, Tord D. Alden, Hernan Amartino, Jane Ashworth, Kumar Belani, Kenneth I. Berger, Andrea Borgo, Elizabeth Braunlin, Yoshikatsu Eto, Jeffrey I. Gold, Andrea Jester, Simon A. Jones, Cengiz Karsli, William Mackenzie, Diane Ruschel Marinho, Andrew McFadyen, Jim McGill, John J. Mitchell, Joseph Muenzer, Torayuki Okuyama, Paul J. Orchard, Bob Stevens, Sophie Thomas, Robert Walker, Robert Wynn, Roberto Giugliani, Paul Harmatz, Christian Hendriksz, Maurizio Scarpa, MPS Consensus Programme Steering Committee, Mehmet Umut Akyol, Tord D. Alden, Hernan Amartino, Jane Ashworth, Kumar Belani, Kenneth I. Berger, Andrea Borgo, Elizabeth Braunlin, Yoshikatsu Eto, Jeffrey I. Gold, Andrea Jester, Simon A. Jones, Cengiz Karsli, William Mackenzie, Diane Ruschel Marinho, Andrew McFadyen, Jim McGill, John J. Mitchell, Joseph Muenzer, Torayuki Okuyama, Paul J. Orchard, Bob Stevens, Sophie Thomas, Robert Walker, Robert Wynn, MPS Consensus Programme Co-Chairs, Roberto Giugliani, Paul Harmatz, Christian Hendriksz, Maurizio Scarpa

**Affiliations:** 1 Department of Otolaryngology, Hacettepe University, Ankara, Turkey; 2 Department of Neurosurgery, Ann & Robert H. Lurie Children’s Hospital of Chicago, Northwestern University Feinberg School of Medicine, Chicago, IL USA; 3 Child Neurology Department, Hospital Universitario Austral, Buenos Aires, Argentina; 4 Department of Paediatric Ophthalmology, Manchester Royal Eye Hospital, Manchester University NHS Foundation Trust, Manchester, UK; 5 Department of Anesthesiology, University of Minnesota, Minneapolis, MN USA; 6 Departments of Medicine and Neuroscience and Physiology, New York University School of Medicine, André Cournand Pulmonary Physiology Laboratory, Bellevue Hospital, New York, NY USA; 7 Orthopaedics Clinic, Padova University Hospital, Padova, Italy; 8 Division of Pediatric Cardiology, University of Minnesota, Minneapolis, MN USA; 9 Advanced Clinical Research Centre, Institute of Neurological Disorders, Kanagawa, Japan and Department of Paediatrics/Gene Therapy, Tokyo Jikei University School of Medicine, Tokyo, Japan; 10 Keck School of Medicine, Departments of Anesthesiology, Pediatrics, and Psychiatry & Behavioural Sciences, Children’s Hospital Los Angeles, Department of Anesthesiology Critical Care Medicine, 4650 Sunset Boulevard, Los Angeles, CA USA; 11 Hand and Upper Limb Service, Department of Plastic Surgery, Birmingham Women’s and Children’s Hospital, Birmingham, UK; 12 Willink Biochemical Genetic Unit, Manchester Centre for Genomic Medicine, St Mary’s Hospital, Manchester University NHS Foundation Trust, Manchester, UK; 13 Department of Anesthesiology and Pain Medicine, The Hospital for Sick Children, Toronto, Canada; 14 Department of Orthopedics, Nemours/Alfred I. Dupont Hospital for Children, Wilmington, DE USA; 15 Department of Ophthalmology, UFRGS, and Ophthalmology Service, HCPA, Porto Alegre, Brazil; 16 The Isaac Foundation, Campbellford, ON Canada; 17 Department of Metabolic Medicine, Queensland Children’s Hospital, Brisbane, Australia; 18 Division of Pediatric Endocrinology, Montreal Children’s Hospital, Montreal, QC Canada; 19 Department of Pediatrics, School of Medicine, University of North Carolina at Chapel Hill, Chapel Hill, NC USA; 20 Department of Clinical Laboratory Medicine, National Centre for Child Health and Development, Tokyo, Japan; 21 Division of Blood and Marrow Transplantation, Department of Pediatrics, University of Minnesota, Minneapolis, MN USA; 22 MPS Society, Amersham, Buckinghamshire UK; 23 Department of Paediatric Anaesthesia, Royal Manchester Children’s Hospital, Manchester, UK; 24 Department of Paediatric Haematology, Royal Manchester Children’s Hospital, Manchester, UK; 25 Department of Genetics, UFRGS, and Medical Genetics Service, HCPA, Porto Alegre, Brazil; 26 UCSF Benioff Children’s Hospital Oakland, Oakland, CA USA; 27 Steve Biko Academic Hospital, University of Pretoria, Pretoria, South Africa; 28 Center for Rare Diseases at Host Schmidt Kliniken, Wiesbaden, Germany and Department of Paediatrics, University of Padova, Padova, Italy

**Keywords:** Maroteaux-Lamy syndrome, Mucopolysaccharidosis, MPS VI, Management guidelines, Galsulfase, Enzyme replacement therapy, ERT, Haematopoietic stem cell transplantation, HSCT, Surgery, Anaesthetics

## Abstract

**Introduction:**

Mucopolysaccharidosis (MPS) VI or Maroteaux-Lamy syndrome (253200) is an autosomal recessive lysosomal storage disorder caused by deficiency in *N*-acetylgalactosamine-4-sulfatase (arylsulfatase B). The heterogeneity and progressive nature of MPS VI necessitates a multidisciplinary team approach and there is a need for robust guidance to achieve optimal management. This programme was convened to develop evidence-based, expert-agreed recommendations for the general principles of management, routine monitoring requirements and the use of medical and surgical interventions in patients with MPS VI.

**Methods:**

26 international healthcare professionals from various disciplines, all with expertise in managing MPS VI, and three patient advocates formed the Steering Committee group (SC) and contributed to the development of this guidance. Members from six Patient Advocacy Groups (PAGs) acted as advisors and attended interviews to ensure representation of the patient perspective. A modified-Delphi methodology was used to demonstrate consensus among a wider group of healthcare professionals with expertise and experience managing patients with MPS VI and the manuscript has been evaluated against the validated Appraisal of Guidelines for Research and Evaluation (AGREE II) instrument by three independent reviewers.

**Results:**

A total of 93 guidance statements were developed covering five domains: (1) general management principles; (2) recommended routine monitoring and assessments; (3) enzyme replacement therapy (ERT) and hematopoietic stem cell transplantation (HSCT); (4) interventions to support respiratory and sleep disorders; (5) anaesthetics and surgical interventions. Consensus was reached on all statements after two rounds of voting. The greatest challenges faced by patients as relayed by consultation with PAGs were deficits in endurance, dexterity, hearing, vision and respiratory function. The overall guideline AGREE II assessment score obtained for the development of the guidance was 5.3/7 (where 1 represents the lowest quality and 7 represents the highest quality of guidance).

**Conclusion:**

This manuscript provides evidence- and consensus-based recommendations for the management of patients with MPS VI and is for use by healthcare professionals that manage the holistic care of patients with the intention to improve clinical- and patient-reported outcomes and enhance patient quality of life. It is recognised that the guidance provided represents a point in time and further research is required to address current knowledge and evidence gaps.

**Electronic supplementary material:**

The online version of this article (10.1186/s13023-019-1080-y) contains supplementary material, which is available to authorized users.

## Background

Mucopolysaccharidoses (MPS) are part of a clinically heterogeneous group of diseases known as lysosomal storage disorders (LSDs), of which there are over 60 different types. Symptoms of MPS occur because of deficiencies in enzymes that break down glycosaminoglycans (GAGs) [1–3]. Eleven different enzymes are responsible for the stepwise degradation of GAGs, deficiencies in each of which are responsible for seven different types of MPS [4]. Patients with MPS typically seem healthy at birth, but symptoms usually appear during early childhood as the concentration of GAGs in cells increases. The pursuant effect on tissues and organs cause severe morbidity and reduced life expectancy [5, 6]. Clinical features can vary according to MPS subtype, but coarse features, organomegaly, skeletal and joint abnormalities, dysfunction in vision and hearing and cardiorespiratory problems are common across all MPS subtypes [1].

MPS VI or Maroteaux-Lamy syndrome (253200) is an autosomal recessive MPS disorder caused by deficiency in *N*-acetylgalactosamine-4-sulfatase (arylsulfatase B, ASB; EC 3.1.6.12). Birth prevalence has been reported to range from 1 in 43,261 live births in Turkish immigrants living in Germany [7] to 1 in 1,505,160 live births in Sweden [8, 9]. ASB catalyses the breakdown of dermatan sulphate, which is present particularly in the skin, but is also found in tendons, blood vessels, airways and heart valves [10]. Preclinical data have shown that dermatan sulphate effects an inflammatory response via the tumour necrosis factor (TNF) pathway, and its accumulation results in apoptosis of chondrocytes and ensuing progressive arthropathy [11, 12].

MPS VI is classified according to severity of symptoms and is typically termed as being either slowly or rapidly progressing; however, it is now known that an intermediate form between slowly and rapidly progressing MPS also exists. Presentation differs according to age of onset and velocity of disease progression; and higher urinary GAG levels are associated with rapidly progressing disease [13]. However, a large number of mutations of the ASB gene have been identified and are believed to be responsible for the heterogeneity in presentation [9]. Although elevated urinary GAGs and increased dermatan sulphate concentrations are markers of the disease, these alone do not provide a definitive diagnosis. Diagnosis is generally accepted by confirmation in an accredited laboratory of ASB enzyme activity in cultured fibroblasts or isolated leukocytes of < 10% of the lower limit of normal and/or demonstration of two disease-causing mutations [9, 14, 15]. Symptoms of MPS VI include decreased growth velocity, coarse facial features, skeletal deformities, frequent upper-airway infections, enlarged liver and spleen, hearing loss, joint stiffness and coarse hair [14]. Abnormalities of cardiac valve anatomy and function are present in all patients with MPS VI [16] and are attributed to the deposition of dermatan sulphate within the cardiac valves.

MPS are rare diseases; therefore, the small patient population of patients with MPS VI precludes the generation of large datasets through participation in Phase 3 trials, and consequently the availability of top-level evidence through meta-analyses. Although guidance for MPS VI has been published [14], owing to the lack of available evidence, the provision of credible guidelines in rare diseases requires the use of robust methodology to provide expert-driven, consensus-based guidance. The guidance provided in this manuscript represents a transition from expert opinion in prior documents to a validated approach that includes a comprehensive literature review and a modified Delphi process.

## Objectives

The scope of the programme was to develop guidance for the management of two MPS without neurocognitive manifestations, namely MPS IVA and MPS VI. This manuscript provides robust evidence- and consensus-based guidance for the management of adult and paediatric patients with MPS VI. The guidance is comprised of a holistic set of recommendations for the timely and appropriate use of medical and surgical interventions and management of the natural history of MPS, with the intention to maintain and enhance patient quality of life and improve clinical- and patient reported- outcomes. The guidance is intended for use by healthcare professionals who manage the care of patients with MPS VI, in particular paediatricians and geneticists, and aims to enhance multidisciplinary practice across specialisms. It also provides specific guidance for other specialists (Table 1) and stakeholders in the health services who are in contact with patients with MPS and is a useful reference for patient advocates, patients and their families. Table 1 describes the areas of clinical focus covered within this guidance and the corresponding recommended speciality focus.

**Table 1:** Clinical areas of focus and recommended speciality focus

Table	Recommended speciality focus
Table 2: General principles for the management of patients with MPS VI	all
Table 3: Recommended routine monitoring and assessments in patients with MPS VI	all
Table 4: Guidance statement for galsulfase	geneticist, metabolic physician, paediatrician, nurse, physiotherapist
Table 5: Guidance statements for HSCT	anaesthetist, bone marrow transplant expert/hematopoietic stem cell transplant expert, geneticist, paediatrician, nurse
Table 6: Guidance statements for continuous positive airway pressure (CPAP), non-invasive positive pressure ventilation (NIPPV), oxygen supplementation and hypercapnia monitoring	anaesthetist, ear-nose-throat specialist, geneticist, paediatrician, respiratory physician/pulmonologist, nurse
Table 7: Guidance statements for anaesthesia	all
Table 8: Guidance statements for hip reconstruction, hip replacement and growth modulation surgeries	anaesthetist, geneticist, orthopaedic surgeon, neurosurgeon, paediatrician, physiotherapist
Table 9: Guidance statements for decompression of the spinal cord, spinal stabilisation and thoracolumbar kyphoscoliosis	anaesthetist, geneticist, orthopaedic surgeon, neurosurgeon, paediatrician, physiotherapist
Table 10: Guidance statement for corneal transplantation	anaesthetist, geneticist, ophthalmologist, paediatrician
Table 11. Guidance statements for decompression of the median nerve, tenosynovectomy and pulley release	anaesthetist, geneticist, hand surgeon, orthopaedic surgeon, neurosurgeon, paediatrician
Table 12: Guidance statement for cardiac valve replacement and left ventricular apical aneurysms	anaesthetist, cardiologist, geneticist, paediatrician
Table 13: Guidance statements for tonsillectomy and/or adenoidectomy, tracheostomy and insertion of ventilation tubes	anaesthetist, geneticist, ear-nose-throat specialist, paediatrician, respiratory physician/pulmonologist

This guidance was developed as part of a broader consensus programme that also covered the management of MPS IVA, the results of which are published in a companion article (*Recommendations for the management of MPS IVA: systematic evidence- and consensus-based guidance*).

## Methods and process

As the methodology for guidance pertaining to both MPS IVA and MPS VI consensus was conducted in parallel, the full methodology is reported in a companion article: *Recommendations for the management of MPS IVA: systematic evidence- and consensus-based guidance*.

Briefly, the methodology included: a systematic expert mapping process to identify the programme Co-Chairs; recommendations from the Co-Chairs to align the international Steering Committee (SC) group; numerous face-face and online SC meetings to define the clinical questions to be answered by the guidance according to the P.I.C.O methodology (Additional file 1: Appendix 1a); a systematic literature review to identify the evidence base for each clinical question in accordance with the Preferred Reporting Items for Systematic Reviews and Meta-Analyses (PRISMA) statement (Additional file 1 Appendices 1b and 1c) [17]; assessment of the quality of evidence level for each paper using the Oxford Centre for Evidence-based Medicine criteria (Additional file 1: Appendix 2 and Additional files 2 and 3); consultations with six global Patient Advocate Groups (PAGs) [listed in the acknowledgements section of the manuscript] to generate insights to inform the development of guidance statements; drafting of guidance statements by the SC via a series of face-to-face, online meetings and email correspondence; validation of the guidance statements by a modified-Delphi survey [18, 19] (the full results including the number of voters/statement, respondent specialisms and geographies, and respondent feedback to guidance statements are included within Additional files 4 and 5); grading of recommendation statements based on the average evidence level for each supporting reference (Additional file 1: Appendix 2 and Additional files 2 and 3) and independent assessment of the manuscript by three reviewers using the Appraisal of Guidelines for Research and Evaluation (AGREE) II Instrument [20] (full information including the scores from the two rounds of AGREE II evaluation can be found in Additional file 1: Appendix 3).

The SC consisted of four Co-Chairs and a further 22 healthcare professionals were convened from a wide geographic spread covering multiple medical specialties including: anaesthesia, ear, nose and throat (ENT) surgery, cardiology, endocrinology, genetics, hand surgery, haematopoietic stem cell transplantation (HSCT), neurosurgery, ophthalmology, orthopaedic surgery, paediatrics, pain management and pulmonology. To ensure the patient view was considered, three representatives from PAGs also formed part of the SC group. The SC defined the scope of the programme, identified the medical and surgical interventions to be covered in the guidance and provided search terms for the literature review. More information about the SC, including their details, competing interests and contributions can be found in the declarations section of the manuscript.

### Setting the clinical questions to be answered by the guidance

The SC group developed the clinical questions to be answered by the guidance according to the patient, interventions, comparator and outcome (P.I.C.O.) methodology (outlined in Additional file 1**:** Appendix 1a) and are shown below.

1. What are the general principles for the management of adult and paediatric MPS VI?
2. What are the recommended routine monitoring and assessments that should be used to track the natural history of adult and paediatric MPS VI and indicated interventions to be used in the management of the common symptoms of MPS VI?
3. For patients with adult and paediatric MPS VI, what is the impact on clinical outcomes and safety/tolerability of:
  Interventions that address the underlying enzyme deficiency
    ◦ ERT
    ◦ HSCT
  Interventions used to manage the symptoms of MPS
    ◦ Respiratory and sleep disorders
    ◦ Anaesthetics
    ◦ Limb and spinal surgeries
    ◦ Ophthalmic surgeries
    ◦ Cardio-thoracic surgeries
    ◦ ENT surgeries

The programme also had a secondary focus to highlight current evidence gaps and provide recommendations for future treatment directions. This programme did not cover the following topics: diagnosis, validation of new clinical outcome assessment tools (e.g. to assess patient-reported outcomes) and defining minimal clinically important differences for MPS IVA/VI.

### Measures to address independence

The programme was funded by BioMarin; however, they remained uninvolved throughout the whole process and did not influence the scope or content of the programme. The funder was absent from all SC meetings, remained blinded to the guidance statements and was not involved in the publication process. An independent secretariat (Lucid Partners Ltd) managed the programme and provided editorial support. The SC led the scope and content of the programme, including the development of guidance statements. Conflicts of interests for all SC members (found in the declarations section) were recorded at the start of the programme and updated throughout the programme. Following the systematic expert mapping exercise, it was noted that some of the SC had previously worked on a consultancy basis with the programme sponsor, who hold the marketing authorisation for approved pharmaceutical therapy in MPS VI. Efforts were therefore taken to ensure representation from leading experts across other treatment modalities, including HSCT/BMT on the Steering Committee panel and during the modified-Delphi voting process, where a large number of physicians across multiple specialisms and geographies were engaged. At several stages during the process, the SC were also required to provide updated conflict of interest disclosures.

## Results

The results of the modified-Delphi voting process are described in more detail in the companion publication (*Recommendations for the management of MPS IVA: systematic evidence- and consensus-based guidance*.

After two rounds of anonymised voting via an online survey (from a pool of 197 MPS physicians across 35 clinical areas of focus in 25 countries worldwide), consensus was reached on 94 validated guidance statements pertaining to the management of patients with MPS VI (further information about the modified-Delphi process, including: the number of voters/statement, respondent specialisms and geographies, and respondent feedback to guidance statements are shown in Additional files 4 and 5).

Three independent reviewers (listed in the acknowledgements section of the manuscript) assessed the guidance for methodological rigour and transparency against the validated Appraisal of guidelines for research and evaluation (AGREE II) instrument. The guidance documents were given an overall guideline assessment score of 5.3/7 (where 1 represents the lowest quality, and 7 represents the highest quality). Full information including the assessment scores across each domain criteria are outlined in Additional file 1: Appendix 3.

### Guidance statements

#### General principles (Table 2)

The diagnosis of MPS VI was deemed out of scope for this guidance, but details can be found elsewhere [14, 15]. The SC noted that if newborn screening is made available, this would facilitate earlier diagnosis and intervention for patients with MPS VI, which is likely to change the course of their disease. As the most serious surgical complications occur in patients with advanced MPS, surgical procedures (particularly airway procedures) where indicated, should be conducted as soon as possible. Guidelines for pain management in patients with MPS have recently been published [21, 22].

**Table 2:** General principles for the management of patients with MPS VI

Statement	Percentage consensus
All guidance statements are evidence Grade D (level 5 expert clinical opinion)	
Diagnosis of MPS VI during infancy is critical to optimise patient outcomes	98%
The first consultation should be conducted by a physician with experience of treating MPS as soon as possible after diagnosis. This should include a full discussion of disease pathology, progression, treatment options and management. Ongoing information should be provided to optimise patient outcomes	97%
Patients and caregivers should receive ongoing psychosocial support from a social worker and/or psychologist, and should be directed towards a MPS society or relevant patient organisation in their country	94%
A comprehensive medical history and multi-system evaluation should be conducted within days of diagnosis to set a baseline for ongoing assessments and evaluate the physical and neurological manifestations of disease, functional ability and disease burden	88%
Ongoing and regular multi-system monitoring, and assessments are recommended to track the natural history of MPS VI, monitor the impact of treatment and assess the need for treatment interventions to manage the symptoms of MPS VI. These should be conducted at every clinic visit, annually or in some cases as clinically indicated (for example pre- and post-operatively)	100%
Timely interventions are recommended where clinically indicated by monitoring, to help avoid irreversible damage caused by the natural history of MPS VI, and to manage the disease manifestations and maintain long-term quality of life (QoL)	99%
A multidisciplinary team (MDT) of metabolic specialists, surgeons and allied healthcare professionals (including, but not limited to: nurses, physiotherapists, occupational therapists, psychologists and audiologists) is required to manage the diverse range of disease manifestations of MPS VI	99%
Co-ordination of the entire MDT care team is required prior to any procedure to determine the need for surgery, to discuss the benefits and risks of combining surgeries to minimise the need for multiple anaesthetics and to decide the optimal order of procedures. The decision to combine surgeries should take into consideration the surgical and intubation time, and complexity of procedures	93%
The risks and benefits of any intervention and the competing risks of other medical problems should be assessed and discussed with patients, families and caregivers such that they can make an informed decision on the appropriateness of the therapy/surgery	100%
Surgical procedures should be performed by (or under the guidance of) specialist surgeons and anaesthetists with experience of MPS, in medical centres with intensive care units	99%
Management of pain should be a fundamental part of the care of patients with MPS VI, with the aim of improving QoL and maintaining mobility. Refer to general guidelines for pain management	100%

#### Recommended routine monitoring and assessments (Table 3)

**Table 3:** Recommended routine monitoring and assessments in patients with MPS VI

Statement	Percentage consensus
All guidance statements are evidence Grade D (based on level 5 expert clinical opinion), unless otherwise stated	
Physical examination	
A physical examination should be performed during every visit to assess general health, growth, vital signs, abdominal organ size, presence of hernia, neurologic function (including gait), joint stiffness, and functions of the eyes, ears, heart and lungs	90%
Routine physical examination can also identify signs of potential respiratory problems, such as an enlarged tongue or sniffing position	90%
Radiology	
While X-rays are essential to identify the natural history of disease and response to treatment, efforts should be made to minimise radiation exposure, and images should be requested only when clinically useful	85%
Hips: an anteroposterior (AP) pelvis radiograph should be performed at diagnosis and as clinically indicated (based on physical examination or reports of pain) to quantify hip dysplasia or identify early signs of hip migration [23]	88%
Lower limbs: in patients with clinical evidence of valgus deformity of the lower limbs, standing AP radiographs of lower extremities should be performed prior to guided growth surgery [24]	100%
Spine: standing or sitting plain radiography of the cervical and thoracolumbar spine to examine for spinal deformities is recommended in patients with MPS VI at diagnosis and every 2–3 years thereafter, or sooner if clinically indicated [24] Evidence Grade: C (level 3/4 studies)	85%
Magnetic resonance imaging (MRI) of the whole spine (in neutral position) should be performed annually in children with MPS VI to assess for spinal cord injury. The frequency may be reduced for adult patients with stable imaging who do not display symptoms^a^	84%
Flexion/extension MRI of cervical spine may be needed to identify changes in spinal canal and spinal cord	86%
MRI of the brain is recommended at diagnosis in patients with MPS VI, and should be repeated as needed in individuals with clinical suspicion of hydrocephalus	80%
MRI of the brain and spinal cord in patients with MPS VI may require sedation or general anaesthesia depending on patient age and cooperation. General anaesthesia carries substantial risk for patients with MPS	95%
Flexion/extension computerised tomography (CT) of the craniocervical junction may be considered in individuals with MPS VI if MRI is not available or if sedation is not possible	92%
The presence of specific radiological signs may indicate the need for surgical intervention to correct skeletal deformities; however, there is insufficient evidence to support preventative surgery based on radiological findings	88%
Endurance	
Choice of assessment depends on the patient’s physical and developmental ability [25]	97%
Baseline assessment is the most important and ideally two values should be obtained as a minimum. Consistent protocols should be used when performing repeat measurements to minimise variability	95%
Annual endurance testing using 6-min walk test (6MWT) is recommended, as per the American Thoracic Society guidelines [13, 25, 26]	87%
In patients with limited ambulation who are unable to perform the 6MWT, endurance should be assessed via alternative methods such as an adapted timed 25-ft walk test (T25FW)	76%
Endurance testing is also recommended prior to initiation of ERT and annually thereafter as a measure of treatment efficacy and to provide early evidence of possible neurologic or skeletal issues	87%
Growth	
Assessment of growth should be performed at each clinic visit as part (ideally every 6 months) of a regular physical examination and should include: standing height (sitting height if the patient is unable to stand), length (supine position), weight, head circumference (≤3 years), Tanner pubertal stage (until maturity)	95%
Height and weight should also be measured before initiation of ERT and at every clinic visit thereafter (ideally every 6 months) to evaluate the impact of treatment	95%
Urinary glycosaminoglycan (uGAG) levels	
Urinary GAG levels should be tested prior to starting galsulfase and every 6 months thereafter to determine the pharmacodynamic effects of ERT [13] Evidence Grade: C (level 3/4 studies)	97%
Measurement of total uGAG levels may be performed using standard dye-based quantitative methods, preferably in the same laboratory and assessed against age-related reference values	93%
Where available tandem mass spectrometry may be used to assess levels of specific GAGs (such as dermatan sulphate) ^b^ [27–32] Evidence Grade: C (level 3/4 studies)	97%
Cardiac function	
Initial cardiac evaluation should be performed at the time of diagnosis and include assessment of vital signs with measurement of oxygen saturation, right arm and leg blood pressure measurements, careful auscultation, full transthoracic two-dimensional and Doppler echocardiogram, and 12-lead electrocardiogram (ECG) [33]	100%
Longer ECG monitoring (prolonged Holter/event monitoring) may be considered in older patients, especially if they have symptoms of black outs, unexpected falls or dizziness	96%
Follow-up in expert centres should be annually initially, but may be extended to every 2–3 years if there is no evidence of cardiac abnormality [34, 35]	92%
Additional cardiac assessment, including a standard ECG, should be performed prior to any surgical procedure requiring general anaesthesia^c^ [34, 35]	92%
Neurological exam	
A detailed neurological examination should be performed at every clinic visit (minimally every 6 months) and, where possible these should correlate with imaging studies of the spine to detect early spinal stenosis or instability compromising the cervical cord. For patients without clinical or radiographic concern, annual neurological examination may be sufficient [36]	87%
Standard MRI of the cervical spine should be performed to assess for presence of spinal cord compression. In the absence of significant spinal cord compression, proceed with flexion/extension MRI to confirm the presence of worsening spinal cord compression with motion^d^	78%
Upper limb function	
Symptoms of carpal tunnel syndrome (CTS) are often atypical in patients with MPS VI, therefore recommend clinical examination, assessment of range of finger movement and strength, electrophysiology nerve conduction assessment and detailed medical history to be performed at diagnosis and annually thereafter	89%
Standardized clinical examination, assessment of active and passive range of movement and nerve conduction studies (NCS) are recommended to assess hand and upper limb function in individuals with MPS VI	89%
Respiratory function and sleep disorder	
Evaluation of respiratory function by spirometry, including forced vital capacity (FVC) and maximum voluntary ventilation (MVV), should be performed to assess changes in lung volume and obstruction in children over 5 years of age	97%
Respiratory function should be assessed annually until children stop growing, and every 2–3 years thereafter, provided that respiratory symptoms remain unchanged. Additional testing should be performed if respiratory symptoms change or if intercurrent illnesses occur	91%
Normative values are not available, therefore change in absolute volume from patient’s own baseline will be the best indicator of deterioration or improvement	97%
Measurement of respiratory rate and arterial oxygen saturation before and after annual endurance testing is recommended	86%
Evaluation of gas exchange and respiratory function is also recommended before any planned air travel, to ensure safety during the flight	86%
To identify symptoms of sleep apnoea, patients should be asked to report presence of snoring and morning headaches at every clinic visit^e^	100%
Overnight sleep study (polysomnography) is recommended at diagnosis (if possible, and no later than 2 years of age), and every 3 years thereafter or when signs and symptoms of obstructive sleep apnoea (OSA) are noted [37, 38]	94%
Ear-nose-throat (ENT)	
ENT examination, including tympanometry^f^, should be conducted every 3–6 months during childhood and every 6–12 months thereafter	91%
ENT examination in patients with MPS VI should include visualization of the upper respiratory tract to determine diagnosis, management and assist in pre-operative planning. Endoscopic examinations should be recorded and kept, to monitor disease progression	92%
Fibreoptic examination in patients with MPS VI should be performed at diagnosis and at least annually thereafter, or as clinically indicated. For those individuals who require general anaesthesia, ENT examination should be performed during the pre-operative evaluation for other surgical procedures	83%
Upper airway CT, focused on airway anatomy preferably with reconstruction, may be useful to identify the area of the abnormality and possible cause of obstruction in patients with MPS VI with suspected obstruction or malacia^g^	92%
Age-adjusted audiometric assessment as a baseline objective hearing evaluation should be conducted in the first clinic visit and repeated annually to assess conductive and sensory-neural hearing loss [39]	100%
If speech problems are determined during ENT examination, an assessment by a speech pathologist should be conducted	100%
Balance tests should be conducted if the patient has a history of balance problems	95%
Ophthalmological function	
Age-appropriate evaluations by an ophthalmologist is recommended every 6 months if possible, or at least annually	90%
Ophthalmic assessment may include visual acuity, refraction, slit-lamp examination of cornea, funduscopic evaluation including optic nerve, and measurement of intraocular pressure	100%
Intraocular pressure monitoring and pachymetry may be considered prior to corneal transplant	100%
Evaluation of oral health by dentist	
Recommend close monitoring of dental development (at least annually) to prevent caries and attrition as is monitoring of occlusion and chewing functions	100%
The need for subacute bacterial endocarditis (SBE) prophylaxis prior to dental procedures should be assessed by a cardiologist	100%
Disease burden	
Annual assessment of patient-reported outcomes is recommended for: pain severity, QoL (as assessed by reproducible and age-appropriate questionnaires [e.g. EQ-5D-5 L]), fatigue, and activities of daily living (ADL; as assessed by functional tests [6MWT/T25FW]) and age-appropriate ADL questionnaires (e.g. MPS Health Assessment Questionnaire [MPS HAQ]), and assessment of wheelchair/walking aid use	97%
These assessments may have to be adapted both for language, culture, and individual physical limitations as they have not been validated in the specific disorders	97%
Physical therapy	
Regular assessments by a physical therapist (lower limb), occupational therapist (upper limb) and rehabilitation medicine specialist should be conducted to assess upper and lower limb function and provide support as needed	93%
The physical therapist could also assist in suggesting walking aids and other adaptations that may improve QoL	98%

Post-consensus comments by the SC to be taken into consideration

^a^Magnetic resonance imaging (MRI) can also be used to assess for spinal cord compression. The frequency may be reduced for older patients with stable imaging who do not display symptoms

^b^Tandem mass spectrometry may also be used to assess disease specific oligosaccharides [32]

^c^Echocardiogram (ECHO) should also be performed prior to any surgical procedure requiring general anaesthesia

^d^This topic was discussed in detail with the neurosurgical and orthopaedic colleagues in the SC group. It was their expert clinical opinion that flexion/extension MRI is not dangerous to perform within the hands of an experienced team. It is important that the range of motion (ROM), flexion and extension of the patient is evaluated while they are awake immediately before anaesthesia. The ROM during anaesthesia should not exceed the ROM as noted in the awake state, and should only be carried out after it is confirmed that there is no spinal cord compression. See Table 9 for guidance statements on spinal surgeries including spinal cord decompression

^e^Signs and symptoms for sleep apnoea (a type of sleep disordered breathing (SDB)) can be divided into nocturnal and daytime symptoms. Nocturnal symptoms include loud snoring, observed episodes of breathing cessation during sleep, abrupt awakenings accompanied by gasping or choking, and awakening with a dry mouth or sore throat. Daytime symptoms include excessive daytime sleepiness, morning headaches, difficulty concentrating during the day, personality and mood changes including depression or irritability, and high blood pressure. To identify presence of SDB, patients should be asked to report snoring and other signs and symptoms of SDB at every clinic visit

^f^Tympanometry is used to measure the volume of the ear canal/tympanic membrane movement and indirectly assess for fluid accumulation and opening of pressure equalising tubes

^g^Upper airway CT may also be useful to identify the area of the abnormality and possible cause of obstruction in patients with MPS VI

#### Disease-modifying interventions

##### ERT (galsulfase) in patients with MPS VI (Table 4)

**Table 4:** Guidance statement for galsulfase

Statement	Percentage consensus
Initiation of long-term ERT with galsulfase at a dose of 1 mg/kg/week by intravenous infusion is recommended in patients with MPS VI as soon as possible after a confirmed diagnosis Evidence Grade: B (level 2/3/4 studies)	89%

***Rationale and evidence base*** Galsulfase is a recombinant form of human lysosomal enzyme *N*-acetylgalactosamine 4-sulfatase, an enzyme that is deficient in patients with MPS VI. Treatment with galsulfase aims to transiently restore *N*-acetylgalactosamine 4-sulfatase activity, thereby preventing the accumulation of GAGs in lysosomal compartments of cells, which causes the clinical manifestations of MPS VI [40]. It is currently the only disease-specific treatment for MPS VI that is licensed and has been validated in clinical trials and long-term post-marketing surveillance studies [41–44]. Administration in patients with high baseline urinary GAG levels resulted in a statistically significant increase in height Z-score from pre-treatment baseline to last follow-up for those beginning treatment at 0–3, > 3–6, > 6–9, > 9–12, and > 12–15 years of age [44]. Galsulfase has been shown to improve endurance (as measured by the 6 min walk test [MWT], 12MWT and 3-min stair climb [3MSC]) [25, 40, 41, 45–49] and pulmonary function (as measured by increases in forced vital capacity [FVC] and forced expiratory volume [FEV_1_]), which may, in part, be attributed to growth in young patients [41]. Results suggest that if initiated early (in patients under 16 years of age), galsulfase also results in an improvement in growth velocity, although comparative data in patients who have not received ERT are limited [41–44]. When initiated early (in patients under 12 years of age), long-term treatment with ERT is effective in preventing the progression of cardiac valve abnormalities; however, the resultant effect on cardiac outcomes is equivocal [50, 51]. There is a trend for improvement in spleen and liver size [42, 52–54], facial dysmorphia [54], joint mobility and decreased pain [40, 52, 53, 55], and findings are suggestive of slowing of bone disease progression [56].

The most common adverse events reported in the galsulfase clinical studies include pyrexia, rash, pruritus, urticaria, chills/rigors, nausea, headache, abdominal pain, vomiting and dypsnoea. Serious adverse reactions included laryngeal edema, apnoea, pyrexia, urticaria, respiratory distress, angioedema, asthma and anaphylactoid reaction. Infusion-associated reactions (IARs), defined as adverse reactions occurring during galsulfase infusions or until the end of the infusion day, were observed in 33 (56%) of the 59 patients treated with galsulfase across five clinical studies [57, 58].

The safety and superior efficacy of galsulfase when administered from a young age has been demonstrated in several sibling-controlled studies [59–61]. However, most available data are from patients who initiate ERT later in the course of disease and additional studies are warranted to determine the long-term outcomes of galsulfase treatment when administered from an early age.

***Considerations prior to starting ERT*** Patient status, disease burden and ultimate prognosis at the time of diagnosis must be accounted for when deciding the timing for initiation of ERT. Initial administration of galsulfase should be performed (where possible) by a clinician with experience of metabolic disorders and in an infusion centre or hospital with the necessary facilities to effectively manage IARs/anaphylactic reactions, should this be required. Home infusion may be considered in regions where this is available; this decision should be made by both the physician and patient. Careful patient selection, good vascular access and a detailed management plan for IARs/anaphylaxis are essential for the success of this approach. Consideration should be given to the need for a totally implantable vascular access device (TIVAD) to facilitate long-term venous access for frequent or continuous administration of ERT. Patients and their families should be made aware of the benefits and risks of using such a device as outlined in two case series [62, 63].

***Considerations for monitoring response*** Baseline and follow-up assessments to measure treatment efficacy should be performed prior to and regularly after initiation of galsulfase. These should include GAG/dermatan sulphate concentration, endurance testing, upper limb function, respiratory function (if age-compatible), growth, height and weight, pain, activities of daily living (ADL) and quality of life (QoL). It is important to assess the life-long impact of galsulfase on an individual basis, as the benefits of treatment may not be consistent across all patients [43, 64].

***Considerations for managing specific adverse events*** Due to the potential for hypersensitivity reactions with galsulfase, patients who were treated with galsulfase in the clinical trial programmes received antihistamine premedication, with or without antipyretics, 30–60 min prior to the start of the infusion. Owing to concerns about the risk of hypersensitivity reactions with galsulfase, this approach is broadly followed in clinical practice; however, there is limited evidence in support of the necessity of premedication use. Patients should be closely observed for signs of anaphylaxis during and after administration of galsulfase and if suspected, hospital admission is advised. IARs are generally manageable by reducing the rate of administration or by temporary interruption of the infusion and the administration of additional antihistamines and antipyretics. Due to the risk of sleep apnoea in patients with MPS VI, use of a non-sedating antihistamine is recommended.

##### HSCT in patients with MPS VI (Table 5)

**Table 5:** Guidance statements for HSCT

Statement	Percentage consensus
With consideration of the associated risks of morbidity and mortality associated with this procedure, HSCT may be an option for patients with MPS VI who have a matched related donor (or unrelated donor), or cord blood graft^a^ Evidence Grade: C (consistent with level 4 studies and extrapolations from level 3 studies)	86%
Due to the risk of mortality, it is critical that HSCT is only performed in an institution with a multidisciplinary team experienced in the care of patients with MPS VI Evidence Grade: D (level 3/4 studies with inconsistent risk/benefit results)	91%

Post-consensus comments by the SC to be taken into consideration

^a^HSCT may be an option for patients with MPS VI who have a matched related donor (preferably not a carrier) or a well-matched adult unrelated donor

***Rationale and evidence base*** The strongest data supporting the role of HSCT as a treatment for MPS is derived from subtypes other than VI; specifically, MPS IH (Hurler disease) [65, 66]. HSCT is currently the standard of care in patients with MPS IH because of its associated improvement in central nervous system (CNS) disease, which is not effectively treated by ERT [66–70]. The incidence of hydrocephalus and cervical stenosis in patients with MPS IH is reported to be lower in those treated with HSCT versus ERT [71]; however, as cognitive deficits similar to those seen in patients with MPS IH have not been observed in patients with MPS VI, it is unclear whether HSCT would be an effective treatment for MPS VI.

Evidence supporting the use of HSCT in patients with MPS VI is currently lacking, being based on a small number of case studies and results from non-randomised follow-up studies [72–76]. Evidence from case studies in patients with MPS VI suggests that HSCT increases the enzymatic activity of *N*-acetylgalactosamine 4-sulfatase in circulating white blood cells, and has a positive effect on joint mobility, ENT and cardiac manifestations, movement, QoL, as well as reducing facial dysmorphism [72]. Normalisation of urinary GAG and dermatan sulphate levels and slowed disease progression have been observed in patients with mild MPS VI phenotypes, with variable outcomes observed in visual acuity [77, 78]. There are limited survival data post-HSCT in patients with MPS VI. Although now somewhat dated, results of a retrospective study of HSCT outcomes in patients with MPS VI (for transplants performed between 1982 and 2007) reported a cumulative incidence of acute graft versus host disease at 100 days of 36% and a probability of survival of 78% at 100 days and 66% at both 1 and 3 years [76]. The collaborative efforts of the transplant community have resulted in a decline of the risks associated with transplant across MPS [79, 80]; however, mortality rates still vary between centres, and serious risks, including death, remain. It is the opinion of the expert SC that risks may be higher in less experienced centres and it is therefore critical that HSCT is only performed in centres dedicated to transplant with access to an MDT with experience of managing patients with MPS. Matched donors should preferably not be carriers for MPS VI, and unrelated donors should be well-matched.

Overall, the expert consensus was that the risk–benefit profile of HSCT in patients with MPS VI is less clear than in other types of MPS, and further research, specifically, a well-designed comparative study of HSCT and ERT in patients of similar age and disease severity is needed to better understand the long-term efficacy and safety of HSCT in patients with MPS VI.

***Considerations for monitoring response*** It is important to consider the life-long impact of HSCT on an individual basis, as the benefits of treatment manifest differently between each patient. Assessments should be performed prior to, and at least annually, after HSCT to measure the impact of transplantation on disease progression. Assessments should include: endurance testing, cardio-respiratory function (owing to the significant cardiac symptoms in patients with MPS VI), and measurement of height and weight. Follow-up should also include assessment of orthopaedic, ophthalmic, ENT, neurological and endocrine function. Data should be collected and shared in a manner that can advance the understanding of the risks and benefits of HSCT.

#### Interventions to support respiratory and sleep disorders

##### Continuous positive airway pressure (CPAP), non-invasive positive pressure ventilation (NIPPV), oxygen supplementation and hypercapnia monitoring (Table 6)

**Table 6:** Guidance statements for CPAP, NIPPV, oxygen supplementation and hypercapnia monitoring

Statement	Percentage consensus
CPAP therapy is recommended for patients with MPS VI who display the presence of obstructive sleep apnoea (OSA) which persists after tonsillectomy and/or adenoidectomy Evidence Grade: B (extrapolations from level 1 studies)	100%
NIPPV therapy is recommended for patients with MPS VI who display nocturnal hypoventilation and are unresponsive to CPAP, or display daytime hypoventilation with increased PaCO_2_ and/or serum HCO_3_ levels Evidence Grade: B (extrapolations from level 1 studies)	94%
Oxygen supplementation is recommended for patients with MPS VI who display sleep apnoea with nocturnal hypoxemia and who do not tolerate CPAP or NIPPV masks Evidence Grade: B (extrapolations from level 1/3/4 studies)	83%
Patients with MPS VI should be monitored for development of hypercapnia after starting oxygen therapy with measurement of PaCO_2_ and/or serum HCO_3_ Evidence Grade: D (level 5, expert clinical opinion)	97%

***Rationale and evidence base*** Upper airway obstruction leading to obstructive sleep apnoea [81] is a common morbidity in patients with MPS VI and can significantly affect functional status and QoL [14, 82]. Typical features of MPS VI include upper and lower airway obstruction and restrictive pulmonary disease that occur from a variety of anatomic and functional abnormalities [83]. Upper airway obstruction is attributable to cranial abnormalities, a short neck and progressive deposition of GAGs in the tissues surrounding the supraglottic upper respiratory tract, while lower airway obstruction reflects GAG deposition in the airway walls with resultant tracheal and bronchomalacia. Lung volume and chest expansion are further limited by short stature, chest wall deformities and abdominal organomegaly [84].

A comprehensive review of the evaluation and treatment options for sleep disordered breathing (SDB) in MPS has been recently published [85]. Continuous positive airway pressure (CPAP) prevents upper airway collapse during inspiration and is the mainstay of treatment for OSA in the general population with beneficial effects on blood pressure, cardiac events, mortality and QoL [86]. Recent studies have demonstrated effectiveness of CPAP in patients with MPS, showing improvement in pulmonary hypertension and cardio-respiratory failure [87–89]. An alternate form of therapy is required for patients who demonstrate either persistent OSA despite CPAP or hypoventilation during sleep. Non-invasive positive pressure ventilation (NIPPV) provides an increased pressure during the inspiratory phase of breathing to augment ventilation.

Supplemental oxygen can be prescribed for individuals that demonstrate persistent nocturnal oxygen desaturation and for patients who do not tolerate therapy with CPAP or NIPPV. Caution is required when prescribing oxygen because of the known complication of suppressing respiratory drive and arousal from sleep with potential for either worsening a pre-existing hypercapnia or inducing onset of hypercapnia is susceptible patients.

***Considerations for management*** SDB can be managed by application of CPAP which delivers air at an elevated pressure via a mask that fits around the nose and/or mouth; however, consideration should be given to facial abnormalities that can make mask fitting difficult. Patients should be monitored to ensure they do not develop sustained hypoventilation. Vaccinations against respiratory pathogens causing influenza and pneumococcus infections are recommended.

#### Anaesthetics and surgical interventions

##### Use of anaesthesia in patients with MPS VI (Table 7)

**Table 7:** Guidance statements for anaesthesia

Statement	Percentage consensus
Pre-, intra- and post-operative care (until extubation is complete) for all procedures requiring general anaesthesia, conscious or deep sedation, should be supervised by an anaesthetist with experience in treating patients with MPS and/or complex airway management. In addition, the anaesthetist should have access to Intensive Care support and be surrounded by an experienced team capable of performing emergency tracheotomy if required Evidence Grade: C (level 3/4 studies)	98%
A full assessment of the risks and benefits should take place with the patient and family prior to any procedure. All pre-operative information should be made available to allow decision making Evidence Grade: C (level 4 study and extrapolation from level 3 study)	100%
ENT respiratory, cardiac, and radiological assessment should be performed prior to any procedure requiring anaesthesia Evidence Grade: C (level 3 study and extrapolation from level 3 study)	93%
It is critical to maintain a neutral neck position during all surgeries, and during intubation and extubation to avoid paralysis^a^. Strongly recommend the use of techniques that allow maintenance of the neutral neck position, including use of laryngeal mask airway (LMA) for shorter procedures, or intubation with a video laryngoscope or fibreoptic intubation Evidence Grade: C (level 3/4 studies)	87%
Pre-operative and intra-operative measures to avoid hypotension should be adopted during all surgical procedures in patients with MPS VI to maintain spinal cord perfusion and therefore protect spinal cord function Evidence Grade: D (expert clinical opinion)	98%
Intra-operative neurophysiological monitoring (including somatosensory evoked potentials [SSEP], electromyography [EMG] and motor evoked potentials [MEP]) is strongly recommended during all spinal surgeries and other potentially lengthy or complicated procedures, including those that require manipulation of the head and neck Evidence Grade: D (limited published evidence)	94%
For other surgeries and procedures, neurophysiologic monitoring should be considered based on pre-existing risk for spinal cord compression and instability, need for spine manipulation, possibility of hemodynamic changes and blood loss, or extended length of time Evidence Grade: D (limited published evidence)	94%
Intrathecal and epidural techniques should be used with extreme caution in patients with MPS VI, due to the anatomical challenges of very short stature, as well as spinal abnormalities causing insertion problems and unpredictability of spread of local anaesthesia. However, these techniques may be considered to avoid general anaesthesia in a high-risk situation or during pregnancy Evidence Grade: D (expert clinical opinion)	88%

Post-consensus comments by the SC to be taken into consideration

^a^It is critical to maintain a neutral neck position to avoid any spinal cord injury

***Rationale and evidence base*** Patients with MPS VI will likely require anaesthesia for multiple surgical interventions and investigations to manage their disease [90, 91], but are considered high-risk for anaesthesia due to potential airway difficulties with mask ventilation and/or endotracheal intubation. Other risk factors include the presence of narrow airways due to adenotonsillar hypertrophy, macroglossia and deformity of the lower airway, skeletal abnormalities, pulmonary disposition and cardiac and neurological impairment [14, 92, 93]. Intubation and extubation can be challenging due to restricted mouth opening, short neck length with a limited range of motion, airway abnormalities already mentioned, micrognathia, subglottic narrowing, and atlanto-axial instability due to odontoid hypoplasia and ligamentous laxity [94–96]. Although hypothetical, poor perfusion related to arterial narrowing and reduced foramina diameters secondary to dysostosis should be anticipated by an anaesthetist, be appropriately monitored with arterial lines, and supported in the near-normal range during procedures.

Adverse events (including fatalities and paralysis) occurring during anaesthesia of patients with MPS have been reported in the literature [97]. Although data on peripheral nerve blocks are lacking, this approach may be considered, and use of ultrasound technology can assist successful performance of these procedures and may allow avoidance of general anaesthesia in selected patients. Perioperative neurophysiological monitoring is recommended to prevent significant complications in this high-risk population; however, availability worldwide is extremely variable.

***Considerations for anaesthesia*** Due to the risk of upper airway obstruction, pre-operative sedative premedication should be used with caution in patients with MPS VI and only with appropriate monitoring. Assessment of the upper and lower airways anatomy (for example, a pre-operative flexible nasopharyngolaryngoscopy and three-dimensional computerised tomography (CT) scan of the trachea, where feasible), cardiac function (including an ECG and echocardiogram), and potential cervical spine instability and compression, should be performed and function as baseline evaluation prior to any procedure that requires sedation or anaesthesia. This needs to be repeated at a future date with increase in age and weight. Similarly, MRI scans of the spine in a neutral position or a flexion/extension X-ray of the spine can be performed to assess the risk of spinal cord compression and instability (flexion/extension X-ray measures instability only). Flexion/extension imaging of the cervical spine prior to anaesthesia is required to assess atlantoaxial instability. The frequency of imaging should be dependent on both the patient’s age and clinical condition. To avoid spinal cord injury, sensory injury with dysesthetic pain, and/or loss of proprioception, it is critical to maintain a neutral neck position during all surgeries, including intubation and extubation. The aim is to avoid spinal cord injury which can lead to paralysis. When possible, intubation should be completed while patients are breathing spontaneously, and the use of paralytic agents should be avoided such that spontaneous breathing is maintained until intubation is completed successfully. Use of a smaller endotracheal tube size is usually necessary and often critical, to avoid intraoperative swelling of the airway and enable successful extubation. Where possible, patients should be extubated in the operating room (OR) and asked to demonstrate movement of all extremities. If safe intubation cannot be achieved, tracheostomy may be considered electively prior to prolonged surgery, or to facilitate post-operative care. If the patient is awake or breathing spontaneously, the option of delaying the surgery after failed intubation should be considered. Mean arterial pressure should be maintained to maximise perfusion of the spinal cord and reduce the risk of spinal cord injury. Displacing the tongue anteriorly prior to intubation by manual retraction using a ring forceps or a piece of gauze may help to access the larynx in children with MPS VI [94]. Intensive care management is often not required but may be necessary for complicated or prolonged procedures requiring post-operative ventilation or peri-operative tracheostomy. If ventilated via an endotracheal tube, it is best to aim for early extubation to minimise swelling of the airway. When clinically indicated, maintenance of intubation overnight following the procedure may be considered to allow resolution of any airway swelling. Extubation should be performed by an experienced anaesthetist who can assess the airway before extubation and if necessary reintubate in the intensive care unit (ICU) or OR. Wherever possible, alternative techniques (e.g. peripheral nerve block under light sedation) should be considered to avoid general anaesthesia and the associated risks thereof. However, the surgical team should always be prepared to perform general anaesthesia when required.

***Considerations after surgery*** Intraoperative use of steroids to reduce airway oedema is standard and the use of post-operative treatment may also be necessary for 24 h. Standard treatment for patients with upper airway obstruction should be available, including NIPPV, CPAP, and continuous monitoring of respiratory and cardiac function. ICU stays are not mandatory for all patients after all surgeries and should only be used when needed; nonetheless, availability of ICU facilities for management of complications, is critical. Intensive monitoring is required for 24–48 h post-surgery because of the potential complications of oral secretions, thoracic cage stiffness and heart and lung failure, which can include apnoea, laryngospasm, bronchospasm, cyanosis and respiratory failure.

##### Limb surgeries in patients with MPS VI (Table 8)

**Table 8:** Guidance statements for hip replacement, hip reconstruction and growth modulation surgery

Statement	Percentage consensus
Hip replacement can be considered in adult patients with MPS VI who exhibit hip pain, reduced walking and endurance related to hip disease, as well as abnormal radiographic findings Evidence Grade: D (limited published evidence)	100%
Hip reconstruction is not routinely indicated, but may be considered in paediatric patients with MPS VI who exhibit hip pain, reduced walking and endurance related to hip disease, as well as abnormal radiographic findings Evidence Grade: D (limited published evidence)	92%
Growth modulation surgery is recommended in patients with MPS VI who have signs of genu valgum and should be performed as early as possible during the period of growth Evidence Grade: D (limited published evidence)	87%

***Rationale and evidence base*** MPS VI is characterised by profound skeletal dysplasia with cervical spinal canal stenosis, hip abnormalities and genu valgum [98]. Hip problems can lead to severe disability [23]. Patients with MPS VI have progressive musculoskeletal involvement, and numerous orthopaedic interventions are usually required to prevent deformity, improve function and reduce pain. Evidence from a prospective follow-up study showed that clinically significant hip abnormalities develop in all patients with MPS VI from very early on in life, starting with deformities of the os ilium and acetabulum. Femoral head abnormalities occur later and are most likely due to altered mechanical forces in combination with epiphyseal abnormalities due to GAG storage, however, the final shape and neck shaft angle differs significantly between individual patients and is difficult to predict [23]. Case studies based on two patients with MPS I with skeletal dysplasia and thoracic kyphosis showed a rare complication of the spinal cord injury. This reiterated the importance of careful pre-operative assessments, including MRI of the spine, to reduce the risk of spinal cord injury [99].

Guided growth techniques for correction of genu valgum are not widely reported in patients with MPS VI; however, the indications for this approach need reconsideration in the era of ERT. A recent report of guided growth surgery conducted in two children with MPS VI while receiving ERT suggest that that this may be a useful approach to correcting knee deformities [98].

***Patient selection for intervention*** Most patients with MPS VI will have abnormal radiographic findings; therefore, hip surgery should only be considered in patients who are symptomatic, as determined by presence of hip pain resulting in much reduced mobility and endurance. Growth modulation surgery should be initiated as soon as the deformity is observed, or if the tibial-femoral angle is > 15 degrees. For optimal results, it should be performed early during the period of growth, due to the deceleration in growth that occurs as the skeleton matures [100], however expert clinical opinion varies regarding the ideal age to perform the surgery. The period following ERT commencement may also be a good time to perform growth modulation surgery. Before orthopaedic intervention, morbidity and mortality risks, pain level, optimal timing and patient preference should be considered on a case-by-case basis.

Currently there is no hand surgical intervention that can be recommended to improve the weakness of the grip but maintain vital flexibility for transfer and adequate ADL. External custom-made splints can be worn to help with certain tasks e.g. heavy lifting. Occupational therapists are vital to help with ADL including providing gadgets to perform necessary tasks. Patients with weak grip can learn to adapt to master necessary ADL.

***Considerations for surgery*** All surgeries should be supervised by an anaesthetist with experience in treating MPS and/or complex airway management (refer to the anaesthetics recommendations). Limb surgeries should be performed by an orthopaedic surgeon with a basic understanding of MPS and of the clinical presentation, musculoskeletal abnormalities, and radiographic findings associated with this group of disorders. An overnight hospital stay is recommended following hip surgery to allow access to intensive care, should this be needed, although this may not be necessary for surgeries such as hemi-epiphysiodesis. Long-term, intensive physical therapy is recommended post-surgery to enhance recovery, and assessment should be performed regularly as patients may require repeated surgeries/interventions. The primary goal of limb surgery is not to improve or restore joint range of motion (ROM), but rather to reduce pain or improve mobility. Goniometer measurements performed by a physiotherapist/occupational therapist/rheumatologist may be useful but may not be available in all centres.

##### Spinal surgeries in patients with MPS VI (Table 9)

**Table 9:** Guidance statements for decompression of the spinal cord, spinal stabilisation and thoracolumbar kyphoscoliosis

Statement	Percentage consensus
Decompression of the spinal cord is recommended in patients with MPS VI who have evidence of spinal cord compression based on clinical and radiographic^a^ findings Evidence Grade: D (limited published evidence)	97%
Spinal stabilisation of the craniocervical junction with either cervical fusion or occipital-cervical fusion is recommended in patients with MPS VI who have evidence of significant instability Evidence Grade: D (expert clinical opinion)	100%
Correction of thoracolumbar kyphoscoliosis is recommended in patients with MPS VI who present with progressive radiographic changes, intractable pain and clinical deterioration as defined by gait, lung function and changes in the degree of kyphosis Evidence Grade: D (limited published evidence)	97%

Post-consensus comments by the SC to be taken into consideration

^a^The SC would like to clarify that neuroimaging is a required radiologic procedure to define compression. MRI is best to image the brain and spinal cord for this indication. Decompression of the spinal cord is recommended in patients who have evidence of spinal cord compression and risk of injury based on clinical and neuroimaging findings

***Rationale and evidence base*** Skeletal abnormalities are early and prominent features of MPS VI and are potentially debilitating and life-threatening [14, 53]. Medical therapies for the management of MPS have a limited effect on the development of skeletal deformities; therefore, early surgical intervention is important to manage disease progression [100, 101]. The frequency of spinal cord compression and the success of surgical intervention has been reported by a clinical surveillance programme [102]. Routine neurological history, examination and appropriate imaging should be part of standard care to detect early compromise of the spine.

***Patient selection for intervention*** Indications for surgery include cervical spine cord compression as determined by clinical symptoms (including weakness, numbness, paraesthesia, and gait difficultly) or radiographic and MRI findings (including plain radiographic findings suggestive of stenosis and instability and MRI findings of extradural stenosis, cord compression, myelomalacia and instability). Physicians should consider the timing of such surgery in line with the need for cardiac valve replacement, as the latter procedure could subsequently commit the patient to lifetime anticoagulation therapy.

***Considerations for surgery*** Spinal surgeries should be performed by a neurosurgeon and/or spinal surgeon with a basic understanding of MPS and of the clinical presentation, musculoskeletal abnormalities, and radiographic findings associated with this group of disorders.

##### Ophthalmic surgery in patients with MPS VI (Table 10)

**Table 10:** Guidance statement for corneal transplantation

Statement	Percentage consensus
Corneal transplantation can be considered for patients with MPS VI who have significant visual loss attributed to corneal opacification Evidence Grade: B (extrapolations from level 1/3/4 studies)	100%

***Rationale and evidence base*** Corneal opacification leading to reduced visual acuity is a common feature in patients with MPS VI and does not appear to be influenced by ERT. Other ophthalmic findings in patients with MPS VI include high hyperopia, increased corneal thickness, optic nerve abnormalities, ocular hypertension, glaucoma, and rarely, retinopathy [103, 104]. Clear corneal grafts can be achieved for patients with MPS VI who have corneal clouding, and may result in improvements in visual acuity [105]. Rejection episodes have been reported following corneal transplantation in patients with MPS VI; however, it should be noted that most occurred in those who were transplanted at a younger age (13–28 years old) [105, 106]. Generally, the recurrence of corneal deposits has not been reported in patients with MPS VI [105, 106].

***Patient selection for intervention*** Corneal transplantation can be considered if corneal clouding is of such severity that it causes significant loss of vision and impacts QoL. The decision to perform corneal transplantation should be made on a case-by-case basis and should only be considered once retinopathy and optic nerve abnormalities have been assessed using electrodiagnostic (including electroretinography and visual evoked potentials) and have been excluded as a significant contributing factor to the loss of vision. The choice of surgical technique for corneal transplantation (deep anterior lamellar keratoplasty [DALK] or penetrating keratoplasty [PK]) should be made on a case-by-case basis. There is some evidence from the general population to suggest that rejection is more likely to occur following PK than DALK [106–108]; as such, DALK should be considered as the first approach in patients with MPS VI.

***Considerations for intervention (e.g. if surgery)*** Monitored anaesthesia care with appropriate sedation and support with nasal CPAP/NIPPV may be used when performing eye surgery in patients with MPS VI. Signs of rejection require prompt ophthalmic assessment to prevent graft failure. Following corneal transplantation, long-term topical treatment is needed, as is regular (annual) ophthalmic assessment to determine the health of the corneal graft, assess for recurrence of corneal deposits and astigmatism control. Follow-up is also required to monitor for optic neuropathy due to raised intracranial pressure. This can be indicated by a reduction in visual acuity, new onset of visual field defect, abnormal pupil reactions, new onset of optic nerve swelling or (more commonly) optic atrophy and visual evoked potential abnormality.

##### Carpal tunnel decompression in patients with MPS VI (Table 11)

**Table 11:** Guidance statements for decompression of the median nerve, tenosynovectomy and pulley release

Statement	Percentage consensus
Decompression of the median nerve and tenosynovectomy of all flexor tendons in the carpal tunnel is recommended in patients with MPS VI who display flexion contractures and distal interphalangeal (DIP) joints and/or proximal interphalangeal (PIP) joints (clawing), as well as clinical symptoms of hand pain and/or numbness in the thumb to middle finger, and in patients with positive nerve conduction studies Evidence Grade: C (level 4 studies)	89%
A1 and A3 pulley release is recommended in patients with MPS VI who display obvious trigger finger Evidence Grade: C (level 4 studies)	94%

***Rationale and evidence base*** Carpal tunnel syndrome (CTS) is a common condition in patients with MPS VI, which if left untreated, can lead to loss of nerve function. Trigger finger can also present in association with CTS in patients with MPS VI. Surgical decompression of CTS, especially when performed early, has reduced signs and symptoms of compressive myelopathy and improves the chance of preserving hand function [109, 110]. Evidence, albeit from limited case studies in patients with MPS, suggests that clinical improvements in hand/motor function, dexterity and spontaneous hand function are observed after carpal tunnel decompression (CTD), which are coupled with reduction in long-term hand pain and cessation of night pain [109]. The preservation of hand function requires a combination of steps including decompression of the median nerve, tenosynovectomy and if necessary, an A1 and A3 pulley release rather than decompression alone. There are no data to suggest that an approach using tenosynovectomy alone is beneficial compared with tenosynovectomy plus complete epineurectomy; however, in rare cases, epineurotomy may be useful to achieve more effective decompression. Recurrence is frequent in older patients therefore repeated surgery may be indicated.

***Patient selection for intervention*** CTD should be performed in patients who display restriction of hand function, including increased flexion contracture of the PIP and DIP joints, indicative of an increase in ‘scarred’ and tethered subsynovial connective tissue around the flexor tendons in the carpal canal. Clinical symptoms of hand pain/numbness include night pain, biting and/or slapping of fingers and pain or numbness in the thumb, index or middle finger. It is important to note that if a patient reports numbness in all fingers it is unlikely to be CTS, and pain in the little finger is suggestive of compression higher up than the carpal canal. Neurophysiological tests should be performed to rule out cervical compression before initiating surgery.

***Considerations for surgery*** CTD should be performed by a hand surgeon with a basic understanding of MPS and of the clinical presentation associated with this group of disorders. In most cases, CTD may be performed with regional block plus sedation, with or without the use of LMA.

***Considerations for post-surgery monitoring*** Regular post-surgical physical therapy may facilitate maintenance of increased hand movement. All patients who undergo CTD surgery should be assessed (pre- and post-surgery) for QoL, improvement in ROM and self-reported change in function.

##### Cardio-thoracic surgery in patients with MPS VI (Table 12)

**Table 12:** Guidance statements for cardiac valve replacement, left ventricular apical aneurysms and tracheostomy

Statement	Percentage consensus
Cardiac (aortic, mitral) valve replacement should be considered in patients with MPS VI who display symptomatic and severe valve stenosis or regurgitation Evidence Grade: C (level 4 studies)	100%
Left ventricular apical aneurysms occur rarely in patients with MPS VI, but should be resected whenever possible Evidence Grade: D (limited published evidence)	85%

***Rationale and evidence base*** Cardiac valve disease, which occurs as a result of valve thickening from deposition of storage material, occurs commonly in patients with MPS VI [111] and is a major cause of morbidity and mortality. Cardio-thoracic surgery in patients with MPS VI can be challenging because of small patient size and associated skeletal and pulmonary co-morbidities. Evidence from several case reports and small case series suggests that replacement of the aortic and mitral valves, either singly or in combination, is feasible in patients with MPS VI [112–116]. Successful resection of a left ventricular apical aneurysm, a rare complication of MPS VI, has been reported [117], but successful balloon dilation of aortic or mitral valves has not.

***Patient selection for intervention (refer to the recommendations for routine assessments and monitoring)*** The performance and interpretation of findings of echocardiography should be completed by individuals familiar with the expected pathological findings in patients with MPS VI. Valve replacement decisions should be based on current European/American (AHA) guidelines [118, 119] in conjunction with assessment of existing co-morbidities, operative risk and rehabilitation potential. Trans-catheter aortic valve replacement may be appropriate for some patients with MPS VI. The Ross procedure is contraindicated in patients with systemic valvular disease. Small valve annulus may preclude valve replacement with currently existing mechanical and bio-prosthetic cardiac valves.

***Consideration for surgery*** Cardiac surgery in patients with MPS VI should be performed in a centre of excellence with a team experienced in both managing patients with MPS and performing high-risk valve replacement surgery. When possible, an anaesthetic specialist with experience in managing patients with MPS should assist the cardiac anaesthetic team during pre-operative assessment in formulating an MPS-related anaesthesia care plan. Similarly, the anaesthetic care plan for cardiac catheterisation should be formulated jointly by the cardiologist and anaesthetic care team specialist. Airway care, including the need for tracheostomy, should be assessed on a case-by-case basis.

##### Ear, nose and throat surgery in patients with MPS VI (Table 13)

**Table 13:** Guidance statements for tonsillectomy and/or adenoidectomy, tracheostomy and insertion of ventilation tubes

Statement	Percentage consensus
Tonsillectomy and/or adenoidectomy is recommended in patients with MPS VI who display upper airway obstruction, recurrent otitis media, snoring and/or OSA, as early as possible following diagnosis without waiting for disease progression Evidence Grade: C (level 2/3/4 studies)	91%
Tracheostomy is recommended in patients with MPS VI who exhibit severe upper airway obstruction that cannot be treated by an alternative approach, and in patients with severe sleep apnoea that is not treatable by CPAP or tonsillectomy and/or adenoidectomy Evidence Grade: D (limited published evidence)	95%
Insertion of ventilation tubes is recommended for patients with MPS VI with otitis media with effusion and/or recurrent otitis media to maintain hearing and/or prevent recurrent acute otitis media Evidence Grade: D (limited published evidence)	96%

***Rationale and evidence base*** ENT manifestations are common in patients with MPS VI and often involve hearing disorders, otitis media and upper airway obstruction [14]. Permanent hearing loss is common and is believed to be conductive and neurosensory in nature [14, 120]. Results from two non-randomised studies revealed that ENT surgery reduced hypoacusia, otitis media, incidence of upper respiratory tract infections, occurrence of OSA and need for Type B tympanograms. QoL was also reported to be improved in some patients [82, 121]. The results from another non-randomised study suggest that tonsillectomy and/or adenoidectomy significantly improves post-operative sleep apnoea in MPS patients [82]. Development of secondary haemorrhage is a serious risk associated with tonsillectomy and/or adenoidectomy in patients with MPS as difficult intubations are common in these patients and can be fatal [82, 122]. Evidence from a case series shows that insertion of ventilation tubes can improve air and bone conduction and the air-bone gap in patients with MPS VI [39]. Advanced surgical options such as uvulopalatopharyngoplasty (UPPP), mandibular advancement surgery and tongue reduction are currently experimental. While one member of the SC had experience, currently, there is not enough evidence from which to derive any recommendations about the use of these invasive procedures in patients with MPS VI.

***Considerations for surgery*** Vaccinations against respiratory pathogens causing influenza and pneumococcus infections are recommended to prevent pneumonia. Insertion of ventilation tubes should be performed according to the guidelines for the general paediatric population [123, 124]. Patients who have had tonsillectomy and/or adenotonsillectomy should be observed as in-patients and may need to remain in hospital preferably in intensive care in the early post-operative period to monitor airway patency. They may need to remain hospitalised for additional days to allow close monitoring for possible haemorrhage and other complications. Patients with ventilation tubes should be assessed every three months and if improvement in hearing is absent, a post-operative audiologic assessment should be performed. The anaesthesia plan should be discussed jointly between the otolaryngologist and anaesthesia care team and precautions should be taken to prevent spinal cord compression during surgical procedures. However, before performing tonsillectomy, children with MPS should be referred by the clinician for polysomnography for sleep [125].

## Discussion

Where evidence is scarce, systematic approaches are required to ensure the evidence-base is as extensive as possible. This programme involved a validated systematic approach to the development of guidance statements and the resulting publication therefore addresses this unmet need by creating a robust, holistic set of recommendations for healthcare professionals managing patients with MPS VI. A detailed discussion on the development of the guidance methodology, strengths and limitations of the programme, future directions, facilitators/barriers to support the application of the guidance including cost considerations, and conclusions is provided in the discussion of the companion article of this publication (*Recommendations for the management of MPS IVA: systematic evidence- and consensus-based guidance*).

## Conclusions

This manuscript provides robust evidence- and consensus-driven guidance for the management of patients with MPS VI. The guidance is intended for use by healthcare professionals that manage the holistic care of patients with MPS with the intention to enhance patient quality of life and improve clinical- and patient-reported outcomes. It recognised that the guidance provided represents a point in time and further research is required to address current knowledge/evidence gaps. The SC recommends that this guidance is reviewed and updated within 5 years, or sooner if there are significant changes to medical practice.

## Additional files

Additional file 1: Methodology. Further information regarding methodology, including: defining clinical questions using the P.I.C.O methodology, the search strategy recording form, results of the systematic literature review according to PRISMA, the Oxford Centre for Evidence-based Medicine criteria and the AGREE II evaluation. (DOCX 69 kb)

Additional file 2: Oxford CEBM grading for MPS IVA: Tables detailing the evidence levels given to each reference supporting the MPS IVA guidance statements and the Evidence Grades applied to each guidance statement. Evidence levels were assessed using the Oxford Centre for Evidence-based Medicine and were based on the quality of evidence of each reference. For each guidance statement, an overall Evidence Grade was applied, based on the evidence levels of the supporting references. (DOCX 41 kb)

Additional file 3: Oxford CEBM grading for MPS VI. Tables detailing the evidence levels given to each reference supporting the MPS VI guidance statements and the Evidence Grades applied to each guidance statement. Evidence levels were assessed using the Oxford Centre for Evidence-based Medicine and were based on the quality of evidence of each reference. For each guidance statement, an overall Evidence Grade was applied, based on the evidence levels of the supporting references. (DOCX 41 kb)

Additional file 4: Modified-Delphi voting Round 1. Full results of the first round of the modified-Delphi voting, which was used to demonstrate consensus of the guidance statements. (DOCX 151 kb)

Additional file 5: Modified-Delphi voting Round 2. Full results of the second round of the modified-Delphi voting, which was used to demonstrate consensus of the guidance statements. (DOCX 41 kb)
