# Supplementary material for: Recommendations for the management of MPS VI: systematic evidence- and consensus-based guidance
Source: Orphanet J Rare Dis. 2019 May 29;14:118. doi: 10.1186/s13023-019-1080-y (PMC6541999; doi:10.1186/s13023-019-1080-y)
Supplement: Supplementary file 1 — Methodology. Further information regarding methodology, including: defining clinical questions using the P.I.C.O methodology, the search strategy recording form, results of the systematic literature review according to PRISMA, the Oxford Centre for Evidence-based Medicine criteria and the AGREE II evaluation. (DOCX 69 kb) [file 13023_2019_1080_MOESM1_ESM.docx]

# Additional File 1: Methodology

**Appendix 1a. Defining clinical questions using the P.I.C.O methodology**

| **Patients** | **Interventions** | **Comparison** | **Outcomes** |
| --- | --- | --- | --- |
| - Paediatric and adult patients with a confirmed diagnosis of MPS IVA - Paediatric and adult patients with a confirmed diagnosis of MPS VI | **Disease-modifying interventions**   - ERT - HSCT   **Interventions to manage symptoms**   - Respiratory and sleep disorders  (e.g. continuous positive airway pressure [cPAP]/non-invasive positive pressure ventilation [NIPPV], oxygen supplementation and hypercapnia monitoring) - Anaesthesia - Surgical procedures:   - Spinal surgery (e.g. spinal cord decompression, spinal instability stabilization and fusion, spinal deformity correction)   - Limb surgery (e.g. hip reconstruction and/or total hip replacement, total knee replacement, guided growth procedures/hemiepiphysiodesis [ankle joint], osteotomy around the knee, carpal tunnel decompression)   - Ear-nose-throat (ENT) surgery (e.g. tonsillectomy and adenoidectomy [T&A] tracheostomy and insertion of ventilation tubes)   - Cardiac surgery (e.g. heart valve replacement)   - Ophthalmic surgery (e.g. corneal transplantation) | - No treatment - No surgical intervention | Note that the domains below will cover clinician- and patient-reported outcomes:  **Physical function/performance:**   - Survival/mortality - Growth/skeletal involvement - Mobility/balance - Grip/reach - Breathing - Hearing - Speech - Vision - Chewing/swallowing - Heart function - Energy - Sleep quality - Reproduction   **Mental/emotional outcomes**   - Mood - Quality of life - Pain   **Socioeconomic outcomes:**   - Social/peer relationships - School/work - Healthcare utilization (hospitalization and surgical interventions) - Compliance with therapy |

**Appendix 1b. Search strategy recording form**

| **Database** | **Date of search** | **Limits** | **Search terms/search strings** | **Number of articles** | **Notes (please provide reasons for amends to search terms)** |
| --- | --- | --- | --- | --- | --- |
| PubMed | 13/07/2017 | None | (mucopolysaccharidos* OR mucopolysaccharidoses[MeSH Terms] OR Morquio A syndrome OR Morquio syndrome OR Maroteaux-Lamy syndrome OR mucopolysaccharidosis IVA OR mucopolysaccharidosis VI) AND (carpal tunnel syndrome OR Vascular Access Devices[MeSH Terms] OR totally implantable vascular access device OR cardiac OR valve replacement OR ophthalmic surgery OR corneal transplant OR dental procedures OR tooth extraction OR occipito OR Surgical Procedures, Operative[MeSH Terms] OR surgery[MeSH Terms] OR hematopoietic stem cell transplant* OR HSCT OR bone marrow transplant OR BMT OR hemiepiphysiodesis OR spinal cord compression OR spinal surgery OR hip replacement OR hearing aid[MeSH Terms] OR hearing [MeSH Terms] OR respiration, artificial[MeSH Terms] OR tracheostomy OR orthopedic surgery OR anesthesia OR enzyme replacement therapy[MeSH Terms] OR enzyme replacement therapy OR ERT OR tonsillectomy OR adenoidectomy OR GALNS protein, human[Supplementary Concept] OR galsulfase[Supplementary Concept] OR vimizim OR naglazyme OR elosulfase alfa[Title/abstract] OR Treatment Outcome [MeSH Terms] OR clinical outcomes) | 2270 |  |
| Web of Science | 13/07/2017 |  | (mucopolysaccharidos* OR mucopolysaccharidoses[MeSH Terms] OR Morquio A syndrome OR Morquio syndrome OR Maroteaux-Lamy syndrome OR mucopolysaccharidosis IVA OR mucopolysaccharidosis VI) AND (carpal tunnel syndrome OR Vascular Access Devices[MeSH Terms] OR totally implantable vascular access device OR cardiac OR valve replacement OR ophthalmic surgery OR corneal transplant OR dental procedures OR tooth extraction OR occipito OR Surgical Procedures, Operative[MeSH Terms] OR surgery[MeSH Terms] OR hematopoietic stem cell transplant* OR HSCT OR bone marrow transplant OR BMT OR hemiepiphysiodesis OR spinal cord compression OR spinal surgery OR hip replacement OR hearing aid[MeSH Terms] OR hearing [MeSH Terms] OR respiration, artificial[MeSH Terms] OR tracheostomy OR orthopedic surgery OR anesthesia OR enzyme replacement therapy[MeSH Terms] OR enzyme replacement therapy OR ERT OR tonsillectomy OR adenoidectomy OR GALNS protein, human[Supplementary Concept] OR galsulfase[Supplementary Concept] OR vimizim OR naglazyme OR elosulfase alfa[Title/abstract] OR Treatment Outcome [MeSH Terms] OR clinical outcomes) | 1902 |  |
| Google scholar | 2017/07/07 (12:00) | Limited number of search terms per search query | mucopolysaccharidosis OR mucopolysaccharidosis IVA OR mucopolysaccharidosis VI OR Morquio syndrome OR Maroteaux-Lamy syndrome AND enzyme replacement therapy OR HSCT OR surgery OR treatment outcome OR clinical outcome | 14 | The total number of hits was 4820, but out of the first 12 pages (as advised by our mentor) only 13 were not appearing in the above-mentioned databases, specifically web of science |
| LDN World | 13/07/2017 | Years 2012–2017 | mucopolysaccharidosis | 20 | Articles found are all included in the molecular genetics and metabolism online journal |

**Appendix 1c. Results of the systematic literature review according to PRISMA**

Additional records identified through other sources*
(n=584)

Records identified through database searching
(n=4206)

## Identification

Records after duplicates removed
(n=3346)

## Screening

Records excluded
(n=2846)

Records screened
(n=3346)

Full-text articles excluded, with reasons (n=253)

Animal study: 8

Preclinical study: 9

No outcomes presented: 16

Single patient case study (only included for interventions where evidence was scarce): 59

No interventions evaluated: 80

Not in English: 18

More than 20 years old: 23

Unable to access: 3

Congress poster/abstract only or published elsewhere: 27

Review/editorial - not specific: 10

## Eligibility

Full-text articles assessed for eligibility
(n=500)

MPS VI Studies included in qualitative synthesis
(n=147)

MPS IVA Studies included in qualitative synthesis
(n=100)

## Included

*Includes relevant articles from Journal of Inherited Metabolic Disease, Molecular Genetics and Metabolism, Genetics in Medicine, Society for Inherited Metabolic Disorders (SIMD), Society for the Study of Inborn Errors of Metabolism (SSIEM), American College of Medical Genetics and Genomics (ACMG), Lysosomal Disease Network We’re Organizing Research on Lysosomal Diseases (LDN WORLD), SCOPUS and Cochrane.

**Appendix 2. The oxford centre for evidence-based medicine criteria**

**Table 1. Oxford Evidence Level Criteria**

|  | **Therapy / Prevention, Aetiology / Harm** | **Prognosis** | **Diagnosis** | **Differential diagnosis / symptom prevalence study** | **Economic and decision analyses** |
| --- | --- | --- | --- | --- | --- |
| 1a | SR (with homogeneity*) of RCTs | SR (with homogeneity*) of inception cohort studies; CDR”  validated in different populations | SR (with homogeneity*) of Level 1 diagnostic studies; CDR”  with 1b studies from different clinical centres | SR (with homogeneity*) of prospective cohort studies | SR (with homogeneity*) of Level 1 economic studies |
| 1b | Individual RCT (with narrow Confidence Interval”¡) | Individual inception cohort study with > 80% follow-up; CDR”  validated in a single population | Validating** cohort study with good” ” ”  reference standards; or CDR”  tested within one clinical centre | Prospective cohort study with good follow-up | Analysis based on clinically sensible costs or alternatives; systematic review(s) of the evidence; and including multi-way sensitivity analyses |
| 1c | All or none§ | All or none case-series | Absolute SpPins and SnNouts | All or none case-series | Absolute better-value or worse-value analyses ” ” ” “ |
| 2a | SR (with homogeneity*) of cohort studies | SR (with homogeneity*) of either retrospective cohort studies or untreated control groups in RCTs | SR (with homogeneity*) of Level >2 diagnostic studies | SR (with homogeneity*) of 2b and better studies | SR (with homogeneity*) of Level >2 economic studies |
| 2b | Individual cohort study (including low quality RCT; eg, <80% follow-up) | Retrospective cohort study or follow-up of untreated control patients in an RCT; Derivation of CDR”  or validated on split-sample§§§ only | Exploratory** cohort study with good” ” ”  reference standards; CDR”  after derivation, or validated only on split-sample§§§ or databases | Retrospective cohort study, or poor follow-up | Analysis based on clinically sensible costs or alternatives; limited review(s) of the evidence, or single studies; and including multi-way sensitivity analyses |
| 2c | “Outcomes” Research; Ecological studies | “Outcomes” Research |  | Ecological studies | Audit or outcomes research |
| 3a | SR (with homogeneity*) of case-control studies |  | SR (with homogeneity*) of 3b and better studies | SR (with homogeneity*) of 3b and better studies | SR (with homogeneity*) of 3b and better studies |
| 3b | Individual Case-Control Study |  | Non-consecutive study; or without consistently applied reference standards | Non-consecutive cohort study, or very limited population | Analysis based on limited alternatives or costs, poor quality estimates of data, but including sensitivity analyses incorporating clinically sensible variations. |
| 4 | Case-series (and poor quality cohort and case-control studies§§) | Case-series (and poor quality prognostic cohort studies***) | Case-control study, poor or non-independent reference standard | Case-series or superseded reference standards | Analysis with no sensitivity analysis |
| 5 | Expert opinion without explicit critical appraisal, or based on physiology, bench research or “first principles” | Expert opinion without explicit critical appraisal, or based on physiology, bench research or “first principles” | Expert opinion without explicit critical appraisal, or based on physiology, bench research or “first principles” | Expert opinion without explicit critical appraisal, or based on physiology, bench research or “first principles” | Expert opinion without explicit critical appraisal, or based on economic theory or “first principles” |

**Table 2. Oxford evidence grade criteria**

| **Grade** | **Rationale** |
| --- | --- |
| A | Consistent level 1 studies |
| B | Consistent level 2 or 3 studies ***or*** extrapolations from level 1 studies |
| C | Level 4 studies ***or*** extrapolations from level 2 or 3 studies |
| D | Level 5 evidence ***or*** troublingly inconsistent or inconclusive studies of any level |

**Appendix 3. AGREE II evaluation**

|  | **Review round 1** | | | | **Review round 2** | | | |
| --- | --- | --- | --- | --- | --- | --- | --- | --- |
| **Total number of Appraisers** | **Appraiser** | | |  | **Appraiser** | | |  |
| **3** | **1** | **2** | **3*** |  | **1** | **2** | **3*** |  |
|  |  |  |  |  |  |  |  |  |
| **Domain 1: scope and purpose** |  | | | |  | | | |
| **Q1.** The overall objective(s) of the guideline is (are) specifically described | 3 | 6 | 3 | 12 | 5 | 7 | 6 | 18 |
| **Q2.** The health question(s) covered by the guideline is (are) specifically described | 6 | 4 | 3 | 13 | 7 | 6 | 5 | 18 |
| **Q3.** The population (patients, public, etc.) to whom the guideline is meant to apply is specifically described | 5 | 6 | 6.5 | 17.5 | 5 | 7 | 7 | 19 |
|  | 14 | 16 | 12.5 |  | 17 | 20 | 18 |  |
|  |  |  |  | **42.5** |  |  |  | **55** |
|  | 62% | | | | 85% | | | |
|  |  |  |  |  |  |  |  |  |
| **Domain 2: stakeholder involvement** |  | | | |  | | | |
| **Q4.** The guideline development group includes individuals from all relevant professional groups | 5 | 7 | 3 | 15 | 5 | 7 | 3 | 15 |
| **Q5.** The views and preferences of the target population (patients, public, etc.) have been sought | 7 | 6 | 3 | 16 | 7 | 6 | 6 | 19 |
| **Q6.** The target users of the guideline are clearly defined | 5 | 5 | 5 | 15 | 7 | 7 | 7 | 21 |
|  | 17 | 18 | 11 |  | 19 | 20 | 16 |  |
|  |  |  |  | **46** |  |  |  | **55** |
|  | 69% | | | | 85% | | | |
|  |  |  |  |  |  |  |  |  |
| **Domain 3: Rigour of development** |  | | | |  | | | |
| **Q7.** Systematic methods were used to search for evidence | 4 | 5 | 2 | 11 | 7 | 7 | 5.5 | 19.5 |
| **Q8.** The criteria for selecting the evidence are clearly described | 6 | 4 | 2.5 | 12.5 | 7 | 5 | 4 | 16 |
| **Q9.** The strengths and limitations of the body of evidence are clearly described | 4 | 4 | 3 | 11 | 4 | 4 | 3 | 11 |
| **Q10.** The methods for formulating the recommendations are clearly described | 7 | 6 | 3.5 | 16.5 | 7 | 7 | 4 | 18 |
| **Q11.** The health benefits, side effects, and risks have been considered in formulating the recommendations | 6 | 4 | 5 | 15 | 6 | 4 | 5 | 15 |
| **Q12.** There is an explicit link between the recommendations and the supporting evidence | 7 | 6 | 6 | 19 | 7 | 6 | 6 | 19 |
| **Q13.** The guideline has been externally reviewed by experts prior to its publication | 6 | 2 | 7 | 15 | 6 | 2 | 7 | 15 |
| **Q14.** A procedure for updating the guideline is provided | 3 | 6 | 7 | 16 | 3 | 6 | 7 | 16 |
|  | 43 | 37 | 36 |  | 47 | 41 | 41.5 |  |
|  |  |  |  | **116** |  |  |  | **129.5** |
|  | 64% | | | | 73% | | | |
|  | | | | | | | | |
| **Domain 4: clarity of presentation** |  | | | |  | | | |
| **Q15.** The recommendations are specific and unambiguous | 7 | 6 | 7 | 20 | 7 | 6 | 7 | 20 |
| **Q16.** The different options for management of the condition or health issue are clearly presented | 7 | 4 | 7 | 18 | 7 | 4 | 7 | 18 |
| **Q17.** Key recommendations are easily identifiable | 1 | 6 | 3 | 10 | 4 | 6 | 3 | 13 |
|  | 15 | 16 | 17 |  | 18 | 16 | 17 |  |
|  |  |  |  | **48** |  |  |  | **51** |
|  | 72% | | | | 78% | | | |
|  |  |  |  |  |  |  |  |  |
| **Domain 5: applicability** |  | | | |  | | | |
| **Q18.** The guideline describes facilitators and barriers to its application | 7 | 3 | 1 | 11 | 7 | 3 | 2 | 12 |
| **Q19.** The guideline provides advice and/or tools on how the recommendations can be put into practice | 5 | 2 | 1 | 8 | 5 | 2 | 1 | 8 |
| **Q20.** The potential resource implications of applying the recommendations have been considered | 2 | 1 | 1 | 4 | 5 | 1 | 1.5 | 7.5 |
| **Q21.** The guideline presents monitoring and/or auditing criteria | 4 | 2 | 3 | 9 | 4 | 2 | 3 | 9 |
|  | 18 | 8 | 6 |  | 21 | 8 | 7.5 |  |
|  |  |  |  | **32** |  |  |  | **36.5** |
|  | 28% | | | | 34% | | | |
|  |  |  |  |  |  |  |  |  |
| **Domain 6: editorial independence** |  | | | |  | | | |
| **Q22.** The views of the funding body have not influenced the content of the guideline | 7 | 5 | 6 | 18 | 7 | 5 | 6 | 18 |
| **Q23.** Competing interests of guideline development group members have been recorded and addressed | 4 | 4 | 3 | 11 | 4 | 4 | 3 | 11 |
|  | 11 | 9 | 9 |  | 11 | 9 | 9 |  |
|  |  |  |  | **29** |  |  |  | **29** |
|  | 64% | | | | 64% | | | |
|  |  |  |  |  |  |  |  |  |
| **Overall guideline assessment** |  | | | |  | | | |
| **1.** Rate the overall quality of this guideline. *Scoring: 1 (lowest quality) – 7 (highest quality)* | 5 | 4 | 4 |  | 6 | 5 | 5 |  |
| **2.** I would recommend this guideline for use. *Scoring: "Yes", "Yes, with modifications", "No"* | Yes | Yes, with modifications | | | Yes | Yes, with modifications | | |

*Average scores calculated from two assessments carried out by three independent appraisers.
