# Supplementary material for: Recommendations for the management of MPS VI: systematic evidence- and consensus-based guidance
Source: Orphanet J Rare Dis. 2019 May 29;14:118. doi: 10.1186/s13023-019-1080-y (PMC6541999; doi:10.1186/s13023-019-1080-y)
Supplement: Supplementary file 2 — Oxford CEBM grading for MPS IVA: Tables detailing the evidence levels given to each reference supporting the MPS IVA guidance statements and the Evidence Grades applied to each guidance statement. Evidence levels were assessed using the Oxford Centre for Evidence-based Medicine and were based on the quality of evidence of each reference. For each guidance statement, an overall Evidence Grade was applied, based on the evidence levels of the supporting references. (DOCX 41 kb) [file 13023_2019_1080_MOESM2_ESM.docx]

# Additional File 2. Oxford Centre for Evidence-based Medicine grading for MPS IVA guidance statements

1. **DISEASE-MODIFYING INTERVENTIONS**

**1.1. ERT (elosulfase alfa) in patients with MPS IVA**

| Initiation of long-term ERT with elosulfase alfa at a dose of 2.0 mg/kg/week through intravenous infusion is recommended in all patients with MPS IVA as soon as possible after a confirmed diagnosis  ***Evidence Grade: B*** ***(Level 2 or 3 studies)*** | |
| --- | --- |
| **References** | **Evidence Level** |
| Hendriksz, C. J., R. Parini, M. D. AlSayed, J. Raiman, R. Giugliani, M. L. Solano Villarreal, J. J. Mitchell, B. K. Burton, N. Guelbert, F. Stewart, D. A. Hughes, K. I. Berger, P. Slasor, R. Matousek, E. Jurecki, A. J. Shaywitz and P. R. Harmatz (2016). "Long-term endurance and safety of elosulfase alfa enzyme replacement therapy in patients with Morquio A syndrome." Mol Genet Metab **119**(1-2): 131-143. | 3 |
| Hendriksz, C. J., B. Burton, T. R. Fleming, P. Harmatz, D. Hughes, S. A. Jones, S. P. Lin, E. Mengel, M. Scarpa, V. Valayannopoulos, R. Giugliani, S. Investigators, P. Slasor, D. Lounsbury and W. Dummer (2014). "Efficacy and safety of enzyme replacement therapy with BMN 110 (elosulfase alfa) for Morquio A syndrome (mucopolysaccharidosis IVA): a phase 3 randomised placebo-controlled study." J Inherit Metab Dis 37(6): 979-990. | 2 |
| Hendriksz, C., S. Santra, S. A. Jones, T. Geberhiwot, L. Jesaitis, B. Long, Y. Qi, S. M. Hawley and C. Decker (2018). "Safety, immunogenicity, and clinical outcomes in patients with Morquio A syndrome participating in 2 sequential open-label studies of elosulfase alfa enzyme replacement therapy (MOR-002/MOR-100), representing 5years of treatment." Mol Genet Metab **123**(4): 479-487. | 3 |
| Hendriksz, C. J., R. Parini, M. D. AlSayed, J. Raiman, R. Giugliani, J. J. Mitchell, B. K. Burton, N. Guelbert, F. J. Stewart, D. A. Hughes, R. Matousek, S. M. Hawley, C. Decker and P. R. Harmatz (2018). "Impact of long-term elosulfase alfa on activities of daily living in patients with Morquio A syndrome in an open-label, multi-center, phase 3 extension study." Mol Genet Metab **123**(2): 127-134. | 3 |
| Burton, B. K., K. I. Berger, G. D. Lewis, M. Tarnopolsky, M. Treadwell, J. J. Mitchell, N. Muschol, S. A. Jones, V. R. Sutton, G. M. Pastores, H. Lau, R. Sparkes, F. Genter, A. J. Shaywitz and P. Harmatz (2015). "Safety and physiological effects of two different doses of elosulfase alfa in patients with morquio a syndrome: A randomized, double-blind, pilot study." Am J Med Genet A **167A**(10): 2272-2281. | 2 |
| Hendriksz, C. J., R. Giugliani, P. Harmatz, E. Mengel, N. Guffon, V. Valayannopoulos, R. Parini, D. Hughes, G. M. Pastores, H. A. Lau, M. D. Al-Sayed, J. Raiman, S. Investigators, K. Yang, M. Mealiffe and C. Haller (2015). "Multi-domain impact of elosufase alfa in Morquio A syndrome in the pivotal phase III trial." Mol Genet Metab **114**(2): 178-185 | 2 |
| Hughes, D., R. Giugliani, N. Guffon, S. A. Jones, K. E. Mengel, R. Parini, R. Matousek, S. M. Hawley and A. Quartel (2017). "Clinical outcomes in a subpopulation of adults with Morquio A syndrome: results from a long-term extension study of elosulfase alfa." Orphanet J Rare Dis **12**(1): 98. | 3 |
| Jones, S. A., M. Bialer, R. Parini, K. Martin, H. Wang, K. Yang, A. J. Shaywitz and P. Harmatz (2015). "Safety and clinical activity of elosulfase alfa in pediatric patients with Morquio A syndrome (mucopolysaccharidosis IVA) less than 5 y." Pediatr Res **78**(6): 717-722. | 3 |
| NICE. (2015). "Elosulfase alfa for treating mucopolysaccharidosis type IVa. Available at: https://www.nice.org.uk/guidance/hst2. Accessed June 2018." | 1 |

**1.2. HSCT in patients with MPS IVA**

| Due to lack of evidence, HSCT cannot be recommended for patients with MPS IVA, and at this time, is considered an investigational procedure  ***Evidence Grade: D (Level 3/4 studies with inconsistent risk/benefit results)*** | |
| --- | --- |
| **References** | **Evidence Level** |
| Tomatsu, S., K. Sawamoto, C. J. Almeciga-Diaz, T. Shimada, M. B. Bober, Y. Chinen, H. Yabe, A. M. Montano, R. Giugliani, F. Kubaski, E. Yasuda, A. Rodriguez-Lopez, A. J. Espejo-Mojica, O. F. Sanchez, R. W. Mason, L. A. Barrera, W. G. Mackenzie and T. Orii (2015). "Impact of enzyme replacement therapy and hematopoietic stem cell transplantation in patients with Morquio A syndrome." Drug Des Devel Ther **9**: 1937-1953. | 4 |
| Sawamoto, K., Y. Suzuki, W. G. Mackenzie, M. C. Theroux, C. Pizarro, H. Yabe, K. E. Orii, R. W. Mason, T. Orii and S. Tomatsu (2016). "Current therapies for Morquio A syndrome and their clinical outcomes." Expert Opin Orphan Drugs 4(9): 941-951. | 4 |
| Yabe, H., A. Tanaka, Y. Chinen, S. Kato, K. Sawamoto, E. Yasuda, H. Shintaku, Y. Suzuki, T. Orii and S. Tomatsu (2016). "Hematopoietic stem cell transplantation for Morquio A syndrome." Mol Genet Metab **117**(2): 84-94. | 4 |
| Yasuda, E., Y. Suzuki, T. Shimada, K. Sawamoto, W. G. Mackenzie, M. C. Theroux, C. Pizarro, L. Xie, F. Miller, T. Rahman, H. H. Kecskemethy, K. Nagao, T. Morlet, T. H. Shaffer, Y. Chinen, H. Yabe, A. Tanaka, H. Shintaku, K. E. Orii, K. O. Orii, R. W. Mason, A. M. Montano, T. Fukao, T. Orii and S. Tomatsu (2016). "Activity of daily living for Morquio A syndrome." Mol Genet Metab **118**(2): 111-122 | 3 |
| Boelens, J. J., R. F. Wynn, A. O'Meara, P. Veys, Y. Bertrand, G. Souillet, J. E. Wraith, A. Fischer, M. Cavazzana-Calvo, K. W. Sykora, P. Sedlacek, A. Rovelli, C. S. Uiterwaal and N. Wulffraat (2007). "Outcomes of hematopoietic stem cell transplantation for Hurler's syndrome in Europe: a risk factor analysis for graft failure." Bone Marrow Transplant **40**(3): 225-233. | Extrapolation from MPS I – Level 4 |
| Coman, D. J., I. M. Hayes, V. Collins, M. Sahhar, J. E. Wraith and M. B. Delatycki (2011). "Enzyme replacement therapy and extended newborn screening for mucopolysaccharidoses: opinions of treating physicians." JIMD Rep **1**: 9-15. | Extrapolation from MPS I, II, VI – Level 5 |
| Boelens, J. J., M. Aldenhoven, D. Purtill, A. Ruggeri, T. Defor, R. Wynn, E. Wraith, M. Cavazzana-Calvo, A. Rovelli, A. Fischer, J. Tolar, V. K. Prasad, M. Escolar, E. Gluckman, A. O'Meara, P. J. Orchard, P. Veys, M. Eapen, J. Kurtzberg, V. Rocha, Eurocord, B. Inborn Errors Working Party of European, g. Marrow Transplant, B. Duke University, P. Marrow Transplantation, B. Centre for International and R. Marrow (2013). "Outcomes of transplantation using various hematopoietic cell sources in children with Hurler syndrome after myeloablative conditioning." Blood 121(19): 3981-3987. | Extrapolation from MPS I - Level 4 |
| Patel, P., Y. Suzuki, A. Tanaka, H. Yabe, S. Kato, T. Shimada, R. W. Mason, K. E. Orii, T. Fukao, T. Orii and S. Tomatsu (2014). "Impact of Enzyme Replacement Therapy and Hematopoietic Stem Cell Therapy on Growth in Patients with Hunter Syndrome." Mol Genet Metab Rep **1**: 184-196. | Extrapolation from MPS II - Level 3 |
| Tomatsu, S., A. M. Montano, H. Oikawa, M. Smith, L. Barrera, Y. Chinen, M. M. Thacker, W. G. Mackenzie, Y. Suzuki and T. Orii (2011). "Mucopolysaccharidosis type IVA (Morquio A disease): clinical review and current treatment." Curr Pharm Biotechnol **12**(6): 931-945. | N/A |
| Algahim, M. F. and G. H. Almassi (2013). "Current and emerging management options for patients with Morquio A syndrome." Ther Clin Risk Manag **9**: 45-53. | N/A |
| Hendriksz, C. J., K. I. Berger, R. Giugliani, P. Harmatz, C. Kampmann, W. G. Mackenzie, J. Raiman, M. S. Villarreal and R. Savarirayan (2015). "International guidelines for the management and treatment of Morquio A syndrome." Am J Med Genet A **167A**(1): 11-25. | N/A |

1. **INTERVENTIONS TO SUPPORT RESPIRATORY AND SLEEP DISORDERS**

**2.1. Respiratory interventions and sleep disorders in patients with MPS IVA**

| Continuous positive airway pressure (CPAP) therapy is recommended for patients with MPS IVA who display the presence of obstructive sleep apnoea (OSA) that persists after tonsillectomy and/or adenoidectomy  ***Evidence Grade: D (Limited published evidence)*** | |
| --- | --- |
| **References** | **Evidence Level** |
| John, A., S. Fagondes, I. Schwartz, A. C. Azevedo, P. Barrios, P. Dalcin, S. Menna-Barreto and R. Giugliani (2011). "Sleep abnormalities in untreated patients with mucopolysaccharidosis type VI." Am J Med Genet A **155A**(7): 1546-1551. | Extrapolation from Level 3 prospective cohort study |
| Berger, K. I., S. C. Fagondes, R. Giugliani, K. A. Hardy, K. S. Lee, C. McArdle, M. Scarpa, M. J. Tobin, S. A. Ward and D. M. Rapoport (2013). "Respiratory and sleep disorders in mucopolysaccharidosis." J Inherit Metab Dis **36**(2): 201-210. | N/A |
| Leighton, S. E., B. Papsin, A. Vellodi, R. Dinwiddie and R. Lane (2001). "Disordered breathing during sleep in patients with mucopolysaccharidoses." Int J Pediatr Otorhinolaryngol **58**(2): 127-138. | 3 |
| Walker, P. P., E. Rose and J. G. Williams (2003). "Upper airways abnormalities and tracheal problems in Morquio's disease." Thorax **58**(5): 458-459. | 4 |
| Piper, A. J. and B. J. Yee (2014). "Hypoventilation syndromes." Compr Physiol **4**(4): 1639-1676. | N/A |

| Non-invasive positive pressure ventilation (NIPPV) therapy is recommended for patients with MPS IVA who display nocturnal hypoventilation and are unresponsive to CPAP, or display daytime hypoventilation with increased PaCO_2_ and/or serum HCO_3_ levels  ***Evidence Grade: D (Level 5 expert clinical opinion)*** | |
| --- | --- |
| **References** | **Evidence Level** |
| Piper, A. J. and B. J. Yee (2014). "Hypoventilation syndromes." Compr Physiol **4**(4): 1639-1676. | N/A |

| Oxygen supplementation during sleep is recommended for patients with MPS IVA who exhibit sleep apnoea with nocturnal hypoxemia, and who do not tolerate CPAP or NIPPV masks  ***Evidence Grade: D (Level 5 expert clinical opinion)*** |
| --- |

| Patients with MPS IVA should be monitored for development of hypercapnia after starting oxygen therapy using measurement of PaCO_2_ and/or serum HCO_3_  ***Evidence Grade: D (Level 5 expert clinical opinion)*** | |
| --- | --- |
| **References** | **Evidence Level** |
| Berger, K. I., S. C. Fagondes, R. Giugliani, K. A. Hardy, K. S. Lee, C. McArdle, M. Scarpa, M. J. Tobin, S. A. Ward and D. M. Rapoport (2013). "Respiratory and sleep disorders in mucopolysaccharidosis." J Inherit Metab Dis **36**(2): 201-210. | N/A |
| Piper, A. J. and B. J. Yee (2014). "Hypoventilation syndromes." Compr Physiol **4**(4): 1639-1676. | N/A |

1. **ANAESTHESIA AND SURGICAL INTERVENTIONS**

**3.1. Anaesthesia in patients with MPS IVA**

| Pre-, intra- and post-operative care (until extubation is complete) for all procedures requiring general anesthesia, conscious or deep sedation, should be supervised by an anesthetist with experience in treating patients with MPS and/or complex airway management. In addition, the anesthetist should have access to Intensive Care support and be surrounded by an experienced team capable of performing emergency tracheotomy if required  ***Evidence Grade: C (Consistent Level 4 studies)*** | |
| --- | --- |
| **References** | **Evidence Level** |
| Moores, C., J. G. Rogers, I. M. McKenzie and T. C. Brown (1996). "Anaesthesia for children with mucopolysaccharidoses." Anaesth Intensive Care **24**(4): 459-463. | 4 |
| Walker, R., K. G. Belani, E. A. Braunlin, I. A. Bruce, H. Hack, P. R. Harmatz, S. Jones, R. Rowe, G. A. Solanki and B. Valdemarsson (2013). "Anaesthesia and airway management in mucopolysaccharidosis." J Inherit Metab Dis **36**(2): 211-219. | N/A |
| Walker, R. W., M. Darowski, P. Morris and J. E. Wraith (1994). "Anaesthesia and mucopolysaccharidoses. A review of airway problems in children." Anaesthesia **49**(12): 1078-1084. | 3 |
| Tobias, J. D. (1999). "Anesthetic care for the child with Morquio syndrome: general versus regional anesthesia." J Clin Anesth **11**(3): 242-246. | 4 |
| Frawley, G., D. Fuenzalida, S. Donath, J. Yaplito-Lee and H. Peters (2012). "A retrospective audit of anesthetic techniques and complications in children with mucopolysaccharidoses." Paediatr Anaesth **22**(8): 737-744. | 3 |
| Walker, R. W., D. L. Allen and M. R. Rothera (1997). "A fibreoptic intubation technique for children with mucopolysaccharidoses using the laryngeal mask airway." Paediatr Anaesth **7**(5): 421-426. | 4 |
| Walker, P. P., E. Rose and J. G. Williams (2003). "Upper airways abnormalities and tracheal problems in Morquio's disease." Thorax **58**(5): 458-459. | 4 |
| Walker, R. W., V. Colovic, D. N. Robinson and O. R. Dearlove (2003). "Postobstructive pulmonary oedema during anaesthesia in children with mucopolysaccharidoses." Paediatr Anaesth **13**(5): 441-447. | 4 |
| Geetha, L., M. Radhakrishnan, B. S. Raghavendra, G. S. Rao and B. Indira Devi (2010). "Anesthetic management for foramen magnum decompression in a patient with Morquio syndrome: a case report." J Anesth **24**(4): 594-597. | 4 |
| Theroux, M. C., T. Nerker, C. Ditro and W. G. Mackenzie (2012). "Anesthetic care and perioperative complications of children with Morquio syndrome." Paediatr Anaesth **22**(9): 901-907. | 3 |
| Herrick, I. A. and E. J. Rhine (1988). "The mucopolysaccharidoses and anaesthesia: a report of clinical experience." Can J Anaesth **35**(1): 67-73. | 4 |
| Drummond, J. C., E. J. Krane, S. Tomatsu, M. C. Theroux and R. R. Lee (2015). "Paraplegia after epidural-general anesthesia in a Morquio patient with moderate thoracic spinal stenosis." Can J Anaesth **62**(1): 45-49. | 4 |
| Charrow, J., T. D. Alden, C. A. Breathnach, G. P. Frawley, C. J. Hendriksz, B. Link, W. G. Mackenzie, R. Manara, A. C. Offiah, M. L. Solano and M. Theroux (2015). "Diagnostic evaluation, monitoring, and perioperative management of spinal cord compression in patients with Morquio syndrome." Mol Genet Metab **114**(1): 11-18. | 5 |

| A full assessment of the risks and benefits should take place with the patient and family prior to any procedure. All pre-operative information should be made available to allow decision making  ***Evidence Grade: C (Consistent Level 4 studies)*** | |
| --- | --- |
| **References** | **Evidence Level** |
| Moores, C., J. G. Rogers, I. M. McKenzie and T. C. Brown (1996). "Anaesthesia for children with mucopolysaccharidoses." Anaesth Intensive Care **24**(4): 459-463. | 4 |
| Walker, R., K. G. Belani, E. A. Braunlin, I. A. Bruce, H. Hack, P. R. Harmatz, S. Jones, R. Rowe, G. A. Solanki and B. Valdemarsson (2013). "Anaesthesia and airway management in mucopolysaccharidosis." J Inherit Metab Dis **36**(2): 211-219. | N/A |
| Walker, R. W., M. Darowski, P. Morris and J. E. Wraith (1994). "Anaesthesia and mucopolysaccharidoses. A review of airway problems in children." Anaesthesia **49**(12): 1078-1084. | 3 |
| Tobias, J. D. (1999). "Anesthetic care for the child with Morquio syndrome: general versus regional anesthesia." J Clin Anesth **11**(3): 242-246. | 4 |
| Frawley, G., D. Fuenzalida, S. Donath, J. Yaplito-Lee and H. Peters (2012). "A retrospective audit of anesthetic techniques and complications in children with mucopolysaccharidoses." Paediatr Anaesth **22**(8): 737-744. | 3 |
| Bartz, H. J., L. Wiesner and F. Wappler (1999). "Anaesthetic management of patients with mucopolysaccharidosis IV presenting for major orthopaedic surgery." Acta Anaesthesiol Scand **43**(6): 679-683. | 4 |
| Walker, R. W., V. Colovic, D. N. Robinson and O. R. Dearlove (2003). "Postobstructive pulmonary oedema during anaesthesia in children with mucopolysaccharidoses." Paediatr Anaesth **13**(5): 441-447. | 4 |
| Theroux, M. C., T. Nerker, C. Ditro and W. G. Mackenzie (2012). "Anesthetic care and perioperative complications of children with Morquio syndrome." Paediatr Anaesth **22**(9): 901-907. | 3 |

| ENT respiratory, cardiac, and radiological assessment should be performed prior to any procedure requiring anesthesia  ***Evidence Grade: C (Consistent Level 4 studies)*** | |
| --- | --- |
| **References** | **Evidence Level** |
| Moores, C., J. G. Rogers, I. M. McKenzie and T. C. Brown (1996). "Anaesthesia for children with mucopolysaccharidoses." Anaesth Intensive Care **24**(4): 459-463. | 4 |
| Walker, R., K. G. Belani, E. A. Braunlin, I. A. Bruce, H. Hack, P. R. Harmatz, S. Jones, R. Rowe, G. A. Solanki and B. Valdemarsson (2013). "Anaesthesia and airway management in mucopolysaccharidosis." J Inherit Metab Dis **36**(2): 211-219. | N/A |
| Walker, R. W., M. Darowski, P. Morris and J. E. Wraith (1994). "Anaesthesia and mucopolysaccharidoses. A review of airway problems in children." Anaesthesia **49**(12): 1078-1084. | 3 |
| Tobias, J. D. (1999). "Anesthetic care for the child with Morquio syndrome: general versus regional anesthesia." J Clin Anesth **11**(3): 242-246. | 4 |
| Frawley, G., D. Fuenzalida, S. Donath, J. Yaplito-Lee and H. Peters (2012). "A retrospective audit of anesthetic techniques and complications in children with mucopolysaccharidoses." Paediatr Anaesth **22**(8): 737-744. | 3 |
| Walker, R. W., D. L. Allen and M. R. Rothera (1997). "A fibreoptic intubation technique for children with mucopolysaccharidoses using the laryngeal mask airway." Paediatr Anaesth **7**(5): 421-426. | 4 |
| Bartz, H. J., L. Wiesner and F. Wappler (1999). "Anaesthetic management of patients with mucopolysaccharidosis IV presenting for major orthopaedic surgery." Acta Anaesthesiol Scand **43**(6): 679-683. | 4 |
| Walker, P. P., E. Rose and J. G. Williams (2003). "Upper airways abnormalities and tracheal problems in Morquio's disease." Thorax **58**(5): 458-459. | 4 |
| Walker, R. W., V. Colovic, D. N. Robinson and O. R. Dearlove (2003). "Postobstructive pulmonary oedema during anaesthesia in children with mucopolysaccharidoses." Paediatr Anaesth **13**(5): 441-447. | 4 |
| Geetha, L., M. Radhakrishnan, B. S. Raghavendra, G. S. Rao and B. Indira Devi (2010). "Anesthetic management for foramen magnum decompression in a patient with Morquio syndrome: a case report." J Anesth **24**(4): 594-597. | 4 |
| Theroux, M. C., T. Nerker, C. Ditro and W. G. Mackenzie (2012). "Anesthetic care and perioperative complications of children with Morquio syndrome." Paediatr Anaesth **22**(9): 901-907. | 3 |
| Herrick, I. A. and E. J. Rhine (1988). "The mucopolysaccharidoses and anaesthesia: a report of clinical experience." Can J Anaesth **35**(1): 67-73. | 4 |
| Drummond, J. C., E. J. Krane, S. Tomatsu, M. C. Theroux and R. R. Lee (2015). "Paraplegia after epidural-general anesthesia in a Morquio patient with moderate thoracic spinal stenosis." Can J Anaesth **62**(1): 45-49. | 4 |
| Charrow, J., T. D. Alden, C. A. Breathnach, G. P. Frawley, C. J. Hendriksz, B. Link, W. G. Mackenzie, R. Manara, A. C. Offiah, M. L. Solano and M. Theroux (2015). "Diagnostic evaluation, monitoring, and perioperative management of spinal cord compression in patients with Morquio syndrome." Mol Genet Metab **114**(1): 11-18. | 5 |

| It is critical to maintain a neutral neck position during all surgeries, and during intubation and extubation to avoid paralysis. Strongly recommend the use of techniques that allow maintenance of the neutral neck position, including use of laryngeal mask airway (LMA) for shorter procedures, or intubation with a video laryngoscope or fiberoptic intubation  ***Evidence Grade: C (Consistent Level 4 studies)*** | |
| --- | --- |
| **References** | **Evidence Level** |
| Moores, C., J. G. Rogers, I. M. McKenzie and T. C. Brown (1996). "Anaesthesia for children with mucopolysaccharidoses." Anaesth Intensive Care **24**(4): 459-463. | 4 |
| Walker, R., K. G. Belani, E. A. Braunlin, I. A. Bruce, H. Hack, P. R. Harmatz, S. Jones, R. Rowe, G. A. Solanki and B. Valdemarsson (2013). "Anaesthesia and airway management in mucopolysaccharidosis." J Inherit Metab Dis **36**(2): 211-219. | N/A |
| Walker, R. W., M. Darowski, P. Morris and J. E. Wraith (1994). "Anaesthesia and mucopolysaccharidoses. A review of airway problems in children." Anaesthesia **49**(12): 1078-1084. | 3 |
| Tobias, J. D. (1999). "Anesthetic care for the child with Morquio syndrome: general versus regional anesthesia." J Clin Anesth **11**(3): 242-246. | 4 |
| Frawley, G., D. Fuenzalida, S. Donath, J. Yaplito-Lee and H. Peters (2012). "A retrospective audit of anesthetic techniques and complications in children with mucopolysaccharidoses." Paediatr Anaesth **22**(8): 737-744. | 3 |
| Walker, R. W., D. L. Allen and M. R. Rothera (1997). "A fibreoptic intubation technique for children with mucopolysaccharidoses using the laryngeal mask airway." Paediatr Anaesth **7**(5): 421-426. | 4 |
| Bartz, H. J., L. Wiesner and F. Wappler (1999). "Anaesthetic management of patients with mucopolysaccharidosis IV presenting for major orthopaedic surgery." Acta Anaesthesiol Scand **43**(6): 679-683. | 4 |
| Walker, R. W., V. Colovic, D. N. Robinson and O. R. Dearlove (2003). "Postobstructive pulmonary oedema during anaesthesia in children with mucopolysaccharidoses." Paediatr Anaesth **13**(5): 441-447. | 4 |
| Geetha, L., M. Radhakrishnan, B. S. Raghavendra, G. S. Rao and B. Indira Devi (2010). "Anesthetic management for foramen magnum decompression in a patient with Morquio syndrome: a case report." J Anesth **24**(4): 594-597. | 4 |
| Theroux, M. C., T. Nerker, C. Ditro and W. G. Mackenzie (2012). "Anesthetic care and perioperative complications of children with Morquio syndrome." Paediatr Anaesth **22**(9): 901-907. | 3 |
| Drummond, J. C., E. J. Krane, S. Tomatsu, M. C. Theroux and R. R. Lee (2015). "Paraplegia after epidural-general anesthesia in a Morquio patient with moderate thoracic spinal stenosis." Can J Anaesth **62**(1): 45-49. | 4 |
| Charrow, J., T. D. Alden, C. A. Breathnach, G. P. Frawley, C. J. Hendriksz, B. Link, W. G. Mackenzie, R. Manara, A. C. Offiah, M. L. Solano and M. Theroux (2015). "Diagnostic evaluation, monitoring, and perioperative management of spinal cord compression in patients with Morquio syndrome." Mol Genet Metab **114**(1): 11-18. | 5 |

| Pre-operative and intra-operative measures to avoid hypotension should be adopted during all surgical procedures in patients with MPS IVA to maintain spinal cord perfusion and therefore protect spinal cord function  ***Evidence Grade: D (Limited published evidence)*** | |
| --- | --- |
| **References** | **Evidence Level** |
| Bartz, H. J., L. Wiesner and F. Wappler (1999). "Anaesthetic management of patients with mucopolysaccharidosis IV presenting for major orthopaedic surgery." Acta Anaesthesiol Scand **43**(6): 679-683. | 4 |
| Drummond, J. C., E. J. Krane, S. Tomatsu, M. C. Theroux and R. R. Lee (2015). "Paraplegia after epidural-general anesthesia in a Morquio patient with moderate thoracic spinal stenosis." Can J Anaesth **62**(1): 45-49. | 4 |
| Charrow, J., T. D. Alden, C. A. Breathnach, G. P. Frawley, C. J. Hendriksz, B. Link, W. G. Mackenzie, R. Manara, A. C. Offiah, M. L. Solano and M. Theroux (2015). "Diagnostic evaluation, monitoring, and perioperative management of spinal cord compression in patients with Morquio syndrome." Mol Genet Metab **114**(1): 11-18. | 5 |

| Intra-operative neurophysiological monitoring (including SSEP, electromyography [EMG] and motor evoked potentials [MEP]) is strongly recommended during all spinal surgeries and other potentially lengthy or complicated procedures, including those that require manipulation of the head and neck  ***Evidence Grade: D (Limited published evidence)*** | |
| --- | --- |
| **References** | **Evidence Level** |
| Walker, R., K. G. Belani, E. A. Braunlin, I. A. Bruce, H. Hack, P. R. Harmatz, S. Jones, R. Rowe, G. A. Solanki and B. Valdemarsson (2013). "Anaesthesia and airway management in mucopolysaccharidosis." J Inherit Metab Dis **36**(2): 211-219. | N/A |
| Charrow, J., T. D. Alden, C. A. Breathnach, G. P. Frawley, C. J. Hendriksz, B. Link, W. G. Mackenzie, R. Manara, A. C. Offiah, M. L. Solano and M. Theroux (2015). "Diagnostic evaluation, monitoring, and perioperative management of spinal cord compression in patients with Morquio syndrome." Mol Genet Metab **114**(1): 11-18. | 5 |

| For other surgeries and procedures, neurophysiologic monitoring should be considered based on pre-existing risk for spinal cord compression and instability, need for spine manipulation, possibility of hemodynamic changes and blood loss, or extended length of time  ***Evidence Grade: D (Limited published evidence)*** | |
| --- | --- |
| **References** | **Evidence Level** |
| Walker, R., K. G. Belani, E. A. Braunlin, I. A. Bruce, H. Hack, P. R. Harmatz, S. Jones, R. Rowe, G. A. Solanki and B. Valdemarsson (2013). "Anaesthesia and airway management in mucopolysaccharidosis." J Inherit Metab Dis **36**(2): 211-219. | N/A |
| Drummond, J. C., E. J. Krane, S. Tomatsu, M. C. Theroux and R. R. Lee (2015). "Paraplegia after epidural-general anesthesia in a Morquio patient with moderate thoracic spinal stenosis." Can J Anaesth **62**(1): 45-49. | 4 |
| Charrow, J., T. D. Alden, C. A. Breathnach, G. P. Frawley, C. J. Hendriksz, B. Link, W. G. Mackenzie, R. Manara, A. C. Offiah, M. L. Solano and M. Theroux (2015). "Diagnostic evaluation, monitoring, and perioperative management of spinal cord compression in patients with Morquio syndrome." Mol Genet Metab **114**(1): 11-18. | 5 |

| Intrathecal and epidural techniques are high-risk in patients with MPS IVA and should be avoided wherever possible  ***Evidence Grade: D (Limited published evidence)*** | |
| --- | --- |
| **References** | **Evidence Level** |
| Drummond, J. C., E. J. Krane, S. Tomatsu, M. C. Theroux and R. R. Lee (2015). "Paraplegia after epidural-general anesthesia in a Morquio patient with moderate thoracic spinal stenosis." Can J Anaesth **62**(1): 45-49. | 4 |
| Charrow, J., T. D. Alden, C. A. Breathnach, G. P. Frawley, C. J. Hendriksz, B. Link, W. G. Mackenzie, R. Manara, A. C. Offiah, M. L. Solano and M. Theroux (2015). "Diagnostic evaluation, monitoring, and perioperative management of spinal cord compression in patients with Morquio syndrome." Mol Genet Metab **114**(1): 11-18. | 5 |

**3.2. Limb surgeries in patients with MPS IVA**

| Hip reconstruction can be considered in paediatric patients with MPS IVA who exhibit hip pain, reduced walking and endurance related to hip disease, as well as abnormal radiographic findings  ***Evidence Grade: D (Limited published evidence)*** | |
| --- | --- |
| **References** | **Evidence Level** |
| Dhawale, A. A., M. M. Thacker, M. V. Belthur, K. Rogers, M. B. Bober and W. G. Mackenzie (2012). "The lower extremity in Morquio syndrome." J Pediatr Orthop **32**(5): 534-540. | 3 |
| White, K. K., A. Jester, C. E. Bache, P. R. Harmatz, R. Shediac, M. M. Thacker and W. G. Mackenzie (2014). "Orthopedic management of the extremities in patients with Morquio A syndrome." J Child Orthop **8**(4): 295-304. | N/A |
| Montano, A. M., S. Tomatsu, G. S. Gottesman, M. Smith and T. Orii (2007). "International Morquio A Registry: clinical manifestation and natural course of Morquio A disease." J Inherit Metab Dis **30**(2): 165-174. | 4 |
| White, K. K. (2011). "Orthopaedic aspects of mucopolysaccharidoses." Rheumatology (Oxford) **50 Suppl 5**: v26-33. | N/A |
| White, K. K. and T. Sousa (2013). "Mucopolysaccharide disorders in orthopaedic surgery." J Am Acad Orthop Surg **21**(1): 12-22. | N/A |

| Hip replacement can be considered in adult patients with MPS IVA who exhibit hip pain, reduced walking and endurance related to hip disease, as well as abnormal radiographic findings  ***Evidence Grade: D (Limited published evidence)*** | |
| --- | --- |
| **References** | **Evidence Level** |
| Dhawale, A. A., M. M. Thacker, M. V. Belthur, K. Rogers, M. B. Bober and W. G. Mackenzie (2012). "The lower extremity in Morquio syndrome." J Pediatr Orthop **32**(5): 534-540. | 3 |
| White, K. K., A. Jester, C. E. Bache, P. R. Harmatz, R. Shediac, M. M. Thacker and W. G. Mackenzie (2014). "Orthopedic management of the extremities in patients with Morquio A syndrome." J Child Orthop **8**(4): 295-304. | N/A |
| Montano, A. M., S. Tomatsu, G. S. Gottesman, M. Smith and T. Orii (2007). "International Morquio A Registry: clinical manifestation and natural course of Morquio A disease." J Inherit Metab Dis **30**(2): 165-174. | 4 |

| Growth modulation surgery is recommended for all patients with MPS IVA who have evidence of genu valgum and should be performed as early as possible during the period of growth  ***Evidence Grade: D (Limited published evidence)*** | |
| --- | --- |
| **References** | **Evidence Level** |
| White, K. K., A. Jester, C. E. Bache, P. R. Harmatz, R. Shediac, M. M. Thacker and W. G. Mackenzie (2014). "Orthopedic management of the extremities in patients with Morquio A syndrome." J Child Orthop **8**(4): 295-304. | N/A |
| Montano, A. M., S. Tomatsu, G. S. Gottesman, M. Smith and T. Orii (2007). "International Morquio A Registry: clinical manifestation and natural course of Morquio A disease." J Inherit Metab Dis **30**(2): 165-174. | 4 |
| White, K. K. (2011). "Orthopaedic aspects of mucopolysaccharidoses." Rheumatology (Oxford) **50 Suppl 5**: v26-33. | N/A |
| White, K. K. and T. Sousa (2013). "Mucopolysaccharide disorders in orthopaedic surgery." J Am Acad Orthop Surg **21**(1): 12-22. | N/A |
| Cooper, G. A., T. Southorn, D. M. Eastwood and C. E. Bache (2016). "Lower Extremity Deformity Management in MPS IVA, Morquio-Brailsford Syndrome: Preliminary Report of Hemiepiphysiodesis Correction of Genu Valgum." J Pediatr Orthop **36**(4): 376-381. | 3 |
| Williams, N., D. Challoumas and D. M. Eastwood (2017). "Does orthopaedic surgery improve quality of life and function in patients with mucopolysaccharidoses?" J Child Orthop **11**(4): 289-297. | 4 (systematic review of Level 4/5 evidence) |
| de Waal Malefijt, M. C., A. van Kampen and J. J. van Gemund (2000). "Total knee arthroplasty in patients with inherited dwarfism--a report of five knee replacements in two patients with Morquio's disease type A and one with spondylo-epiphyseal dysplasia." Arch Orthop Trauma Surg **120**(3-4): 179-182 | 4 |

**3.3. Spinal surgeries in patients with MPS IVA**

| Decompression of the spinal cord is recommended in patients with MPS IVA who have evidence of spinal cord compression based on clinical and radiographic findings  ***Evidence Grade: C (Level 3/4 studies)*** | |
| --- | --- |
| **References** | **Evidence Level** |
| Ransford, A. O., H. A. Crockard, J. M. Stevens and S. Modaghegh (1996). "Occipito-atlanto-axial fusion in Morquio-Brailsford syndrome. A ten-year experience." J Bone Joint Surg Br **78**(2): 307-313. | 3 |
| Blaw, M. E. and L. O. Langer (1969). "Spinal cord compression in Morquio-Brailsford's disease." J Pediatr **74**(4): 593-600. | 4 |
| White, K. K. (2011). "Orthopaedic aspects of mucopolysaccharidoses." Rheumatology (Oxford) **50 Suppl 5**: v26-33. | N/A |
| Dalvie, S., J. Skinner, A. Vellodi and M. H. Noorden (2001). "Mobile thoracolumbar gibbus in Morquio type A: the cause of paraparesis and its management." J Pediatr Orthop B **10**(4): 328-330. | 4 |
| Dede, O., M. M. Thacker, K. J. Rogers, M. Oto, M. V. Belthur, W. Baratela and W. G. Mackenzie (2013). "Upper cervical fusion in children with Morquio syndrome: intermediate to long-term results." J Bone Joint Surg Am **95**(13): 1228-1234. | 3 |
| Garrido, E., F. Tome-Bermejo and C. I. Adams (2014). "Combined spinal arthrodesis with instrumentation for the management of progressive thoracolumbar kyphosis in children with mucopolysaccharidosis." Eur Spine J **23**(12): 2751-2757. | 4 |

| Spinal stabilisation of the craniocervical junction with either cervical fusion or occipital-cervical fusion is recommended in patients with MPS IVA who have evidence of significant instability  ***Evidence Grade: D (Limited published evidence)*** | |
| --- | --- |
| **References** | **Evidence Level** |
| Dede, O., M. M. Thacker, K. J. Rogers, M. Oto, M. V. Belthur, W. Baratela and W. G. Mackenzie (2013). "Upper cervical fusion in children with Morquio syndrome: intermediate to long-term results." J Bone Joint Surg Am **95**(13): 1228-1234. | 3 |

| Correction of thoracolumbar kyphoscoliosis is recommended in patients with MPS IVA who present with progressive radiographic deformity, intractable pain and neurological deterioration  ***Evidence Grade: C (Level 3/4 studies)*** | |
| --- | --- |
| **References** | **Evidence Level** |
| Ransford, A. O., H. A. Crockard, J. M. Stevens and S. Modaghegh (1996). "Occipito-atlanto-axial fusion in Morquio-Brailsford syndrome. A ten-year experience." J Bone Joint Surg Br **78**(2): 307-313. | 3 |
| White, K. K. (2011). "Orthopaedic aspects of mucopolysaccharidoses." Rheumatology (Oxford) **50 Suppl 5**: v26-33. | N/A |
| Dalvie, S., J. Skinner, A. Vellodi and M. H. Noorden (2001). "Mobile thoracolumbar gibbus in Morquio type A: the cause of paraparesis and its management." J Pediatr Orthop B **10**(4): 328-330. | 4 |
| Dede, O., M. M. Thacker, K. J. Rogers, M. Oto, M. V. Belthur, W. Baratela and W. G. Mackenzie (2013). "Upper cervical fusion in children with Morquio syndrome: intermediate to long-term results." J Bone Joint Surg Am **95**(13): 1228-1234. | 3 |
| Dalvie, S. S., M. H. Noordeen and A. Vellodi (2001). "Anterior instrumented fusion for thoracolumbar kyphosis in mucopolysaccharidosis." Spine (Phila Pa 1976) **26**(23): E539-541. | 4 |
| Garrido, E., F. Tome-Bermejo and C. I. Adams (2014). "Combined spinal arthrodesis with instrumentation for the management of progressive thoracolumbar kyphosis in children with mucopolysaccharidosis." Eur Spine J **23**(12): 2751-2757. | 4 |

**3.4. Ophthalmic surgery in patients with MPS IVA**

| While significant corneal clouding is rare in patients with MPS IVA, corneal transplantation can be considered for patients with significant visual loss attributed to corneal opacification  ***Evidence Grade: D (Limited published evidence)*** | |
| --- | --- |
| **References** | **Evidence Level** |
| Ashworth, J. L., S. Biswas, E. Wraith and I. C. Lloyd (2006). "Mucopolysaccharidoses and the eye." Surv Ophthalmol **51**(1): 1-17. | N/A |
| Bothun, E. D., A. Decanini, C. G. Summers, P. J. Orchard and J. Tolar (2011). "Outcome of penetrating keratoplasty for mucopolysaccharidoses." Arch Ophthalmol **129**(2): 138-144. | 4 |
| Ohden, K. L., S. Pitz, J. Ashworth, A. Magalhaes, D. R. Marinho, P. Lindahl, K. Tear Fahnehjelm and C. G. Summers (2017). "Outcomes of keratoplasty in the mucopolysaccharidoses: an international perspective." Br J Ophthalmol **101**(7): 909-912. | 3 |
| Keane, M., D. Coster, M. Ziaei and K. Williams (2014). "Deep anterior lamellar keratoplasty versus penetrating keratoplasty for treating keratoconus." Cochrane Database Syst Rev(7): CD009700. | Extrapolation from Level 1 systematic review (general population) |
| Fenzl, C. R., K. Teramoto and M. Moshirfar (2015). "Ocular manifestations and management recommendations of lysosomal storage disorders I: mucopolysaccharidoses." Clin Ophthalmol **9**: 1633-1644. | N/A |

**3.5. Cardio-thoracic surgery in patients with MPS IVA**

| Cardiac (aortic, mitral) valve replacement should be considered in patients with MPS IVA who display symptomatic and severe valve stenosis or regurgitation  ***Evidence Grade: C (Level 4 studies)*** | |
| --- | --- |
| **References** | **Evidence Level** |
| Braunlin, E. A., P. R. Harmatz, M. Scarpa, B. Furlanetto, C. Kampmann, J. P. Loehr, K. P. Ponder, W. C. Roberts, H. M. Rosenfeld and R. Giugliani (2011). "Cardiac disease in patients with mucopolysaccharidosis: presentation, diagnosis and management." J Inherit Metab Dis **34**(6): 1183-1197. | N/A - Reports succesful replacements in other MPS types (exapolation) |
| Barry, M. O., M. A. Beardslee and A. C. Braverman (2006). "Morquio's syndrome: severe aortic regurgitation and late pulmonary autograft failure." J Heart Valve Dis **15**(6): 839-842. | 4 |
| Nicolini F, Corradi D, Bosio S, Gherli T (2008) “Aortic valve replacement in a patient with morquio syndrome.” Heart Surg Forum 11:E96–E98 | 4 |
| Pagel PS, Almassi GH (2009) “Perioperative implications of Morquio syndrome in a 31-year-old woman undergoing aortic valve replacement.” J Cardiothorac Vasc Anesth 23:855–857. | 4 |

**3.6. Ear, nose and throat (ENT) surgery in patients with MPS IVA**

| Tonsillectomy and/or adenoidectomy is recommended for patients with MPS IVA who display recurrent otitis media, snoring and/or OSA as early as possible following diagnosis without waiting for disease progression  ***Evidence Grade: C (Level 2, 3 and 4 studies)*** | |
| --- | --- |
| **References** | **Evidence Level** |
| Mesolella, M., M. Cimmino, E. Cantone, A. Marino, M. Cozzolino, R. Della Casa, G. Parenti and M. Iengo (2013). "Management of otolaryngological manifestations in mucopolysaccharidoses: our experience." Acta Otorhinolaryngol Ital **33**(4): 267-272. | 4 |
| Gonuldas, B., T. Yilmaz, H. S. Sivri, K. S. Gucer, K. Kilinc, G. A. Genc, M. Kilic and T. Coskun (2014). "Mucopolysaccharidosis: Otolaryngologic findings, obstructive sleep apnea and accumulation of glucosaminoglycans in lymphatic tissue of the upper airway." Int J Pediatr Otorhinolaryngol **78**(6): 944-949. | 3 |
| Sudarsan, S. S., V. K. Paramasivan, S. V. Arumugam, S. Murali and M. Kameswaran (2014). "Comparison of treatment modalities in syndromic children with obstructive sleep apnea--a randomized cohort study." Int J Pediatr Otorhinolaryngol **78**(9): 1526-1533. | 2 |

| Insertion of ventilation tubes is recommended for patients with MPS IVA with otitis media with effusion and/or recurrent otitis media to maintain hearing and/or prevent recurrent acute otitis media  ***Evidence Grade: D (Limited published evidence)*** | |
| --- | --- |
| **References** | **Evidence Level** |
| Mesolella, M., M. Cimmino, E. Cantone, A. Marino, M. Cozzolino, R. Della Casa, G. Parenti and M. Iengo (2013). "Management of otolaryngological manifestations in mucopolysaccharidoses: our experience." Acta Otorhinolaryngol Ital **33**(4): 267-272. | 4 |
| Gonuldas, B., T. Yilmaz, H. S. Sivri, K. S. Gucer, K. Kilinc, G. A. Genc, M. Kilic and T. Coskun (2014). "Mucopolysaccharidosis: Otolaryngologic findings, obstructive sleep apnea and accumulation of glucosaminoglycans in lymphatic tissue of the upper airway." Int J Pediatr Otorhinolaryngol **78**(6): 944-949. | 3 |

| Tracheostomy is recommended in patients with MPS IVA who do not respond to any of the treatment modalities mentioned above  ***Evidence Grade: D (Limited published evidence)*** | |
| --- | --- |
| **References** | **Evidence Level** |
| Mesolella, M., M. Cimmino, E. Cantone, A. Marino, M. Cozzolino, R. Della Casa, G. Parenti and M. Iengo (2013). "Management of otolaryngological manifestations in mucopolysaccharidoses: our experience." Acta Otorhinolaryngol Ital **33**(4): 267-272. | 4 |
| Gonuldas, B., T. Yilmaz, H. S. Sivri, K. S. Gucer, K. Kilinc, G. A. Genc, M. Kilic and T. Coskun (2014). "Mucopolysaccharidosis: Otolaryngologic findings, obstructive sleep apnea and accumulation of glucosaminoglycans in lymphatic tissue of the upper airway." Int J Pediatr Otorhinolaryngol **78**(6): 944-949. | 3 |
