# Supplementary material for: Recommendations for the management of MPS VI: systematic evidence- and consensus-based guidance
Source: Orphanet J Rare Dis. 2019 May 29;14:118. doi: 10.1186/s13023-019-1080-y (PMC6541999; doi:10.1186/s13023-019-1080-y)
Supplement: Supplementary file 3 — Oxford CEBM grading for MPS VI. Tables detailing the evidence levels given to each reference supporting the MPS VI guidance statements and the Evidence Grades applied to each guidance statement. Evidence levels were assessed using the Oxford Centre for Evidence-based Medicine and were based on the quality of evidence of each reference. For each guidance statement, an overall Evidence Grade was applied, based on the evidence levels of the supporting references. (DOCX 41 kb) [file 13023_2019_1080_MOESM3_ESM.docx]

# Additional File 3. Oxford Centre for Evidence-based Medicine grading for MPS VI guidance statements

1. **DISEASE-MODIFYING INTERVENTIONS**

**1.1. ERT (galsulfase) in patients with MPS VI**

| Initiation of long-term ERT with galsulfase at a dose of 1 mg/kg/week by intravenous infusion is recommended in patients with MPS VI as soon as possible after a confirmed diagnosis  ***Evidence Grade: B*** ***(Level 2/3/4 studies)*** | |
| --- | --- |
| **References** | **Evidence Level** |
| Decker, C., Z. F. Yu, R. Giugliani, I. V. Schwartz, N. Guffon, E. L. Teles, M. C. Miranda, J. E. Wraith, M. Beck, L. Arash, M. Scarpa, D. Ketteridge, J. J. Hopwood, B. Plecko, R. Steiner, C. B. Whitley, P. Kaplan, S. J. Swiedler, S. Conrad and P. Harmatz (2010). "Enzyme replacement therapy for mucopolysaccharidosis VI: Growth and pubertal development in patients treated with recombinant human N-acetylgalactosamine 4-sulfatase." J Pediatr Rehabil Med **3**(2): 89-100. | 2 |
| Hendriksz, C. J., R. Giugliani, P. Harmatz, C. Lampe, A. M. Martins, G. M. Pastores, R. D. Steiner, E. Leao Teles, V. Valayannopoulos and C. S. P. S. Group (2013). "Design, baseline characteristics, and early findings of the MPS VI (mucopolysaccharidosis VI) Clinical Surveillance Program (CSP)." J Inherit Metab Dis **36**(2): 373-384. | 2 |
| Giugliani, R., C. Lampe, N. Guffon, D. Ketteridge, E. Leao-Teles, J. E. Wraith, S. A. Jones, C. Piscia-Nichols, P. Lin, A. Quartel and P. Harmatz (2014). "Natural history and galsulfase treatment in mucopolysaccharidosis VI (MPS VI, Maroteaux-Lamy syndrome)--10-year follow-up of patients who previously participated in an MPS VI Survey Study." Am J Med Genet A **164A**(8): 1953-1964. | 3 |
| Harmatz, P., C. J. Hendriksz, C. Lampe, J. J. McGill, R. Parini, E. Leao-Teles, V. Valayannopoulos, T. J. Cole, R. Matousek, S. Graham, N. Guffon, A. Quartel and M. V. S. Group (2017). "The effect of galsulfase enzyme replacement therapy on the growth of patients with mucopolysaccharidosis VI (Maroteaux-Lamy syndrome)." Mol Genet Metab **122**(1-2): 107-112. | 3 |
| Harmatz, P., D. Ketteridge, R. Giugliani, N. Guffon, E. L. Teles, M. C. Miranda, Z. F. Yu, S. J. Swiedler, J. J. Hopwood and M. V. S. Group (2005). "Direct comparison of measures of endurance, mobility, and joint function during enzyme-replacement therapy of mucopolysaccharidosis VI (Maroteaux-Lamy syndrome): results after 48 weeks in a phase 2 open-label clinical study of recombinant human N-acetylgalactosamine 4-sulfatase." Pediatrics **115**(6): e681-689. | 3 |
| Harmatz, P., R. Giugliani, I. Schwartz, N. Guffon, E. L. Teles, M. C. Miranda, J. E. Wraith, M. Beck, L. Arash, M. Scarpa, Z. F. Yu, J. Wittes, K. I. Berger, M. S. Newman, A. M. Lowe, E. Kakkis, S. J. Swiedler and M. V. P. S. Group (2006). "Enzyme replacement therapy for mucopolysaccharidosis VI: a phase 3, randomized, double-blind, placebo-controlled, multinational study of recombinant human N-acetylgalactosamine 4-sulfatase (recombinant human arylsulfatase B or rhASB) and follow-on, open-label extension study." J Pediatr **148**(4): 533-539. | 2 |
| McGill, J. J., A. C. Inwood, D. J. Coman, M. L. Lipke, D. de Lore, S. J. Swiedler and J. J. Hopwood (2010). "Enzyme replacement therapy for mucopolysaccharidosis VI from 8 weeks of age--a sibling control study." Clin Genet **77**(5): 492-498 | 4 |
| But, W. M., M. Y. Wong, J. C. Chow, W. K. Chan, W. T. Ko, S. P. Wu, M. L. Wong, T. Y. Miu, W. Y. Tse, W. W. Hung, T. W. Fan and C. C. Shek (2011). "Enzyme replacement therapy for mucopolysaccharidosis VI (Maroteaux-Lamy syndrome): experience in Hong Kong." Hong Kong Med J **17**(4): 317-324. | 4 |
| Brunelli, M. J., A. N. Atallah and E. M. da Silva (2016). "Enzyme replacement therapy with galsulfase for mucopolysaccharidosis type VI." Cochrane Database Syst Rev **3**: CD009806. | 1 |
| Braunlin, E., H. Rosenfeld, C. Kampmann, J. Johnson, M. Beck, R. Giugliani, N. Guffon, D. Ketteridge, C. M. Sa Miranda, M. Scarpa, I. V. Schwartz, E. Leao Teles, J. E. Wraith, P. Barrios, E. Dias da Silva, G. Kurio, M. Richardson, G. Gildengorin, J. J. Hopwood, M. Imperiale, A. Schatz, C. Decker, P. Harmatz and M. V. S. Group (2013). "Enzyme replacement therapy for mucopolysaccharidosis VI: long-term cardiac effects of galsulfase (Naglazyme(R)) therapy." J Inherit Metab Dis **36**(2): 385-394. | 3 |
| Horovitz, D. D., T. S. Magalhaes, A. Acosta, E. M. Ribeiro, L. R. Giuliani, D. B. Palhares, C. A. Kim, A. C. de Paula, M. Kerstenestzy, M. A. Pianovski, M. I. Costa, F. C. Santos, A. M. Martins, C. S. Aranda, J. Correa Neto, G. B. Holanda, L. Cardoso, Jr., C. A. da Silva, R. C. Bonatti, B. F. Ribeiro, C. Rodrigues Mdo and J. C. Llerena, Jr. (2013). "Enzyme replacement therapy with galsulfase in 34 children younger than five years of age with MPS VI." Mol Genet Metab **109**(1): 62-69. | 3 |
| Brands, M. M., M. Hoogeveen-Westerveld, M. A. Kroos, W. Nobel, G. J. Ruijter, L. Ozkan, I. Plug, D. Grinberg, L. Vilageliu, D. J. Halley, A. T. van der Ploeg and A. J. Reuser (2013). "Mucopolysaccharidosis type VI phenotypes-genotypes and antibody response to galsulfase." Orphanet J Rare Dis **8**: 51. | 2 |
| Brands, M. M., E. Oussoren, G. J. Ruijter, A. A. Vollebregt, H. M. van den Hout, K. F. Joosten, W. C. Hop, I. Plug and A. T. van der Ploeg (2013). "Up to five years experience with 11 mucopolysaccharidosis type VI patients." Mol Genet Metab **109**(1): 70-76. | 3 |
| Harmatz, P. R., P. Garcia, N. Guffon, L. M. Randolph, R. Shediac, E. Braunlin, R. S. Lachman and C. Decker (2014). "Galsulfase (Naglazyme(R)) therapy in infants with mucopolysaccharidosis VI." J Inherit Metab Dis **37**(2): 277-287. | 4 |
| Jurecka, A., V. Opoka-Winiarska, E. Jurkiewicz, J. Marucha and A. Tylki-Szymanska (2012). "Spinal cord compression in Maroteaux-Lamy syndrome: case report and review of the literature with effects of enzyme replacement therapy." Pediatr Neurosurg **48**(3): 191-198. | 4 |
| Horovitz, D. D. G., A. X. Acosta, L. de Rosso Giuliani and E. M. Ribeiro (2015). "Mucopolysaccharidosis type VI on enzyme replacement therapy since infancy: Six years follow-up of four children." Mol Genet Metab Rep **5**: 19-25. | 4 |
| Furujo, M., T. Kubo, M. Kosuga and T. Okuyama (2011). "Enzyme replacement therapy attenuates disease progression in two Japanese siblings with mucopolysaccharidosis type VI." Mol Genet Metab **104**(4): 597-602. | 4 |

**1.2 HSCT in patients with MPS VI**

| With consideration of the associated risks of morbidity and mortality associated with this procedure, HSCT may be an option for patients with MPS VI who have a matched related donor (or unrelated donor), or cord blood graft  ***Evidence Grade: C (Consistent with level 4 studies and extrapolations from Level 3 studies)*** | |
| --- | --- |
| **References** | **Evidence Level** |
| Boelens, J. J., R. F. Wynn, A. O'Meara, P. Veys, Y. Bertrand, G. Souillet, J. E. Wraith, A. Fischer, M. Cavazzana-Calvo, K. W. Sykora, P. Sedlacek, A. Rovelli, C. S. Uiterwaal and N. Wulffraat (2007). "Outcomes of hematopoietic stem cell transplantation for Hurler's syndrome in Europe: a risk factor analysis for graft failure." Bone Marrow Transplant **40**(3): 225-233. | Extrapolation from MPS I – Level 4 |
| Boelens, J. J., M. Aldenhoven, D. Purtill, A. Ruggeri, T. Defor, R. Wynn, E. Wraith, M. Cavazzana-Calvo, A. Rovelli, A. Fischer, J. Tolar, V. K. Prasad, M. Escolar, E. Gluckman, A. O'Meara, P. J. Orchard, P. Veys, M. Eapen, J. Kurtzberg, V. Rocha, Eurocord, B. Inborn Errors Working Party of European, g. Marrow Transplant, B. Duke University, P. Marrow Transplantation, B. Centre for International and R. Marrow (2013). "Outcomes of transplantation using various hematopoietic cell sources in children with Hurler syndrome after myeloablative conditioning." Blood 121(19): 3981-3987. | Extrapolation from MPS I – Level 4 |
| Herskhovitz, E., E. Young, J. Rainer, C. M. Hall, V. Lidchi, K. Chong and A. Vellodi (1999). "Bone marrow transplantation for Maroteaux-Lamy syndrome (MPS VI): long-term follow-up." J Inherit Metab Dis **22**(1): 50-62. | 4 |
| Turbeville, S., H. Nicely, J. D. Rizzo, T. L. Pedersen, P. J. Orchard, M. E. Horwitz, E. M. Horwitz, P. Veys, C. Bonfim and A. Al-Seraihy (2011). "Clinical outcomes following hematopoietic stem cell transplantation for the treatment of mucopolysaccharidosis VI." Mol Genet Metab **102**(2): 111-115.  Valayannopoulos, V., H. Nicely, P. Harmatz and S. Turbeville (2010). "Mucopolysaccharidosis VI." Orphanet J Rare Dis **5**: 5. | 3 |
| Jester, S., J. Larsson, E. A. Eklund, D. Papadopoulou, J. E. Mansson, A. N. Bekassy, D. Turkiewicz, J. Toporski and I. Ora (2013). "Haploidentical stem cell transplantation in two children with mucopolysaccharidosis VI: clinical and biochemical outcome." Orphanet J Rare Dis **8**: 134. | 4 |
| Gullingsrud, E. O., W. Krivit and C. G. Summers (1998). "Ocular abnormalities in the mucopolysaccharidoses after bone marrow transplantation. Longer follow-up." Ophthalmology **105**(6): 1099-1105. | 3 |
| Patel, P., Y. Suzuki, A. Tanaka, H. Yabe, S. Kato, T. Shimada, R. W. Mason, K. E. Orii, T. Fukao, T. Orii and S. Tomatsu (2014). "Impact of Enzyme Replacement Therapy and Hematopoietic Stem Cell Therapy on Growth in Patients with Hunter Syndrome." Mol Genet Metab Rep **1**: 184-196. | Extrapolation from MPS I - Level 3 |
| Ghosh, A., W. Miller, P. J. Orchard, S. A. Jones, J. Mercer, H. J. Church, K. Tylee, T. Lund, B. W. Bigger, J. Tolar and R. F. Wynn (2016). "Enzyme replacement therapy prior to haematopoietic stem cell transplantation in Mucopolysaccharidosis Type I: 10 year combined experience of 2 centres." Mol Genet Metab **117**(3): 373-377. | Extrapolation from MPS I - Level 3 |
| Aldenhoven, M., S. Jones, D. Bonney, R. Borrill, M. Coussons, J. Mercer, M. Bierings, B. Versluys, P. van Hasselt, F. Wijburg, A. van der Ploeg, R. Wynn and J. Boelens (2015). "Hematopoietic Cell Transplantation for MPS Patients Is Safe and Effective: Results after Implementation of International Guidelines." Biol Blood Marrow Transplant **21**(2): S93 | Extrapolation from MPS I, II, III and VI - Level 4 |
| Summers, C. G., R. L. Purple, W. Krivit, R. Pineda, 2nd, G. T. Copland, N. K. Ramsay, J. H. Kersey and C. B. Whitley (1989). "Ocular changes in the mucopolysaccharidoses after bone marrow transplantation. A preliminary report." Ophthalmology **96**(7): 977-984; discussion 984-975. | 4 |

| Due to the risk of mortality, it is critical that HSCT is only performed in an institution with a multidisciplinary team experienced in the care of patients with MPS VI  ***Evidence Grade: C (Level 3/4 studies with inconsistent risk/benefit results)*** | |
| --- | --- |
| **References** | **Evidence Level** |
| Boelens, J. J., R. F. Wynn, A. O'Meara, P. Veys, Y. Bertrand, G. Souillet, J. E. Wraith, A. Fischer, M. Cavazzana-Calvo, K. W. Sykora, P. Sedlacek, A. Rovelli, C. S. Uiterwaal and N. Wulffraat (2007). "Outcomes of hematopoietic stem cell transplantation for Hurler's syndrome in Europe: a risk factor analysis for graft failure." Bone Marrow Transplant **40**(3): 225-233. | Extrapolation from MPS I – Level 4 |
| Boelens, J. J., M. Aldenhoven, D. Purtill, A. Ruggeri, T. Defor, R. Wynn, E. Wraith, M. Cavazzana-Calvo, A. Rovelli, A. Fischer, J. Tolar, V. K. Prasad, M. Escolar, E. Gluckman, A. O'Meara, P. J. Orchard, P. Veys, M. Eapen, J. Kurtzberg, V. Rocha, Eurocord, B. Inborn Errors Working Party of European, g. Marrow Transplant, B. Duke University, P. Marrow Transplantation, B. Centre for International and R. Marrow (2013). "Outcomes of transplantation using various hematopoietic cell sources in children with Hurler syndrome after myeloablative conditioning." Blood 121(19): 3981-3987. | Extrapolation from MPS I – Level 4 |
| Herskhovitz, E., E. Young, J. Rainer, C. M. Hall, V. Lidchi, K. Chong and A. Vellodi (1999). "Bone marrow transplantation for Maroteaux-Lamy syndrome (MPS VI): long-term follow-up." J Inherit Metab Dis **22**(1): 50-62. | 4 |
| Turbeville, S., H. Nicely, J. D. Rizzo, T. L. Pedersen, P. J. Orchard, M. E. Horwitz, E. M. Horwitz, P. Veys, C. Bonfim and A. Al-Seraihy (2011). "Clinical outcomes following hematopoietic stem cell transplantation for the treatment of mucopolysaccharidosis VI." Mol Genet Metab **102**(2): 111-115.  Valayannopoulos, V., H. Nicely, P. Harmatz and S. Turbeville (2010). "Mucopolysaccharidosis VI." Orphanet J Rare Dis **5**: 5. | 3 |
| Jester, S., J. Larsson, E. A. Eklund, D. Papadopoulou, J. E. Mansson, A. N. Bekassy, D. Turkiewicz, J. Toporski and I. Ora (2013). "Haploidentical stem cell transplantation in two children with mucopolysaccharidosis VI: clinical and biochemical outcome." Orphanet J Rare Dis **8**: 134. | 4 |
| Gullingsrud, E. O., W. Krivit and C. G. Summers (1998). "Ocular abnormalities in the mucopolysaccharidoses after bone marrow transplantation. Longer follow-up." Ophthalmology **105**(6): 1099-1105. | 3 |
| Patel, P., Y. Suzuki, A. Tanaka, H. Yabe, S. Kato, T. Shimada, R. W. Mason, K. E. Orii, T. Fukao, T. Orii and S. Tomatsu (2014). "Impact of Enzyme Replacement Therapy and Hematopoietic Stem Cell Therapy on Growth in Patients with Hunter Syndrome." Mol Genet Metab Rep **1**: 184-196. | Extrapolation from MPS I - Level 3 |
| Ghosh, A., W. Miller, P. J. Orchard, S. A. Jones, J. Mercer, H. J. Church, K. Tylee, T. Lund, B. W. Bigger, J. Tolar and R. F. Wynn (2016). "Enzyme replacement therapy prior to haematopoietic stem cell transplantation in Mucopolysaccharidosis Type I: 10 year combined experience of 2 centres." Mol Genet Metab **117**(3): 373-377. | Extrapolation from MPS I - Level 3 |
| Aldenhoven, M., S. Jones, D. Bonney, R. Borrill, M. Coussons, J. Mercer, M. Bierings, B. Versluys, P. van Hasselt, F. Wijburg, A. van der Ploeg, R. Wynn and J. Boelens (2015). "Hematopoietic Cell Transplantation for MPS Patients Is Safe and Effective: Results after Implementation of International Guidelines." Biol Blood Marrow Transplant **21**(2): S93 | Extrapolation from MPS I, II, III and VI - Level 4 |
| Summers, C. G., R. L. Purple, W. Krivit, R. Pineda, 2nd, G. T. Copland, N. K. Ramsay, J. H. Kersey and C. B. Whitley (1989). "Ocular changes in the mucopolysaccharidoses after bone marrow transplantation. A preliminary report." Ophthalmology **96**(7): 977-984; discussion 984-975. | 4 |

**2. INTERVENTIONS TO SUPPORT RESPIRATORY AND SLEEP DISORDERS**

**2.1. Respiratory interventions and sleep disorders in patients with MPS VI**

| CPAP therapy is recommended for patients with MPS VI who display the presence of obstructive sleep apnoea (OSA) that persists after tonsillectomy and/or adenoidectomy  ***Evidence Grade: B (Extrapolations from level 1 studies)*** | |
| --- | --- |
| **References** | **Evidence Level** |
| Leighton, S. E., B. Papsin, A. Vellodi, R. Dinwiddie and R. Lane (2001). "Disordered breathing during sleep in patients with mucopolysaccharidoses." Int J Pediatr Otorhinolaryngol **58**(2): 127-138. | 3 |
| Giles TL, Lasserson TJ, Smith B, White J, Wright JJ, Cates CJ. (2006) “Continuous positive airways pressure for obstructive sleep apnoea in adults.” Cochrane Database of Systematic Reviews (3) | Extrapolation from systematic review of sleep apnea patients - Level 1 |
| Faria DAS, da Silva EMK, Atallah ÁN, Vital FMR (2006) “Continuous positive airways pressure for obstructive sleep apnoea in adults.” Cochrane Database Syst Rev. (3) | Extrapolation from systematic review of OSA patients - Level 1 |

| NIPPV therapy is recommended for patients with MPS VI who display nocturnal hypoventilation and are unresponsive to CPAP, or display daytime hypoventilation with increased PaCO_2_ and/or serum HCO_3_ levels  ***Evidence Grade: B (Extrapolations from Level 1 studies)*** | |
| --- | --- |
| **References** | **Evidence Level** |
| Ram FS, Picot J, Lightowler J, Wedzicha JA. (2004) “Non-invasive positive pressure ventilation for treatment of respiratory failure due to exacerbations of chronic obstructive pulmonary disease.” Cochrane Database Syst Rev. (1) | Extrapolation from systematic review of COPD patients - Level 1 |
| Lim WJ, Mohammed Akram R, Carson KV, Mysore S, Labiszewski NA, Wedzicha JA, Rowe BH, Smith BJ. (2012) “Non-invasive positive pressure ventilation for treatment of respiratory failure due to severe acute exacerbations of asthma.” Cochrane Database Syst Rev. 12 | Extrapolation from systematic review of severe acute asthma patients - Level 1 |

| Oxygen supplementation during sleep is recommended for patients with MPS VI who display sleep apnoea with nocturnal hypoxemia, and who do not tolerate CPAP or NIPPV masks  ***Evidence Grade: B (Extrapolations from Level 1/3/4 studies)*** | |
| --- | --- |
| **References** | **Evidence Level** |
| John, A., S. Fagondes, I. Schwartz, A. C. Azevedo, P. Barrios, P. Dalcin, S. Menna-Barreto and R. Giugliani (2011). "Sleep abnormalities in untreated patients with mucopolysaccharidosis type VI." Am J Med Genet A **155A**(7): 1546-1551. | 3 |
| Montano, A. M., S. Tomatsu, G. S. Gottesman, M. Smith and T. Orii (2007). "International Morquio A Registry: clinical manifestation and natural course of Morquio A disease." J Inherit Metab Dis **30**(2): 165-174. | Extrapolation from MPS IVA -Level 4 |
| Vanita Mehta, M.D., Tajender S. Vasu, M.D., M.S., Barbara Phillips, M.D., F.A.A.S.M., and Frances Chung, M.B.B.S. (2013) “Obstructive Sleep Apnea and Oxygen Therapy: A Systematic Review of the Literature and Meta-Analysis” J Clin Sleep Med. 2013 Mar 15; 9(3): 271–279. | Extrapolation from systematic review of OSA patients - Level 1 |

| Patients with MPS VI should be monitored for development of hypercapnia after starting oxygen therapy using measurement of PaCO_2_ and/or serum HCO_3_  ***Evidence Grade: D (Level 5, expert clinical opinion)*** | |
| --- | --- |
| **References** | **Evidence Level** |
| Giugliani, R., P. Harmatz and J. E. Wraith (2007). "Management guidelines for mucopolysaccharidosis VI." Pediatrics **120**(2): 405-418. | N/A |
| Piper, A. J. and B. J. Yee (2014). "Hypoventilation syndromes." Compr Physiol **4**(4): 1639-1676. | N/A |

**3. ANAESTHESIA AND SURGICAL INTERVENTIONS**

**3.1. Anaesthesia in patients with MPS VI**

| Pre-, intra- and post-operative care (until extubation is complete) for all procedures requiring general anaesthesia, conscious or deep sedation, should be supervised by an anaesthetist with experience in treating patients with MPS and/or complex airway management. In addition, the anaesthetist should have access to Intensive Care support and be surrounded by an experienced team capable of performing emergency tracheotomy if required  ***Evidence Grade: C*** ***(Level 3/4 studies)*** | |
| --- | --- |
| **References** | **Evidence Level** |
| Moores, C., J. G. Rogers, I. M. McKenzie and T. C. Brown (1996). "Anaesthesia for children with mucopolysaccharidoses." Anaesth Intensive Care **24**(4): 459-463. | 4 |
| Walker, R. W., M. Darowski, P. Morris and J. E. Wraith (1994). "Anaesthesia and mucopolysaccharidoses. A review of airway problems in children." Anaesthesia 49(12): 1078-1084. | 3 |
| Frawley, G., D. Fuenzalida, S. Donath, J. Yaplito-Lee and H. Peters (2012). "A retrospective audit of anesthetic techniques and complications in children with mucopolysaccharidoses." Paediatr Anaesth **22**(8): 737-744. | 3 |
| Walker, R. W., D. L. Allen and M. R. Rothera (1997). "A fibreoptic intubation technique for children with mucopolysaccharidoses using the laryngeal mask airway." Paediatr Anaesth **7**(5): 421-426. | 4 |
| Walker, R. W., V. Colovic, D. N. Robinson and O. R. Dearlove (2003). "Postobstructive pulmonary oedema during anaesthesia in children with mucopolysaccharidoses." Paediatr Anaesth **13**(5): 441-447. | 4 |
| Herrick, I. A. and E. J. Rhine (1988). "The mucopolysaccharidoses and anaesthesia: a report of clinical experience." Can J Anaesth **35**(1): 67-73. | 4 |

| A full assessment of the risks and benefits should take place with the patient and family prior to any procedure. All pre-operative information should be made available to allow decision making  ***Evidence Grade: C*** ***(Level 4 study and extrapolation from Level 3 study)*** | |
| --- | --- |
| **References** | **Evidence Level** |
| Moores, C., J. G. Rogers, I. M. McKenzie and T. C. Brown (1996). "Anaesthesia for children with mucopolysaccharidoses." Anaesth Intensive Care **24**(4): 459-463. | 4 |
| Walker, R. W., M. Darowski, P. Morris and J. E. Wraith (1994). "Anaesthesia and mucopolysaccharidoses. A review of airway problems in children." Anaesthesia 49(12): 1078-1084. | 3 |

| ENT respiratory, cardiac, and radiological assessment should be performed prior to any procedure requiring anesthesia  ***Evidence Grade: C*** ***(Level 3 study and extrapolation from Level 3 study)*** | |
| --- | --- |
| **References** | **Evidence Level** |
| Walker, R. W., M. Darowski, P. Morris and J. E. Wraith (1994). "Anaesthesia and mucopolysaccharidoses. A review of airway problems in children." Anaesthesia 49(12): 1078-1084. | 3 |
| Theroux, M. C., T. Nerker, C. Ditro and W. G. Mackenzie (2012). "Anesthetic care and perioperative complications of children with Morquio syndrome." Paediatr Anaesth **22**(9): 901-907. | Extrapolation from MPS IVA – Level 3 |

| It is critical to maintain a neutral neck position during all surgeries, and during intubation and extubation to avoid paralysis. Strongly recommend the use of techniques that allow maintenance of the neutral neck position, including use of laryngeal mask airway (LMA) for shorter procedures, or intubation with a video laryngoscope or fibreoptic intubation  ***Evidence Grade: C*** ***(Level 3/4 studies)*** | |
| --- | --- |
| **References** | **Evidence Level** |
| Moores, C., J. G. Rogers, I. M. McKenzie and T. C. Brown (1996). "Anaesthesia for children with mucopolysaccharidoses." Anaesth Intensive Care **24**(4): 459-463. | 4 |
| Walker, R. W., M. Darowski, P. Morris and J. E. Wraith (1994). "Anaesthesia and mucopolysaccharidoses. A review of airway problems in children." Anaesthesia 49(12): 1078-1084. | 3 |
| Frawley, G., D. Fuenzalida, S. Donath, J. Yaplito-Lee and H. Peters (2012). "A retrospective audit of anesthetic techniques and complications in children with mucopolysaccharidoses." Paediatr Anaesth **22**(8): 737-744. | 3 |
| Walker, R. W., D. L. Allen and M. R. Rothera (1997). "A fibreoptic intubation technique for children with mucopolysaccharidoses using the laryngeal mask airway." Paediatr Anaesth **7**(5): 421-426. | 4 |
| Walker, R. W., V. Colovic, D. N. Robinson and O. R. Dearlove (2003). "Postobstructive pulmonary oedema during anaesthesia in children with mucopolysaccharidoses." Paediatr Anaesth **13**(5): 441-447. | 4 |
| Theroux, M. C., T. Nerker, C. Ditro and W. G. Mackenzie (2012). "Anesthetic care and perioperative complications of children with Morquio syndrome." Paediatr Anaesth **22**(9): 901-907. | Extrapolation from MPS IVA - Level 3 |

| Pre-operative and intra-operative measures to avoid hypotension should be adopted during all surgical procedures in patients with MPS VI to maintain spinal cord perfusion and therefore protect spinal cord function  ***Evidence Grade: D (Expert clinical opinion)*** | |
| --- | --- |
| **References** | **Evidence Level** |
|  |  |

| Intra-operative neurophysiological monitoring (including somatosensory evoked potentials [SSEP], electromyography [EMG] and motor evoked potentials [MEP]) is strongly recommended during all spinal surgeries and other potentially lengthy or complicated procedures, including those that require manipulation of the head and neck  ***Evidence Grade: D (Limited published evidence)*** | |
| --- | --- |
| **References** | **Evidence Level** |
| Theroux, M. C., T. Nerker, C. Ditro and W. G. Mackenzie (2012). "Anesthetic care and perioperative complications of children with Morquio syndrome." Paediatr Anaesth **22**(9): 901-907. | Extrapolation from MPS IVA: 3 |
| Walker, R., K. G. Belani, E. A. Braunlin, I. A. Bruce, H. Hack, P. R. Harmatz, S. Jones, R. Rowe, G. A. Solanki and B. Valdemarsson (2013). "Anaesthesia and airway management in mucopolysaccharidosis." J Inherit Metab Dis **36**(2): 211-219. | N/A |
| Giugliani, R., P. Harmatz and J. E. Wraith (2007). "Management guidelines for mucopolysaccharidosis VI." Pediatrics **120**(2): 405-418. | N/A |

| For other surgeries and procedures, neurophysiologic monitoring should be considered based on pre-existing risk for spinal cord compression and instability, need for spine manipulation, possibility of hemodynamic changes and blood loss, or extended length of time  ***Evidence Grade: D (Limited published evidence)*** | |
| --- | --- |
| **References** | **Evidence Level** |
| Theroux, M. C., T. Nerker, C. Ditro and W. G. Mackenzie (2012). "Anesthetic care and perioperative complications of children with Morquio syndrome." Paediatr Anaesth **22**(9): 901-907. | Extrapolation from MPS IVA: 3 |

| Intrathecal and epidural techniques should be used with extreme caution in patients with MPS VI, due to the anatomical challenges of very short stature, as well as spinal abnormalities causing insertion problems and unpredictability of spread of local anaesthesia. However, these techniques may be considered to avoid general anaesthesia in a high-risk situation or pregnancy  ***Evidence Grade: D (Expert clinical opinion)*** | |
| --- | --- |
| **References** | **Evidence Level** |
|  |  |

**3.2. Limb surgeries in patients with MPS VI**

| Hip replacement can be considered in adult patients with MPS VI who exhibit hip pain, reduced walking and endurance related to hip disease, as well as abnormal radiographic findings  ***Evidence Grade: D (Limited published evidence)*** | |
| --- | --- |
| **References** | **Evidence Level** |
| Oussoren, E., J. Bessems, V. Pollet, J. C. van der Meijden, L. J. van der Giessen, I. Plug, A. S. Devos, G. J. G. Ruijter, A. T. van der Ploeg and M. Langeveld (2017). "A long term follow-up study of the development of hip disease in Mucopolysaccharidosis type VI." Mol Genet Metab **121**(3): 241-251. | 3 |

| Hip reconstruction is not routinely indicated, but may be considered in paediatric patients with MPS VI who exhibit hip pain, reduced walking and endurance related to hip disease, as well as abnormal radiographic findings  ***Evidence Grade: D (Limited published evidence)*** | |
| --- | --- |
| **References** | **Evidence Level** |
| Oussoren, E., J. Bessems, V. Pollet, J. C. van der Meijden, L. J. van der Giessen, I. Plug, A. S. Devos, G. J. G. Ruijter, A. T. van der Ploeg and M. Langeveld (2017). "A long term follow-up study of the development of hip disease in Mucopolysaccharidosis type VI." Mol Genet Metab **121**(3): 241-251. | 3 |

| Growth modulation surgery is recommended in patients with MPS VI who have signs of genu valgum and should be performed as early as possible during the period of growth  ***Evidence Grade: D (Limited published evidence)*** | |
| --- | --- |
| **References** | **Evidence Level** |
| Wood M, D. JE, M. A and E. DM (2017). "Guided growth surgery for genu valgum in mucopolysaccharidosis type VI." Mol Genet Metab **120**(1-2): S141 | 4 |

**3.3. Spinal surgeries in patients with MPS VI**

| Decompression of the spinal cord is recommended in patients with MPS VI who have evidence of spinal cord compression based on clinical and radiographic findings  ***Evidence Grade: D (Limited published evidence)*** | |
| --- | --- |
| **References** | **Evidence Level** |
| Solanki, G. A., P. P. Sun, K. W. Martin, C. J. Hendriksz, C. Lampe, N. Guffon, A. Hung, Z. Sisic, R. Shediac, P. R. Harmatz and C. S. P. S. Group (2016). "Cervical cord compression in mucopolysaccharidosis VI (MPS VI): Findings from the MPS VI Clinical Surveillance Program (CSP)." Mol Genet Metab **118**(4): 310-318. | 3 |

| Spinal stabilization of the craniocervical junction with either cervical fusion or occipital-cervical fusion is recommended in patients with MPS VI who have evidence of instability  ***Evidence Grade: D (Expert clinical opinion)*** | |
| --- | --- |
| **References** | **Evidence level** |
|  |  |

| Correction of thoracolumbar kyphoscoliosis is recommended in patients with MPS VI who present with progressive radiographic changes, intractable pain and clinical deterioration as defined by gait, lung function and changes in the degree of kyphosis  ***Evidence Grade: D (Limited published evidence)*** | |
| --- | --- |
| **References** | **Evidence Level** |
| Dalvie, S. S., M. H. Noordeen and A. Vellodi (2001). "Anterior instrumented fusion for thoracolumbar kyphosis in mucopolysaccharidosis." Spine (Phila Pa 1976) **26**(23): E539-541. | 4 |
| Brands, M. M., E. Oussoren, G. J. Ruijter, A. A. Vollebregt, H. M. van den Hout, K. F. Joosten, W. C. Hop, I. Plug and A. T. van der Ploeg (2013). "Up to five years experience with 11 mucopolysaccharidosis type VI patients." Mol Genet Metab **109**(1): 70-76. | 3 |

**3.4. Ophthalmic surgery in patients with MPS VI**

| Corneal transplantation can be considered for patients with MPS VI who have significant visual loss attributed to corneal opacification  ***Evidence Grade: B (Extrapolations from level 1/3/4 studies)*** | |
| --- | --- |
| **References** | **Evidence Level** |
| Bothun, E. D., A. Decanini, C. G. Summers, P. J. Orchard and J. Tolar (2011). "Outcome of penetrating keratoplasty for mucopolysaccharidoses." Arch Ophthalmol **129**(2): 138-144. | Extrapolation from MPS I, IV and VI - Level 4 |
| Ohden, K. L., S. Pitz, J. Ashworth, A. Magalhaes, D. R. Marinho, P. Lindahl, K. Tear Fahnehjelm and C. G. Summers (2017). "Outcomes of keratoplasty in the mucopolysaccharidoses: an international perspective." Br J Ophthalmol **101**(7): 909-912. | Extrapolation from MPS I, IV and VI - Level 3 |
| Schwartz, M. F., T. P. Werblin and W. R. Green (1985). "Occurrence of mucopolysaccharide in corneal grafts in the Maroteaux-Lamy syndrome." Cornea **4**(1): 58-66. | Extrapolation from MPS I, IV and VI - Level 4 |
| Keane, M., D. Coster, M. Ziaei and K. Williams (2014). "Deep anterior lamellar keratoplasty versus penetrating keratoplasty for treating keratoconus." Cochrane Database Syst Rev(7): CD009700. | Extrapolation from level 1 systematic review (general population) |

**3.5. Carpal tunnel decompression in patients with MPS VI**

| Decompression of the median nerve and tenosynovectomy of all flexor tendons in the carpal tunnel is recommended in patients with MPS VI who display flexion contractures and distal interphalangeal (DIP) joints and/or proximal interphalangeal (PIP) joints (clawing), as well as clinical symptoms of hand pain and/or numbness in the thumb to middle finger, and in patients with positive nerve conduction studies  ***Evidence Grade: C (Level 4 studies)*** | |
| --- | --- |
| **References** | **Evidence Level** |
| Van Heest, A. E., J. House, W. Krivit and K. Walker (1998). "Surgical treatment of carpal tunnel syndrome and trigger digits in children with mucopolysaccharide storage disorders." J Hand Surg Am **23**(2): 236-243. | 4 |
| Yuen, A., G. Dowling, B. Johnstone, A. Kornberg and C. Coombs (2007). "Carpal tunnel syndrome in children with mucopolysaccaridoses." J Child Neurol **22**(3): 260-263. | 4 |

| A1 and A3 pulley release is recommended in patients with MPS VI who display obvious trigger finger  ***Evidence Grade: D (Extrapolation from Level 4 studies)*** | |
| --- | --- |
| **References** | **Evidence Level** |
| Van Heest, A. E., J. House, W. Krivit and K. Walker (1998). "Surgical treatment of carpal tunnel syndrome and trigger digits in children with mucopolysaccharide storage disorders." J Hand Surg Am **23**(2): 236-243. | Extrapolation based on MPS I, III and VI - Level 4 |
| Yuen, A., G. Dowling, B. Johnstone, A. Kornberg and C. Coombs (2007). "Carpal tunnel syndrome in children with mucopolysaccaridoses." J Child Neurol **22**(3): 260-263. | Extrapolation based on MPS I, II and VI - Level 4 |

**3.6. Cardio-thoracic surgery in patients with MPS VI**

| Cardiac (aortic, mitral) valve replacement should be considered in patients with MPS VI who display symptomatic and severe valve stenosis or regurgitation  ***Evidence Grade: C (Level 4 studies)*** | |
| --- | --- |
| **References** | **Evidence Level** |
| Marwick, T. H., B. Bastian, C. F. Hughes and B. P. Bailey (1992). "Mitral stenosis in the Maroteaux-Lamy syndrome: a treatable cause of dyspnoea." Postgrad Med J **68**(798): 287-288 | 4 |
| Tan, C. T., H. V. Schaff, F. A. Miller, Jr., W. D. Edwards and P. S. Karnes (1992). "Valvular heart disease in four patients with Maroteaux-Lamy syndrome." Circulation **85**(1): 188-195. | 4 |
| Thumler, A., E. Miebach, C. Lampe, S. Pitz, W. Kamin, C. Kampmann, B. Link and E. Mengel (2012). "Clinical characteristics of adults with slowly progressing mucopolysaccharidosis VI: a case series." J Inherit Metab Dis **35**(6): 1071-1079. | 4 |
| Hachida, M., M. Nonoyama, Y. Bonkohara, N. Hanayama and H. Koyanagi (1996). "Combined aortic and mitral valve replacement in an adult with mucopolysaccharidosis (Maroteaux-Lamy syndrome)." Heart Vessels **11**(4): 215-217. | 4 |
| Torre, S., M. Scarpelli, A. Salviati, E. Buffone, G. Faggian and G. B. Luciani (2016). "Aortic and Mitral Valve Involvement in Maroteaux-Lamy Syndrome VI: Surgical Implications in the Enzyme Replacement Therapy Era." Ann Thorac Surg **102**(1): e23-25. | 4 |

| Left ventricular apical aneurysms occur rarely in patients with MPS VI, but should be resected whenever possible  ***Evidence Grade: D (Limited published evidence)*** | |
| --- | --- |
| **References** | **Evidence Level** |
| Oudit, G. Y., J. Butany, W. G. Williams, S. C. Siu, J. T. Clarke and R. M. Iwanochko (2007). "Left ventricular aneurysm in a patient with mucopolysaccharidosis type VI (Maroteaux-Lamy syndrome): clinical and pathological correlation." Cardiovasc Pathol **16**(4): 237-240. | 4 |

**3.7. Ear, nose and throat surgery in patients with MPS VI**

| Tonsillectomy and/or adenoidectomy is recommended in patients with MPS VI who display upper airway obstruction, recurrent otitis media, snoring and/or OSA, as early as possible following diagnosis without waiting for disease progression  ***Evidence Grade: C (Level 2/3/4 studies)*** | |
| --- | --- |
| **References** | **Evidence Level** |
| Mesolella, M., M. Cimmino, E. Cantone, A. Marino, M. Cozzolino, R. Della Casa, G. Parenti and M. Iengo (2013). "Management of otolaryngological manifestations in mucopolysaccharidoses: our experience." Acta Otorhinolaryngol Ital **33**(4): 267-272. | 4 |
| Gonuldas, B., T. Yilmaz, H. S. Sivri, K. S. Gucer, K. Kilinc, G. A. Genc, M. Kilic and T. Coskun (2014). "Mucopolysaccharidosis: Otolaryngologic findings, obstructive sleep apnea and accumulation of glucosaminoglycans in lymphatic tissue of the upper airway." Int J Pediatr Otorhinolaryngol **78**(6): 944-949. | 3 |
| Sudarsan, S. S., V. K. Paramasivan, S. V. Arumugam, S. Murali and M. Kameswaran (2014). "Comparison of treatment modalities in syndromic children with obstructive sleep apnea--a randomized cohort study." Int J Pediatr Otorhinolaryngol **78**(9): 1526-1533. | 2 |

| Tracheostomy is recommended in patients with MPS VI who exhibit severe upper airway obstruction that cannot be treated by an alternative approach, and in patients with severe sleep apnoea that is not treatable by CPAP or tonsillectomy and/or adenoidectomy  ***Evidence Grade: D (Limited published evidence)*** | |
| --- | --- |
| **References** | **Evidence Level** |
| Tan, C. T., H. V. Schaff, F. A. Miller, Jr., W. D. Edwards and P. S. Karnes (1992). "Valvular heart disease in four patients with Maroteaux-Lamy syndrome." Circulation **85**(1): 188-195. | 4 |
| Thumler, A., E. Miebach, C. Lampe, S. Pitz, W. Kamin, C. Kampmann, B. Link and E. Mengel (2012). "Clinical characteristics of adults with slowly progressing mucopolysaccharidosis VI: a case series." J Inherit Metab Dis **35**(6): 1071-1079. | 4 |

| Insertion of ventilation tubes is recommended in patients with MPS VI with otitis media with effusion and/or recurrent otitis media to maintain hearing and/or prevent recurrent acute otitis media  ***Evidence Grade: D (Limited published evidence)*** | |
| --- | --- |
| **References** | **Evidence Level** |
| Mesolella, M., M. Cimmino, E. Cantone, A. Marino, M. Cozzolino, R. Della Casa, G. Parenti and M. Iengo (2013). "Management of otolaryngological manifestations in mucopolysaccharidoses: our experience." Acta Otorhinolaryngol Ital **33**(4): 267-272. | 4 |
| Gonuldas, B., T. Yilmaz, H. S. Sivri, K. S. Gucer, K. Kilinc, G. A. Genc, M. Kilic and T. Coskun (2014). "Mucopolysaccharidosis: Otolaryngologic findings, obstructive sleep apnea and accumulation of glucosaminoglycans in lymphatic tissue of the upper airway." Int J Pediatr Otorhinolaryngol **78**(6): 944-949. | 3 |
| Giugliani, R., P. Harmatz and J. E. Wraith (2007). "Management guidelines for mucopolysaccharidosis VI." Pediatrics **120**(2): 405-418. | N/A |
| Simmons, M. A., I. A. Bruce, S. Penney, E. Wraith and M. P. Rothera (2005). "Otorhinolaryngological manifestations of the mucopolysaccharidoses." Int J Pediatr Otorhinolaryngol **69**(5): 589-595. | N/A |
