# Supplementary material for: Recommendations for the management of MPS VI: systematic evidence- and consensus-based guidance
Source: Orphanet J Rare Dis. 2019 May 29;14:118. doi: 10.1186/s13023-019-1080-y (PMC6541999; doi:10.1186/s13023-019-1080-y)
Supplement: Supplementary file 4 — Modified-Delphi voting Round 1. Full results of the first round of the modified-Delphi voting, which was used to demonstrate consensus of the guidance statements. (DOCX 151 kb) [file 13023_2019_1080_MOESM4_ESM.docx]

## Additional File 4: modified-Delphi voting Round 1 results

##

A total of 103 survey submissions were received from 82 individual hospitals/institutions across 20 countries, this included 20 responses from members of the Steering Committee. Following the analysis of the results, 7 respondents did not meet the minimum experience threshold and their submissions were excluded, therefore giving a total of 96 respondents to round 1 of the modified-Delphi survey.

Table 1. Summary of respondents to Round 1 of modified-Delphi voting by specialism, following exclusion of submissions that did not meet the minimum experience threshold

| **List of specialisms** | **Number of respondents** |
| --- | --- |
| Anesthetist | 16 |
| Bone marrow transplant expert/Hematopoietic stem cell transplant expert | 3 |
| Cardiologist | 5 |
| Ear-nose-throat specialist | 5 |
| Geneticist | 8 |
| Hand surgeon | 1 |
| Neurosurgeon | 3 |
| Ophthalmologist | 5 |
| Orthopedic surgeon | 11 |
| Pediatrician | 10 |
| Pulmonologist/Respiratory physician | 5 |
| Other: Adult metabolic consultant | 1 |
| Other: Clinical geneticist and metabolic physician | 1 |
| Other: Clinical nurse specialist | 1 |
| Other: Duel geneticist and pediatrician | 1 |
| Other: Genetic counsellor | 1 |
| Other: Genetic counsellor/MPS Society advocacy support worker | 1 |
| Other: Hematologist/LSD | 1 |
| Other: Internist | 1 |
| Other: Metabolic pediatrician | 1 |
| Other: Metabolic physician | 1 |
| Other: Neuropediatrician | 1 |
| Other: Neuroradiologist | 1 |
| Other: Nurse | 1 |
| Other: Nurse practitioner | 1 |
| Other: Pediatric and adult cardiac surgeon | 1 |
| Other: Pediatric biochemical geneticist | 1 |
| Other: Pediatric metabolic medicine | 1 |
| Other: Pediatric neuropsychologist | 1 |
| Other: Pediatric nurse practitioner | 1 |
| Other: Pediatric rehabilitation specialist | 1 |
| Other: Physiotherapist | 1 |
| Other: Radiologist | 1 |
| Other: Sleep medicine | 1 |
| Other: Speech therapist | 1 |
| **Total** | **96** |

Table 2. Summary of respondents to Round 1 of modified-Delphi voting by country, following exclusion of submissions that did not meet the minimum experience threshold

| **List of countries** | **Number of respondents** |
| --- | --- |
| Argentina | 1 |
| Australia | 5 |
| Austria | 1 |
| Brazil | 5 |
| Canada | 10 |
| Colombia | 5 |
| Czech Republic | 1 |
| Germany | 3 |
| Italy | 6 |
| Japan | 1 |
| Netherlands | 2 |
| New Zealand | 1 |
| Northern Ireland | 1 |
| Poland | 1 |
| Russia | 1 |
| Spain | 1 |
| Sweden | 1 |
| Turkey | 2 |
| UK | 25 |
| USA | 23 |
| **Total** | **96** |

Table 3. modified-Delph voting results for General principles for the management of MPS IVA/VI

| **Statement** | **Number of respondents** | **Percentage consensus** | **Consensus achieved (yes/no)** |
| --- | --- | --- | --- |
| General principles for the management of MPS IVA/VI | | | |
| Diagnosis of MPS IVA/VI during infancy is critical to optimize patient outcomes | 83 | 98% | Yes |
| The first consultation should be conducted by a physician with experience of treating MPS as soon as possible after diagnosis. This should include a full discussion regarding the disease pathology, progression, treatment options and management. Ongoing information should be provided to optimize patient outcomes | 87 | 97% | Yes |
| Patients and caregivers should receive ongoing psychosocial support from a social worker and/or psychologist, and should be directed towards the MPS society or relevant patient organization in their country | 86 | 94% | Yes |
| A comprehensive medical history and multi-system evaluation should be conducted within days of diagnosis to set a baseline for ongoing assessments and evaluate the physical and neurological manifestations of disease, functional ability and disease burden | 84 | 88% | Yes |
| Ongoing and regular, multi-system monitoring, and assessments are recommended to track the natural history of MPS IVA/VI, monitor the impact of treatment and assess the need for treatment interventions to manage the symptoms of MPS IVA/VI. These should be conducted at every clinic visit, annually or in some cases as clinically indicated (for example pre-and post-operatively) | 91 | 100% | Yes |
| Timely interventions are recommended where clinically indicated by monitoring, to help avoid irreversible damage caused by the natural history of MPS IVA/VI, and to manage the disease manifestations and maintain long-term quality of life | 92 | 99% | Yes |
| A multidisciplinary team (MDT) of metabolic specialists, surgeons and allied healthcare professionals (including but not limited to: nurses, physiotherapists, occupational therapists, psychologists and audiologists) is required to manage the diverse range of disease manifestations of MPS IVA/VI | 92 | 99% | Yes |
| Coordination of the entire MDT care team is required prior to any procedure to determine the need for surgery, to discuss the benefits and risks of combining surgeries to minimize the need for multiple anesthesia and to decide the optimal order of procedures. Combination of surgeries should take into consideration the surgical and intubation time, and complexity of procedures | 91 | 93% | Yes |
| The risks and benefits of any intervention and competing risks of other medical problems should be assessed and discussed with patients, families and caregivers to make an informed decision on the appropriateness of the therapy/surgery | 93 | 100% | Yes |
| Surgical procedures should be performed by (or under the guidance of) specialist surgeons and anesthetists with experience of MPS, in medical centers with intensive care units | 92 | 99% | Yes |

Table 4. modified-Delph voting results for recommended routine monitoring and assessments in MPS IVA/VI

| **Statement** | **Number of respondents** | **Percentage consensus** | **Consensus achieved (yes/no)** |
| --- | --- | --- | --- |
| Physical examination | | | |
| A physical examination should be performed during every visit for MPS IVA/VI patients to assess general health, growth, vital signs, abdominal organ size, presence of hernia, neurologic function (including gait), ligamentous laxity, and functions of the eyes, ears, heart and lungs | 41 | 90% | Yes |
| Routine physical examination for MPS IVA/VI patients can also identify signs of potential respiratory problems, such as an enlarged tongue or sniff position | 41 | 90% | Yes |
| Radiology | | | |
| While X-rays are essential to identify the natural history of disease and response to treatment, efforts should be made to minimize radiation exposure throughout the patient’s lifetime, and images should be requested only when clinically useful | 40 | 85% | Yes |
| An anteroposterior (AP) pelvis radiograph should be performed at diagnosis and as clinically indicated (based on physical examination or reports of pain) for MPS IVA/VI patients to quantify hip dysplasia, or to identify early signs of hip migration | 34 | 88% | Yes |
| In MPS IVA/VI patients with clinical evidence of valgus deformity of the lower limbs, standing AP radiographs of lower extremities should be performed prior to guided growth surgery | 30 | 100% | Yes |
| Plain radiography of cervical and thoracolumbar spine is recommended at diagnosis and then every 2–3 years in MPS IVA/VI patients | 38 | 74% | No |
| Magnetic resonance imaging (MRI) of the whole spine (in neutral position) should be performed annually in MPS IVA/VI children to assess for spinal cord injury. The frequency may be reduced for stable adult patients that do not display symptoms | 37 | 84% | Yes |
| Flexion/extension MRI of cervical spine in MPS IVA/VI patients may be needed to identify changes in spinal canal and spinal cord | 35 | 86% | Yes |
| MRI of the brain is recommended at diagnosis in MPS IVA/VI patients to assess for hydrocephalus, with follow up every 2–3 years | 32 | 66% | No |
| MRI of the brain and spinal cord in MPS IVA/VI patients may require sedation or general anesthesia, depending on patient age and cooperation. General anesthesia carries substantial risk for MPS patients | 37 | 95% | Yes |
| Computerized tomography (CT) of neutral region of interest may be considered in MPS IVA/VI patients if MRI is not available or if sedation is not possible | 36 | 69% | No |
| The presence of specific radiological signs in MPS IVA/VI patients may indicate the need for surgical intervention to correct skeletal deformities; however, there is insufficient evidence to support preventative surgery based on radiological findings | 34 | 88% | Yes |
| Endurance | | | |
| Choice of assessment depends on MPS IVA/VI patient’s physical and developmental abilities | 38 | 97% | Yes |
| Baseline assessment is the most important and ideally two values should be obtained as a minimum. Consistent protocols should be used when performing repeat measurements to minimize variability | 39 | 95% | Yes |
| Annual endurance testing using the 6-minute walk test (6MWT) for MPS IVA/VI patients is recommended, as per the American Thoracic Society guidelines | 39 | 87% | Yes |
| In MPS IVA/VI patients with limited ambulation who are unable to do the 6MWT, endurance should be assessed via alternative methods such as an adapted timed 25-foot walk test (T25FW) | 37 | 76% | Yes |
| Endurance testing in MPS IVA/VI patients is also recommended prior to initiation of ERT and annually thereafter as a measure of treatment efficacy and to provide early evidence of possible neurologic or skeletal issues | 38 | 87% | Yes |
| Growth | | | |
| Assessment of growth for MPS IVA/VI patients should be performed at each clinic visit (ideally every 6 months) as part of a regular physical examination and should include: standing height (sitting height if the patient is unable to stand), length (supine position), weight, head circumference (≤3 years), Tanner pubertal stage (until maturity) | 40 | 95% | Yes |
| Height and weight of MPS IVA/VI patients should also be measured before initiation of ERT and at every clinic visit thereafter (ideally every 6 months) to evaluate the impact of treatment | 39 | 95% | Yes |
| Urinary keratan sulphate (KS)/glycosaminoglycan (uGAG) levels | | | |
| Where available tandem mass spectrometry may be used to assess levels of urinary KS prior to starting elosulfase alfa and every 6 months thereafter to determine the pharmacodynamic effects of ERT treatment in MPS IVA patients | 32 | 94% | Yes |
| Total uGAG levels are often elevated in neonates and infants with MPS IVA, and may overlap with normal values in adults and some teenagers. However, if a specific KS assay is not available, measurement of uGAG levels using standard dye-binding methods may be useful. Preferably, measurements should be performed in the same laboratory and assessed against age-related reference values | 33 | 85% | Yes |
| Urinary glycosaminoglycan (uGAG) level | | | |
| Urinary GAG levels should be tested prior to starting galsulfase and every 6 months thereafter to determine the pharmacodynamic effects of ERT in MPS VI patients | 35 | 97% | Yes |
| Measurement of total uGAG levels in MPS VI patients may be performed using standard dye-based quantitative methods, preferably in the same laboratory and assessed against age-related reference values | 30 | 93% | Yes |
| Where available tandem mass spectrometry may be used to assess levels of specific GAGs (such as dermatan sulfate [DS]) in MPS VI patients | 33 | 97% | Yes |
| Cardiac function | | | |
| Initial cardiac evaluation should be performed at the time of diagnosis in MPS IVA/VI patients and include assessment of vital signs with measurement of oxygen saturation, right arm and leg blood pressure measurements, careful auscultation, full transthoracic two-dimensional and Doppler echocardiogram, and 12-lead electrocardiogram (ECG) | 26 | 100% | Yes |
| Longer ECG monitoring (prolonged Holter/Event monitoring) may be considered in older MPS IVA/VI patients especially if they have symptoms of black outs, unexpected falls and dizziness | 24 | 96% | Yes |
| Follow-up in expert centers should be annually initially but may be extended to every 2–3 years if there is no evidence of cardiac abnormality in MPS IVA/VI patients | 26 | 92% | Yes |
| Additional cardiac assessment, including a standard ECG, should be performed prior to any surgical procedures requiring general anesthesia in MPS IVA/VI patients | 25 | 92% | Yes |
| Neurological exam | | | |
| A detailed neurological examination should be performed in MPS IVA/VI patients at every clinic visit (minimally every 6 months) and, where possible, these should correlate with imaging studies of the spine to detect early spinal stenosis or instability compromising the cervical cord. For patients without clinical or radiographic concern, annual neurological examination may be sufficient | 38 | 87% | Yes |
| Flexion/extension cervical spine MRI should be considered for all MPS IVA/VI children with an abnormal neurological examination result | 35 | 74% | No |
| Upper limb function | | | |
| Symptoms of carpal tunnel syndrome (CTS) are often atypical in patients with MPS VI, therefore recommend clinical examination, assessment of range of finger movement and strength, electrophysiology nerve conduction assessment and detailed medical history to be performed at diagnosis and annually thereafter | 27 | 89% | Yes |
| Reach-out tests or the Pediatric Orthopedic Society of North America (POSNA) Pediatric Musculoskeletal Functional Health Questionnaire may also be used to assess hand and upper limb function in MPS VI patients | 18 | 72% | No |
| Respiratory function and sleep disorder | | | |
| Evaluation of respiratory function by spirometry, including forced vital capacity (FVC) and maximum voluntary ventilation (MVV), should be performed to assess changes in lung volume and obstruction on MPS IVA/VI children over 5 years of age | 36 | 97% | Yes |
| Respiratory function should be assessed annually until MPS IVA/VI children stop growing, and every 2–3 years thereafter provided that respiratory symptoms remain unchanged. Additional testing should be performed if respiratory symptoms change or if intercurrent illnesses occur | 35 | 91% | Yes |
| Normative values are not available, therefore change in absolute volume from MPS IVA/VI patients own baseline will be the best indicator of deterioration or improvement | 35 | 97% | Yes |
| Measuring respiratory rate and arterial oxygen saturation before and after annual endurance testing is recommended in MPS IVA/VI patients | 29 | 86% | Yes |
| Evaluation of gas exchange and respiratory function is also recommended before any planned air travel, to ensure safety during the flight in MPS IVA/VI patients | 29 | 86% | Yes |
| MPS IVA/VI patients should be asked to report presence of snoring and morning headaches to identify symptoms of sleep apnea at every clinic visit | 38 | 100% | Yes |
| Overnight sleep study (polysomnography) is recommended at diagnosis (if possible, and no later than 2 years of age), and every 3 years thereafter or when signs and symptoms of obstructive sleep apnea (OSA) are noted in MPS IVA/VI patients | 35 | 94% | Yes |
| Ear-nose-throat (ENT) | | | |
| ENT examination, including tympanometry, should be conducted every 3–6 months during childhood and every 6–12 months thereafter in MPS IVA/VI patients | 23 | 91% | Yes |
| Each ENT examination in MPS IVA/VI patients should include a recorded flexible nasopharyngolaryngoscopy to visualize the upper respiratory tract. If airway obstruction involving a site other than the upper respiratory tract is suspected, rigid endoscopic evaluation under general anesthesia is indicated to assess the whole airway | 26 | 69% | No |
| Age-adjusted audiometric assessment as a baseline objective hearing evaluation should be conducted at first clinic visit and repeated annually to assess conductive and sensory-neural hearing loss in MPS IVA/VI patients | 25 | 100% | Yes |
| If speech problems are determined during the ENT examination, an assessment by a speech pathologist should be conducted in MPS IVA/VI patients | 23 | 100% | Yes |
| Balance tests should be conducted if the MPS IVA/VI patient has a history of balance problems | 20 | 95% | Yes |
| Ophthalmological function | | | |
| Age-appropriate evaluations by an ophthalmologist to assess ophthalmic function is recommended for MPS IVA/VI patients every 6 months if possible, or at least annually | 21 | 90% | Yes |
| Ophthalmic assessment for MPS IVA/VI patients may include visual acuity, refraction, slit-lamp examination of cornea, funduscopic evaluation including optic nerve, and measurement of intraocular pressure | 19 | 100% | Yes |
| Scotopic and photopic electroretinogram may be performed in MPS IVA patients with clinical suspicion of retinopathy or when considering corneal transplantation | 13 | 100% | Yes |
| Intraocular pressure monitoring and pachymetry may be considered prior to corneal transplant in MPS IVA/VI patients | 15 | 100% | Yes |
| Evaluation of oral health by dentist | | | |
| Close monitoring of dental development (at least annually) is recommended in MPS IVA/VI patients to prevent caries and attrition of the teeth, and monitoring of occlusion and chewing functions | 28 | 100% | Yes |
| The need for subacute bacterial endocarditis (SBE) prophylaxis prior to dental procedures in MPS IVA/VI patients should be assessed by a cardiologist | 26 | 100% | Yes |
| Disease burden | | | |
| Annual assessment of patient-reported outcomes is recommended for: pain severity, quality of life (QoL) as assessed by reproducible and age-appropriate questionnaires (eg EQ-5D-5L), fatigue, and activities of daily living (ADL) as assessed by functional tests (6MWT/T25FW), age-appropriate ADL questionnaires (eg MPS Health Assessment Questionnaire [MPS HAQ]), and assessment of wheelchair/walking aid use | 39 | 97% | Yes |
| These assessments may have to be adapted both for language, culture and individual physical limitations as they have not been validated in these specific disorders | 39 | 97% | Yes |
| Physical therapy | | | |
| Regular assessments should be conducted for MPS IVA/VI patients by a physical therapist (lower limb), occupational therapist (upper limb) and rehabilitation medicine specialist to assess upper and lower function and provide support as needed | 40 | 93% | Yes |
| Physical therapists could also assist in suggesting walking aids and other adaptations that may improve QoL for MPS IVA/VI patients | 40 | 98% | Yes |

Table 5. modified-Delph voting results for disease-modifying interventions

| **Statement** | **Number of respondents** | **Percentage consensus** | **Consensus achieved (yes/no)** |
| --- | --- | --- | --- |
| Enzyme replacement therapy (elosulfase alfa) in MPS IVA | | | |
| Initiation of life-long ERT with elosulfase alfa at a dose of 2 mg/kg/week through intravenous infusion is recommended in all MPS IVA patients as soon as possible after a confirmed diagnosis | 38 | 79% | Yes |
| Enzyme replacement therapy (galsulfase) in MPS VI | | | |
| Initiation of life-long ERT with galsulfase at a dose of 1 mg/kg/week through intravenous infusion is recommended in all MPS VI patients as soon as possible after a confirmed diagnosis | 39 | 74% | No |
| Hematopoietic stem cell transplantation in MPS IVA/VI | | | |
| HSCT should only be considered at diagnosis in exceptional circumstances for young, clinically stable MPS IVA patients who have matched related (non-carrier) donor, or well-matched unrelated donor or cord blood graft | 34 | 62% | No |
| HSCT may be an option at diagnosis for young, clinically stable MPS VI patients who have a matched related (non-carrier) donor, or well-matched unrelated donor or cord blood graft | 36 | 69% | No |
| For MPS IVA/VI patients, HSCT should be performed in an institution with a MDT experienced in the care of individuals with MPS and established Institutional Review Board (IRB)-approved protocols | 38 | 84% | Yes |
| HSCT may also be an option for MPS IVA/VI patients who do not tolerate, or cannot access, ERT (for example patients who experience severe adverse events leading to ERT discontinuation) and who meet the above criteria | 35 | 83% | Yes |

Table 6. modified-Delph voting results for anesthetics and surgical interventions

| **Statement** | **Number of respondents** | **Percentage consensus** | **Consensus achieved (yes/no)** |
| --- | --- | --- | --- |
| Anesthetics in MPS IVA/VI | | | |
| Pre-, intra- and post-operative care (until extubation is complete) for all procedures requiring general anesthesia, or conscious or deep sedation, should be supervised by an anesthetist with experience in MPS and/or complex airway management. In addition, the anesthetist should have access to Intensive Care support and be surrounded by an experienced team capable of performing emergency tracheotomy if required | 42 | 98% | Yes |
| A full assessment of the risks and benefits should take place with the patient and family prior to any procedure. All pre-operative information should be made available to allow decision making | 43 | 100% | Yes |
| ENT, respiratory, cardiac, and radiological assessment should be performed prior to any procedure requiring anesthesia | 42 | 93% | Yes |
| It is critical to maintain a neutral neck position during all surgeries, and during intubation and extubation to avoid paralysis. Strongly recommend the use of techniques that allow maintenance of the neutral neck position, including use of laryngeal mask airway (LMA) for shorter procedures, or intubation with a video laryngoscope or fiberoptic intubation | 39 | 87% | Yes |
| Pre-operative and intra-operative measures to avoid hypotension should be adopted during all surgical procedures in patients with MPS IVA/VI to maintain spinal cord perfusion and therefore protect spinal cord function | 40 | 98% | Yes |
| Intra-operative neurophysiological monitoring (including somatosensory evoked potentials [SSEP], electromyography [EMG] and motor evoked potentials [MEP]) is strongly recommended during all spinal surgeries and other potentially lengthy or complicated procedures, including those that require manipulation of the head and neck | 34 | 94% | Yes |
| For other surgeries and procedures, neurophysiologic monitoring should be considered based on pre-existing risk for spinal cord compression and instability, need for spine manipulation, possibility of hemodynamic changes and blood loss, or extended length of time | 36 | 94% | Yes |
| Intrathecal and epidural techniques should be used with extreme caution in MPS VI, due to the anatomical challenges of very short stature, as well as spinal abnormalities causing insertion problems and unpredictability of spread of local anesthesia. However, these techniques may be considered to avoid general anesthesia in a high-risk situation or during pregnancy | 32 | 88% | Yes |
| Intrathecal and epidural techniques are high-risk in patients with MPS IVA and should be avoided wherever possible | 29 | 83% | Yes |
| Limb Surgeries in MPS IVA | | | |
| Hip reconstruction can be considered in pediatric MPS IVA patients who exhibit hip pain, reduced walking and endurance related to hip disease, as well as abnormal radiographic findings | 29 | 86% | Yes |
| Hip replacement can be considered in adult MPS IVA patients who exhibit hip pain, reduced walking and endurance related to hip disease, as well as abnormal radiographic findings | 26 | 100% | Yes |
| Growth modulation is recommended in all MPS IVA patients who have evidence of genu valgum and should be performed as early as possible during the period of growth | 22 | 77% | Yes |
| Limb Surgeries in MPS VI | | | |
| Hip replacement can be considered in adult MPS VI patients who exhibit hip pain, reduced walking and endurance related to hip disease, as well as abnormal radiographic findings | 28 | 100% | Yes |
| Hip reconstruction is not routinely indicated but may be considered in pediatric MPS VI patients who exhibit hip pain, reduced walking and endurance related to hip disease, as well as abnormal radiographic findings | 24 | 92% | Yes |
| Growth modulation is recommended in MPS VI patients who have signs of genu valgum and should be performed as early as possible during the period of growth | 23 | 87% | Yes |
| Spinal surgeries in MPS IVA | | | |
| Decompression of the spinal cord is recommended in MPS IVA patients who have evidence of spinal cord compression based on clinical and radiographic findings | 36 | 97% | Yes |
| Spinal stabilization of the craniocervical junction with either cervical fusion or occipital-cervical fusion is recommended in MPS IVA patients who have evidence of instability | 36 | 97% | Yes |
| Correction of thoracolumbar kyphoscoliosis is recommended in MPS IVA patients who present with progressive radiographic deformity, intractable pain and neurological deterioration | 30 | 100% | Yes |
| Spinal surgeries in MPS VI | | | |
| Decompression of the spinal cord is recommended in MPS VI patients who have evidence of spinal cord compression based on clinical and radiographic findings | 35 | 97% | Yes |
| Spinal stabilization of the craniocervical junction with either cervical fusion or occipital-cervical fusion is recommended in MPS VI patients who have evidence of instability | 36 | 100% | Yes |
| Correction of thoracolumbar kyphoscoliosis is recommended in MPS VI patients who present with progressive radiographic changes, intractable pain and clinical deterioration as defined by gait, lung function and changes in the degree of kyphosis | 32 | 97% | Yes |
| Ophthalmic surgery in MPS IVA | | | |
| While significant corneal clouding is rare in MPS IVA patients, corneal transplantation can be considered for patients with significant visual loss attributed to corneal opacification | 19 | 95% | Yes |
| Ophthalmic surgery in MPS VI | | | |
| Corneal transplantation can be considered for MPS VI patients with significant visual loss attributed to corneal opacification | 21 | 100% | Yes |
| Carpal tunnel decompression in MPS VI | | | |
| Decompression of the median nerve and tensosynovectomy of all flexor tendons in the carpal tunnel is recommended in MPS VI patients who display flexion contractures and distal interphalangeal (DIP) joints and/or proximal interphalangeal (PIP) joints (clawing), as well as clinical symptoms of hand pain and/or numbness in the thumb to middle finger, or in patients with positive nerve conduction studies | 28 | 89% | Yes |
| A1 and A3 pulley release is recommended in MPS VI patients who display obvious trigger finger | 18 | 94% | Yes |
| Cardio-thoracic surgery in MPS IVA | | | |
| Cardiac (aortic, mitral) valve replacement should be considered in patients with MPS IVA who display symptomatic and severe valve stenosis or regurgitation | 21 | 95% | Yes |
| Cardio-thoracic surgery in MPS VI | | | |
| Cardiac (aortic, mitral) valve replacement should be considered in patients with MPS VI who display symptomatic and severe valve stenosis or regurgitation | 23 | 100% | Yes |
| Left ventricular apical aneurysms occur rarely in patients with MPS VI but should be resected whenever possible | 13 | 85% | Yes |
| Respiratory interventions and sleep disorders in MPS IVA | | | |
| CPAP therapy is recommended for MPS IVA patients who display the presence of OSA which persists after tonsillectomy and/or adenoidectomy | 36 | 97% | Yes |
| NIPPV therapy is recommended for MPS IVA patients who display nocturnal hypoventilation and are unresponsive to CPAP, or display daytime hypoventilation with increased PaCO2 and/or serum HCO3 levels | 34 | 91% | Yes |
| Oxygen supplementation during sleep is recommended for MPS IVA patients who exhibit sleep apnea with nocturnal hypoxemia, and who do not tolerate CPAP or NIPPV masks | 30 | 77% | Yes |
| MPS IVA patients should be monitored for development of hypercapnia after starting oxygen therapy with measurement of PaCO2 and/or serum HCO3 | 29 | 97% | Yes |
| Respiratory Interventions and Sleep Disorders in MPS VI | | | |
| CPAP is recommended therapy for MPS VI patients who display the presence of OSA which persists after tonsillectomy and/or adenoidectomy | 35 | 100% | Yes |
| NIPPV therapy is recommended for MPS VI who display nocturnal hypoventilation and are unresponsive to CPAP, or display daytime hypoventilation with increased PaCO2 and/or serum HCO3 levels | 34 | 94% | Yes |
| Oxygen supplementation during sleep is recommended for MPS VI patients that display sleep apnea with nocturnal hypoxemia, and who do not tolerate CPAP or NIPPV masks | 30 | 83% | Yes |
| MPS VI patients should be monitored for development of hypercapnia after starting oxygen therapy with measurement of PaCO2 and/or serum HCO3 | 29 | 97% | Yes |
| ENT Surgery in MPS IVA | | | |
| Tonsillectomy and/or adenoidectomy is recommended for MPS IVA patients who display recurrent otitis media, or snoring and/or obstructive sleep apnea (OSA) as early as possible following diagnosis without waiting for disease progression | 32 | 94% | Yes |
| Insertion of ventilation tubes is recommended for MPS IVA patients with otitis media with effusion and/or recurrent otitis media to maintain hearing and/or prevent recurrent acute otitis media | 28 | 100% | Yes |
| Uvulopalatopharyngoplasty and/or mandibular advancement surgeries should be considered in MPS IVA patients who display the presence of OSA which persists after tonsillectomy and/or adenoidectomy | 20 | 55% | No |
| Partial tongue reduction could be considered in MPS IVA patients who display the presence of OSA which persists after tonsillectomy and/or adenoidectomy | 19 | 42% | No |
| Tracheostomy is recommended in MPS IVA patients that do not respond to any of the treatment modalities mentioned above | 30 | 77% | Yes |
| ENT Surgery in MPS VI |  |  |  |
| Tonsillectomy and/or adenoidectomy is recommended in MPS VI patients who display upper airway obstruction, recurrent otitis media, snoring and/or OSA as early as possible following diagnosis, without waiting for disease progression | 33 | 91% | Yes |
| Uvulopalatopharyngoplasty and/or mandibular advancement surgeries should be considered in MPS VI patients, who display the presence of OSA which persists after tonsillectomy and/or adenoidectomy | 20 | 65% | No |
| Partial tongue reduction could be considered in MPS VI patients, who display the presence of OSA which persists after tonsillectomy and/or adenoidectomy | 22 | 64% | No |
| Tracheostomy is recommended in MPS VI patients that exhibit severe upper airway obstruction, which cannot be treated by an alternative approach, or in patients with severe sleep apnea that is not treatable by CPAP or tonsillectomy and/or adenoidectomy | 21 | 95% | Yes |
| Insertion of ventilation tubes is recommended in MPS VI patients with otitis media with effusion and/or recurrent otitis media to maintain hearing and/or prevent recurrent acute otitis media | 28 | 96% | Yes |

Table 7. Summary of the statements that did not reach consensus

| **Statement** | **Number of respondents** | **Percentage consensus** |
| --- | --- | --- |
| **Radiology** | | |
| Plain radiography of cervical and thoracolumbar spine is recommended at diagnosis and then every 2–3 years in MPS IVA/VI patients | 38 | 74% |
| MRI of the brain is recommended at diagnosis in MPS IVA/VI patients to assess for hydrocephalus, with follow up every 2–3 years | 32 | 66% |
| Computerized tomography (CT) of neutral region of interest may be considered in MPS IVA/VI patients if MRI is not available or if sedation is not possible | 36 | 69% |
| **Neurological exam** | | |
| Flexion/extension cervical spine MRI should be considered for all MPS IVA/VI children with an abnormal neurological examination result | 35 | 74% |
| **Upper limb function** | | |
| Reach-out tests or the Pediatric Orthopedic Society of North America (POSNA) Pediatric Musculoskeletal Functional Health Questionnaire may also be used to assess hand and upper limb function in MPS VI patients | 18 | 72% |
| **Ear-nose-throat (ENT)** | | |
| Each ENT examination in MPS IVA/VI patients should include a recorded flexible nasopharyngolaryngoscopy to visualize the upper respiratory tract. If airway obstruction involving a site other than the upper respiratory tract is suspected, rigid endoscopic evaluation under general anesthesia is indicated to assess the whole airway | 26 | 69% |
| **Enzyme replacement therapy** | | |
| Initiation of life-long ERT with galsulfase at a dose of 1 mg/kg/week through intravenous infusion is recommended in all MPS VI patients as soon as possible after a confirmed diagnosis | 39 | 74 |
| **Haematopoietic Stem Cell Transplantation** | | |
| HSCT should only be considered at diagnosis in exceptional circumstances for young, clinically stable MPS IVA patients who have matched related (non-carrier) donor, or well-matched unrelated donor or cord blood graft | 34 | 62 |
| HSCT may be an option at diagnosis for young, clinically stable MPS VI patients who have a matched related (non-carrier) donor, or well-matched unrelated donor or cord blood graft | 36 | 69 |
| **ENT Surgery in MPS IVA** | | |
| Uvulopalatopharyngoplasty and/or mandibular advancement surgeries should be considered in MPS IVA patients who display the presence of OSA which persists after tonsillectomy and/or adenoidectomy | 20 | 55 |
| Partial tongue reduction could be considered in MPS IVA patients who display the presence of OSA which persists after tonsillectomy and/or adenoidectomy | 19 | 42 |
| **ENT Surgery in MPS VI** | | |
| Uvulopalatopharyngoplasty and/or mandibular advancement surgeries should be considered in MPS VI patients, who display the presence of OSA which persists after tonsillectomy and/or adenoidectomy | 20 | 65 |
| Partial tongue reduction could be considered in MPS VI patients, who display the presence of OSA which persists after tonsillectomy and/or adenoidectomy | 22 | 64 |

Respondent feedback for each Key Action Statement

*General principles for management*

| **Statement** | | **Consensus achieved (yes/no) (%)** |
| --- | --- | --- |
| **Diagnosis of MPS IVA/VI during infancy is critical to optimize patient outcomes** | | Yes (98) |
| **Comments** | | |
| *If you disagree with the statement, please explain why and suggest an amendment* | - Certainly 'the earlier you treat' (with ERT) the 'better' the outcome, but outcomes with ERT for MPS IVA and VI especially with limited access of enzyme to joint tissues are still poor. What are you going to accomplish with earlier treatment? Certainly, does not forestall need for orthopedic or ENT surgeries as bony structures remain severely dysplastic. - For severe patients yes. For very attenuated patient’s diagnosis and treatment in early infancy may not necessarily be appropriate | |
| *Additional comments or suggestions* | - Best outcomes are with early diagnosis - Early ERT may be of benefit - How are we defining infancy here? Up to 6 months? 12 months? I don't think the majority of patients are diagnosed in the first year. The earlier the better makes sense. I'm not sure there is enough data to say diagnosis at 6 months leads to a better outcome than a diagnosis at 18 months, though - Local experience has clearly demonstrated better outcomes occur when diagnosed and treated from infancy - For MPS VI, HSCT may be discussed with parents of children with MPS VI - It should be made as soon as manifestations are evident. Enzyme replacement should begin as soon as possible - I would support new-born screening for these disorders - Early diagnosis leads to early treatment and therefore to limited effects of the storage disorder - I am not a physician, but it is always sensible/optimal to diagnose congenital conditions as soon as it is realistically possible to do so. Even if treatment is not available, the condition should be known about early - Some slowly progressive patients may not necessarily benefit from diagnosis in infancy - The early diagnosis in relation to the natural course of the disease | |
| **Statement** | | **Consensus achieved (yes/no) (%)** |

| **The first consultation should be conducted by a physician with experience of treating MPS as soon as possible after diagnosis. This should include a full discussion regarding the disease pathology, progression, treatment options and management. Ongoing information should be provided to optimize patient outcomes** | | Yes (97) |
| --- | --- | --- |
| **Comments** | | |
| *If you disagree with the statement, please explain why and suggest an amendment* | - Requirement for information about genetics and inheritance to be discussed at an early stage. This would support families to make decisions about their reproductive options - Early assessment of children is done by non-expert personnel in metabolism errors in most countries. I agree to give staff the biggest tool for their clinical diagnosis and in many cases their follow up to optimize patient outcome. The reference centers for patients are not abundant and are not close | |
| *Additional comments or suggestions* | - Otherwise misinformation habitually ensues, leading to unnecessary angst - The physician should be able to provide any information on the full spectrum (pathology – management) relevant at this early time point and should answer respective questions of parents/patients. Yet in most patients/parents will only be able to digest a small amount of information. Thus, it is very important, that an experienced physician selects the most relevant information for the individual patient/family - I agree that the first consultation should be conducted by a physician with appropriate experience as soon after diagnosis as possible - but I think the full discussion regarding pathology, progression, treatment and management would take place over the first few meetings. Otherwise it would be a huge amount for parents to absorb on top of bad news - Repeated consultations are of importance similarly to other severe disorders - Contact to national society for MPS is of importance too - It can be disastrous if parents are given inaccurate information about the condition particularly if they are not given information about treatment options - Early diagnosis leads to early treatment and therefore to limited effects of the storage disorder - The first consultation following diagnosis should be conducted by a physician with knowledge, so they could be seen by an interested local pediatrician who has discussed the case with an expert and/or had guidelines sent to them about what to say and what will happen and then the referral can be actioned - The first consultation following diagnosis should be conducted by a physician with knowledge, so they could be seen by an interested local pediatrician who has discussed the case with an expert and/or had guidelines sent to them about what to say and what will happen and then the referral can be actioned | |

| **Statement** | | **Consensus achieved (yes/no) (%)** |
| --- | --- | --- |
| **Patients and caregivers should receive ongoing psychosocial support from a social worker and/or psychologist, and should be directed towards the MPS society or relevant patient organization in their country** | | Yes (94) |
| **Comments** | | |
| *If you disagree with the statement, please explain why and suggest an amendment below:* | - While I do believe that social support is important, and that families should be introduced to their relevant family organization, formal social work referrals and psychology evaluation are probably not necessary for all families or caregivers - This is very important but not in the first consultation and not for all cases. Some cases (patients) do not accept the psychological support - Strongly agree that families (parents, guardians, siblings and affected patients require regular and ongoing support from a social worker and/or psychologist, but this support can be provided from any support health worker rather than just the MPS Societies. This may be dependent on funding sources. Ideally the health support worker should have knowledge and experience in working with individuals/families with MPS - Would delete the "from a social worker and/or psychologist" - They should be offered the support, but they should be empowered to remain in charge of the situation (so as much support as they want and as limited disturbance as the wish) | |
| *Additional comments or suggestions (Optional):* | - This is a very traumatic time in a family's life - Genetic counselors are other good options - A chronic disease diagnosis with multisystem involvement is going to be very challenging for a family to cope with. Psychosocial support is a must-have. - This is in an ideal world - If needed/wanted. Should not be pushed onto them - I agree to offer professional psychosocial support. As far as "directing" them to the MPS society or patient organization. They need to be made aware of such groups but be left with them choosing to contact or not - Information about the MPS Society should be passed on to families to consider. The involvement of the MPS Society at specialist clinics should be facilitated - The right to privacy must prevail. Parents and adult patients must decide what the best source of support is for them - My only reservation here would be what is the defined role of the MPS Society? Should this be expanded upon? Is it education, interventional opportunities/clinical trials? Support? Connection with other families with similar issues? All of the above? | |

| **Statement** | | **Consensus achieved (yes/no) (%)** |
| --- | --- | --- |
| **A comprehensive medical history and multi-system evaluation should be conducted within days of diagnosis to set a baseline for ongoing assessments and evaluate the physical and neurological manifestations of disease, functional ability and disease burden** | | Yes (88) |
| **Comments** | | |
| *If you disagree with the statement, please explain why and suggest an amendment below:* | - Multi-system evaluation is very important, but not so important that it should be done within days - "Within days" is too specific. Would prefer “as soon as possible" - It is not oncology, the importance within days is speculative and depends very much on the individual family and education - I certainly agree comprehensive medical history and evaluation should be done. I disagree with the statement 'within days of diagnosis.' The evaluation does not need to be performed with that degree of urgency, especially as the disease progression is slow - Completing age appropriate baseline assessments within days of diagnosis is not realistic at my institution. There may not be appointments available for a few weeks or more. Suggestion would be to change "within days" to as soon as possible. Also, there is less urgency in MPS IVA and MPS VI compared to other MPSs as these patients do not have cognitive regression - This is not urgent and can be done within a few weeks. Days is of course better for the family who will be stunned by this diagnosis, but medically it is impractical to get this evaluation done by knowledgeable individuals within days - This should certainly be conducted but I disagree with "within days of diagnosis". For many families the diagnosis will be a huge blow and some time to come to terms with the diagnosis whilst ongoing assessments are arranged gradually over days/weeks is not necessarily a bad thing - At some stage but not necessarily within days of diagnosis - Within days of diagnosis isn’t that necessary as the disease is not that fast in progressing, however a full multisystem evaluation should be made within a number of weeks/months to optimize management | |
| *Additional comments or suggestions (Optional):* | - "Days" should be more specific - As soon as possible. If we believe this consult should be done by an experienced physician, then within days, may not be practical - Change "within days" to "as soon as possible" - It is important here to detect life threatening disease complications as well as quality of life reducing disease manifestations - Really important. I think we now realize we should have collected much more natural history data about MPS conditions - There is no doubt that a baseline/intake evaluation is needed, I wonder about the timing "within days" or rather "within the first month" - I think the wording "within days of diagnosis" sets a potentially unrealistic expectation for this evaluation to occur for some patients. If the patient does not live close to a medical provider or if a provider is not available who can perform a "comprehensive" evaluation, it may take longer than this to be evaluated. Maybe "as soon as possible" or "at the soonest possible opportunity" etc. - Depends on definition of "evaluation." If evaluation means "physical examination" then I would change the wording to "physical examination" - 'Within days' is perhaps a bit ambitious and may be overwhelming. it would be important to set up a schedule of assessments as soon as possible - Strongly agree - Any opportunity for biorepositories to collect samples prior to intervention? - I agree with the overall statement, but I would disagree with having to do it "within days" | |

| **Statement** | | **Consensus achieved (yes/no) (%)** |
| --- | --- | --- |
| **Ongoing and regular, multi-system monitoring, and assessments are recommended to track the natural history of MPS IVA/VI, monitor the impact of treatment and assess the need for treatment interventions to manage the symptoms of MPS IVA/VI. These should be conducted at every clinic visit, annually or in some cases as clinically indicated (for example pre-and post-operatively)** | | Yes (100) |
| **Comments** | | |
| *If you disagree with the statement, please explain why and suggest an amendment below:* |  | |
| *Additional comments or suggestions (Optional):* | - The visit need to be performed every 4 months - As above - It is mandatory to maintain the balance between the needed check-ups and the burden for the patient and the family. In this case the check-ups should be as much as possible clustered to limit the time of the patient in hospitals - I like the idea of guidelines but not rules about this unless it’s going to be a very integrated and efficient MDT clinic. It just means a lot of (largely unnecessary) clinic appointments - Too vague on detail e.g. which assessments need to be done every clinic visit, how often should clinic visits be, which tests need to be done annually etc - Sometimes the availability of this monitoring might be affected by the geographical location of the patients and their proximity with the reference center - What does "track the natural history" mean? Are there specific questions and/or data that should be collected at these visits? If so, is that information provided? - I think that "every six months, whenever possible" would be better than "annually" | |

| **Statement** | | **Consensus achieved (yes/no) (%)** |
| --- | --- | --- |
| **Timely interventions are recommended where clinically indicated by monitoring, to help avoid irreversible damage caused by the natural history of MPS IVA/VI, and to manage the disease manifestations and maintain long-term quality of life** | | Yes (99) |
| **Comments** | | |
| *If you disagree with the statement, please explain why and suggest an amendment below:* | - Some interventions can be precise without real benefit for patient | |
| *Additional comments or suggestions (Optional):* | - As it relates to hand surgery, timely diagnosis and management of carpal tunnel syndrome in MPS prevents irreversible muscle atrophy and dysfunction in the hand - This should include the option of entrance into relevant clinical trials | |

| **Statement** | | **Consensus achieved (yes/no) (%)** |
| --- | --- | --- |
| **A multidisciplinary team (MDT) of metabolic specialists, surgeons and allied healthcare professionals (including but not limited to: nurses, physiotherapists, occupational therapists, psychologists and audiologists) is required to manage the diverse range of disease manifestations of MPS IVA/VI** | | Yes (99) |
| **Comments** | | |
| *If you disagree with the statement, please explain why and suggest an amendment below:* | - I do not think everyone has to be there overtime. We have to try and maintain a holistic approach to the child and to doctoring. A metabolic specialist should be able to tell me whether the knees are straight or not and do the x-ray that I might want to review but the patient may not need to see me specifically every time. The child's care should not be chopped up into tiny bits so that everyone can look at their little bit - I remain worried that in such situations no-one is interested/capable of putting all the little bits together in a sensible/logical way that then is a true representation of the child themselves and not another picture entirely | |
| *Additional comments or suggestions (Optional):* | - Agree with the point, but metabolic specialists may be to narrow a classification. I am a geneticist and see a lot of MPS patients, but do not consider myself a metabolic specialist - again, this is nice but not always practical in all areas of the world - The MDT approach is essential as these patients have such complex needs - In any team a central coordinator specialized nurse or specialized secretary seems most imported for this multidisciplinary approach. Because of the limited number of patients, it is advisable, not to spread them over a big number of medical specialists of any specialty - Important with good communication between the MDT and the staff responsible for daily care at rehabilitation centers or at home - This should include the option of entrance into relevant clinical trials - All specialties should be appropriately trained in MPS diseases and should be facilitated to spend time working under specialist centers to develop their knowledge and skills base - That is a pretty partial list. Seems it should be expanded upon, or otherwise not sure it is helpful at all - This MDT should also include neurosurgeons/spinal surgeons/ENT surgeons and Pediatric-Congenital Cardiac surgeons able to deal with these patients in pediatric and adult age. Also, specialist chest physicians/ICU physicians with an interest in complex respiratory patients. Any type of intervention should take place in an environment where complex pediatric or adult surgery can be delivered safely. Clinicians with expertise should be enrolled. Care provided in the right environment for the patient rather than dependent on surgeons is required. Centralized surgical care would be optimal. We had this model when I provided the cardiac surgical care for these patients in Manchester which worked well with good outcomes - It is the only way to manage rare diseases - I would also include primary care providers in this "medical home" concept - A centralized evaluation of neuroimaging by an expert. Neuroradiologists might be useful for a correct interpretation of radiological findings both at diagnosis and at follow-up examinations - The term "required" could be replaced by "beneficial" | |

| **Statement** | | **Consensus achieved (yes/no) (%)** |
| --- | --- | --- |
| **Coordination of the entire MDT care team is required prior to any procedure to determine the need for surgery, to discuss the benefits and risks of combining surgeries to minimize the need for multiple anesthesia and to decide the optimal order of procedures. Combination of surgeries should take into consideration the surgical and intubation time, and complexity of procedures** | | Yes (93) |
| **Comments** | | |
| *If you disagree with the statement, please explain why and suggest an amendment below:* | - I am not convinced that a committee needs to meet for every procedure, but the team should be aware of issues - "Prior to any" seems very restrictive. Perhaps "any major" - Having an MDT is the ideal situation, but not always realistic, and I do not think the entire team has to be involved in every decision. We do try to combine procedures if this can be arranged with the surgeons, radiologists, etc. An anesthesia consult is always recommended - Again, this sounds good in theory, but it is often not actually practical /desirable. Although I do agree it should be discussed. I feel that the parents should not be led to expect that everything can/should be done together. We, as surgeons, rather than they as parents are usually in a better position to say how much it is likely that a kid can cope with - This in practice is very easy to state but virtually impossible to set into practice. Each patient should have at least 1 very knowledgeable physician who oversees their care and ensures that discussions related to optimization of outcome is in place - Not all team members need always to be consulted | |
| *Additional comments or suggestions (Optional):* | - Ideally all MPS IVA/VI patients should have regular anesthetic assessment to identify those patients at high risk of problematic management. In cases where disease progression is slow these assessments may be spaced at long intervals (infant, child, adolescent), in those where disease progression is more rapid annual assessment is more appropriate - If at all possible. I also think it is important that all patients have some form of 'emergency plan' as it is not always possible to get the MDT together in an urgent situation - I would omit "entire" - No surgeries should be undertaken outside of a specialist center unless agreed by the specialist team. It is important that ICU/HDU facilities are available in the location of surgery - Whole allied professional MDT may not be needed on every occasion but certainly the medical professions involved in decision making re: type and number of operations - Not sure the team listed above (nurses, OT, PT, etc.) are required to weigh in for every procedure. A discussion with the metabolic specialist, anesthesiology and surgeon however seems necessary, with requested participation of any others that would be helpful - See above - This is the best practice to manage the patients (not always possible) however grouping surgical procedures is a must to prevent multiple anesthetics which are extremely high-risk events in these patients due to their airways | |

| **Statement** | | **Consensus achieved (yes/no)** |
| --- | --- | --- |
| **The risks and benefits of any intervention and competing risks of other medical problems should be assessed and discussed with patients, families and caregivers to make an informed decision on the appropriateness of the therapy/surgery** | | Yes (100) |
| **Comments** | | |
| *If you disagree with the statement, please explain why and suggest an amendment below:* |  | |
| *Additional comments or suggestions (Optional):* | - Situations may arise where a ceiling of care may need to be agreed between patient, family, physician, surgeon and intensivists - Bearing in mind in acute emergencies where a very urgent intervention is life-saving this may not happen at a level of detail and satisfaction ideally aimed for - I think we do this, as surgeons - Informed consent is the basis of surgeon's practice and I agree it should apply to physicians/orthotists/physios as well. Unnecessary splints can make life a misery | |

| **Statement** | | **Consensus achieved (yes/no)** |
| --- | --- | --- |
| **Surgical procedures should be performed by (or under the guidance of) specialist surgeons and anesthetists with experience of MPS, in medical centers with intensive care units** | | **Yes (99)** |
| **Comments** | | |
| *If you disagree with the statement, please explain why and suggest an amendment below:* | - Depends on the procedure | |
| *Additional comments or suggestions (Optional):* | - These centers should ideally have a team approach to avoid the concentration of expertise in a single individual. Career succession is essential - With special emphasis on anesthesia - I have published literature on the subject supporting this statement - Fully agree for planned procedures but again in emergencies it may not be possible to achieve this. - Anesthetic complications are common in these patients even if they are having minor procedures so full back up including ICU is essential - The term under guidance is key here. An excellent surgeon is an excellent surgeon, I do not believe that surgical procedures themselves are intrinsically different for MPS patients. What is key is advice in relation to the timing of intervention and the appropriate monitoring during procedures and after procedures. | |

Recommended routine monitoring and assessments in MPS IVA/VI

Physical examination

| **Statement** | | **Consensus achieved (yes/no) (%)** |
| --- | --- | --- |
| **A physical examination should be performed during every visit for MPS IVA/VI patients to assess general health, growth, vital signs, abdominal organ size, presence of hernia, neurologic function (including gait), ligamentous laxity, and functions of the eyes, ears, heart and lungs** | | Yes (90) |
| **Comments** | | |
| *If you disagree with the statement, please explain why and suggest an amendment below:* | - We do not do this on every occasion that patients attend for ERT but do this when they attend clinic. A physical examination should be performed during every clinic visit for MPS IVA/VI patients to assess general health, growth, vital signs, abdominal organ size, presence of hernia, neurologic function (including gait), ligamentous laxity, and functions of the eyes, ears, heart and lungs - No, if you are seeing someone weekly for ERT, this is not necessary. A complete exam should be done at routine intervals, but every visit, may be excessive - We see patients weekly at the time of infusion, this statement implies that a full PE be completed at each visit. Need to distinguish--give some time from such as every 6 months - This is not feasible if the patient is attending weekly for ERT but should be performed at all clinic visits | |
| *Additional comments or suggestions (Optional):* | - Symptoms are different in children with MPS IV and VI, recommendation for follow cannot be the same - Good medical practice! - Define "every visit". Does it mean visiting the MDT team or general all visits in healthcare? Perhaps better with a minimum number of visits that require a physical examination of the patient's status - I think the above should be on a timetable - for instance all the above every 6 months, or a year. PE is easy enough, but pulmonary function testing, exams by ophthalmology, range of motion assessments, ortho, etc. take time - However, it depends upon the institutions appointment routine. I would regard 2 times a year assessment of all the above will pick up stability as well as decline | |

| **Statement** | | **Consensus achieved (yes/no) (%)** |
| --- | --- | --- |
| **Routine physical examination for MPS IVA/VI patients can also identify signs of potential respiratory problems, such as an enlarged tongue or sniff position** | | Yes (90) |
| **Comments** | | |
| *If you disagree with the statement, please explain why and suggest an amendment below:* | - Statement 2 should be incorporated into first statement about PE and does not warrant a key statement - Symptoms are different in children with MPS IV and VI, recommendation for follow cannot be the same - It may show this, but I feel a full respiratory assessment by an expert is also needed - The question is poorly worded. Most potential respiratory problems will not be pick-up on routine exams. PFT's and sleep study are much better in identify potential respiratory problems | |
| *Additional comments or suggestions (Optional):* | - Any staff member who is an expert in MPS disorders will pick an enlarged tongue and observe the sniff position signifying a respiratory decline. That’s why ultra-rare disorders should be managed in centers of excellence | |

| **Statement** | | **Consensus achieved (yes/no) (%)** |
| --- | --- | --- |
| **While X-rays are essential to identify the natural history of disease and response to treatment, efforts should be made to minimize radiation exposure throughout the patient’s lifetime, and images should be requested only when clinically useful** | | Yes (85) |
| **Comments** | | |
| *If you disagree with the statement, please explain why and suggest an amendment below:* | - Subclinical deterioration is common in MPS patients. Regular radiographic surveillance is necessary - It is important to identify problem early, if this requires a couple of more X-rays this is likely to be a lower burden than identifying problem too late - It's not only a clinical criterion, some X-rays have to be done yearly to be able to define indications for operations. And to have the possibility to assess changes over time - This is a dangerous area because we always wish to avoid ionizing radiation, but this recommendation may lead to skipping X-rays when they would be very useful. It may also embolden parents to request no X-rays when in fact they are indicated. So overall, yes, we should minimize these images should be requested when the clinician feels they might be helpful. I don't think it should be included, I would amend it out, we always think before we X-ray patients, it is not necessary to reinforce this - Upper C-spine requires regular radiological review. Thoracolumbar can be assessed clinically - The emphasis should be on a positive statement of indications for X-ray rather than 'avoid unless useful' i.e. annually / biannually and/or with clinical need - What does "clinically useful" mean. While it is difficult to find fault with this statement, it is necessary to get X-rays routinely in these patients, including when they are asymptomatic | |
| *Additional comments or suggestions (Optional):* | - Particularly in early childhood, otherwise annual or q6m radiographs are probably not excessive | |

Radiology

| **Statement** | | **Consensus achieved (yes/no) (%)** |
| --- | --- | --- |
| **An anteroposterior (AP) pelvis radiograph should be performed at diagnosis and as clinically indicated (based on physical examination or reports of pain) for MPS IVA/VI patients to quantify hip dysplasia, or to identify early signs of hip migration** | | Yes (88) |
| **Comments** | | |
| *If you disagree with the statement, please explain why and suggest an amendment below:* | - Clinical signs of hip disease may be subtle or modified by other musculoskeletal conditions - I would do more frequent to detect changes - I think, as with CP, we should be more pro-active in X-raying routinely at certain age points until we understand the natural history of the condition (now that it has been altered by medical treatments) better - Again, "as clinically indicated (based on physical examination or reports of pain)" is a misleading statement. It sounds benign. However, most patients develop a gradual subluxation of the hip that is totally asymptomatic and not apparent clinically. It is only by getting routine, annual, AP pelvis radiographs that one can diagnose and then monitor hip subluxation. By the time it is apparent on physical examination or producing pain, it is too late | |
| *Additional comments or suggestions (Optional):* | - We do the hip X-rays yearly | |

| **Statement** | | **Consensus achieved (yes/no) (%)** |
| --- | --- | --- |
| **In MPS IVA/VI patients with clinical evidence of valgus deformity of the lower limbs, standing AP radiographs of lower extremities should be performed prior to guided growth surgery** | | Yes (100) |
| **Comments** | | |
| *If you disagree with the statement, please explain why and suggest an amendment below:* |  | |
| *Additional comments or suggestions (Optional):* | - We must not rely on clinical evidence alone. This does not allow you to identify the main site of the deformity, and a well-positioned (and this is important) and a well interpreted X-ray is better at providing comparative information both in a patient over time and between patients for assessment of outcomes | |

| **Statement** | | **Consensus achieved (yes/no) (%)** |
| --- | --- | --- |
| **Plain radiography of cervical and thoracolumbar spine is recommended at diagnosis and then every 2-3 years in MPS IVA/VI patients** | | No (74) |
| **Comments** | | |
| *If you disagree with the statement, please explain why and suggest an amendment below:* | - Or when clinically indicated - Plain radiography of the spine should be done only when clinically useful (see questions above!) - What is the evidence to routinely perform every 2 to 3 years? I would suggest "as required based on clinical need" - Would think we need this more frequent especially in young children - other methods are better for the follow up including MRI, electrophysiology - We prefer usage of MRI (see below) - It depends on the age at diagnosis and the current age of patient. To do x rays when clinically needed would be my approach rather doing it as routine at a fixed interval - We rely on MRI in the first instance - I would suggest wording to include "plus as clinically indicated" and also or other appropriate imaging techniques e.g. CT may be used in place of plain radiography in some clinical situations - In patients who underwent spine MRI, plain radiography can be spared | |
| *Additional comments or suggestions (Optional):* | - This is in part dependent on the availability and quality of MRI imaging of the spine at the respective centers. Both MRI and radiography should be applied in a complementary way for early recognition of relevant instability and myelon compression - Or earlier, for example prior to other surgical interventions - please specify including AP and lateral views - In my opinion, annually on a watch and see basis then early signs of compression can be picked up before any further neurological signs of tightening are present | |

| **Statement** | | **Consensus achieved (yes/no) (%)** |
| --- | --- | --- |
| **Magnetic resonance imaging (MRI) of the whole spine (in neutral position) should be performed annually in MPS IVA/VI children to assess for spinal cord injury. The frequency may be reduced for stable adult patients that do not display symptoms** | | Yes (84) |
| **Comments** | | |
| *If you disagree with the statement, please explain why and suggest an amendment below:* | - Also, this investigation should be performed only when clinically useful, as in very young children this procedure may require general anesthesia - See below, neutral imaging is probably insufficient in the cervical regions - It is very difficult in poor countries and MPS IVA have anesthetic risk for these procedures - Do we know that yearly MRI are indicated? Shouldn’t there be a certain threshold for going two years, or ages at which we can go two years? these kids need anesthesia for the MRI and that is not without risk - Initially & then as clinically indicated, particularly when a GA is required - I do not think that it has to be done annually. At least every 2 years and more frequently depending on the previous imaging concerns - Initially and then yearly only if there is a concern (due to clinical or radiological findings) | |
| *Additional comments or suggestions (Optional):* | - If the patients are in need of GA, to perform this then the risks of GA have to be considered - I would rather use the phrase 'cervicomedullary compression' than 'spinal cord injury' - The frequency of MRI of whole spine should be personalized for each patient according to their clinic. Considering MPS IV patients show a higher risk of spinal injuries, their frequency could be annual, but in MPS VI patients the frequency could vary according to the clinic - In my opinion, annually on a watch and see basis then early signs of compression can be picked up before any further neurological signs of tightening are present | |

| **Statement** | | **Consensus achieved (yes/no) (%)** |
| --- | --- | --- |
| **Flexion/extension MRI of cervical spine in MPS IVA/VI patients may be needed to identify changes in spinal canal and spinal cord** | | Yes (86) |
| **Comments** | | |
| *If you disagree with the statement, please explain why and suggest an amendment below:* | - Some and even many of our pediatric patients need general anesthesia for MRI scans. As such performing a forced flexion/extension MRI under general anesthesia sounds very risky to me. I would not allow this in any patient who needs sedation or general anesthesia for the MRI scan - Flex extension should only be done if clinically indicated or in preparation for decompression - We do not think that flexion/extension MRI is indicated - Extension MRI is useless and prolong sedation when the latter is required. Neutral cervical spine MRI should be performed and if no critical stenosis is present, the sole flexion MRI should be performed | |
| *Additional comments or suggestions (Optional):* | - Yes, when the patient is completely awake | |

| **Statement** | | **Consensus achieved (yes/no) (%)** |
| --- | --- | --- |
| **MRI of the brain is recommended at diagnosis in MPS IVA/VI patients to assess for hydrocephalus, with follow up every 2–3 years** | | No (66) |
| **Comments** | | |
| *If you disagree with the statement, please explain why and suggest an amendment below:* | - Clinical and ophthalmic signs of hydrocephalus should be monitored and be indicative for MRI - Would not be dogmatic about this, as clinically indicated - I am unaware of any cases of hydrocephalus in MPS IV - An initial imaging study is needed and repeated as clinically necessary regular monitoring can be done with tape measure - In MPS VI yes, in MPS IVA not - This investigation should be done at diagnosis, but later only when clinically indicated because of the risk of anesthesia - Only when clinical suspicion, not routine - Would do every year during first 1 years, less frequent (2-3 years interval) thereafter - We tend not to see hydrocephalus in MPS IVA even VI patients; as such MRI of brain is typically not performed - I'm not sure of this. I think in the presence of symptoms such as papilledema/headache/evidence of ventricular enlargement on the scout view of the MR spine then yes, but routinely performing MR of brain is likely to prolong scan time excessively for little gain - Agree for MPS VI, but not MPS IVA | |
| *Additional comments or suggestions (Optional):* | - If the patients are in need of GA to performer this then the risks of GA have to be considered - And as needed based on symptoms. Then we should list the symptoms - The frequency of hydrocephalus varies among MPS IV and MPS VI. Thus, in MPS VI patients, the frequency of this test varies depending on the evolution of the disease | |

| **Statement** | | **Consensus achieved (yes/no) (%)** |
| --- | --- | --- |
| **MRI of the brain and spinal cord in MPS IVA/VI patients may require sedation or general anesthesia, depending on patient age and cooperation. General anesthesia carries substantial risk for MPS patients** | | Yes (95) |
| **Comments** | | |
| *If you disagree with the statement, please explain why and suggest an amendment below:* | - General anesthesia is recommended at our institution because sedation provides unpredictable depth which may compromise the airway - I don't think the risk is 'substantial' for an MRI | |
| *Additional comments or suggestions (Optional):* | - Yes, there is risk, but in trained anesthesiology hands MPS patients can be safely sedated | |

| **Statement** | | **Consensus achieved (yes/no) (%)** |
| --- | --- | --- |
| **Computerized tomography (CT) of neutral region of interest may be considered in MPS IVA/VI patients if MRI is not available or if sedation is not possible** | | No (69) |
| **Comments** | | |
| *If you disagree with the statement, please explain why and suggest an amendment below:* | - I don't know what the neutral region of interest is - Not informative enough, encourage to go to center with experience - We never had this case, but if there is no other possibility, yes - Suggest that flexion extension would be extremely important especially in the absence of MRI flexion/extension or c-spine flex/ext X-ray that is likely to be non-interpretable in MPS VI patients. The use of CT statement needs to be re-examined - Exposure to radiation is significant with CT and to be considered if there is a clinical need - What is "neutral region?" - This should be carefully considered. MRI should always be available in a center caring for MPSVI and MPS IVA. If sedation is not possible, how would the child manage a CT? CT is a lot of radiation and there may be a role for it in surgical planning, but I don't think it should be recommended for surveillance without very good reason - We use CT and MRI, CT as a tool pre-surgery to direct surgical technique - I am unclear regarding the word "neutral" suggest "Computerized tomography (CT) of region of interest may be considered in MPS IVA/VI patients if MRI is not available or if sedation is not possible" - I don't understand the "neutral region of interest" concept - Unclear what is the meaning of "neutral region of interest" | |
| *Additional comments or suggestions (Optional):* | - "If MRI is not available, contraindicated..." - CT may be considered before a surgical intervention on the spine, to highlight the morphology of the deformed vertebrae | |

| **Statement** | | **Consensus achieved (yes/no) (%)** |
| --- | --- | --- |
| **The presence of specific radiological signs in MPS IVA/VI patients may indicate the need for surgical intervention to correct skeletal deformities; however, there is insufficient evidence to support preventative surgery based on radiological findings** | | Yes (88) |
| **Comments** | | |
| *If you disagree with the statement, please explain why and suggest an amendment below:* | - I think there is some evidence that significant upper cervical instability needs correction - We operate on asymptomatic genu valgum and on asymptomatic atlanto-axial instability, so these are indeed preventative " however one should document either progressive deformity or near critical values before proceeding on a prophylactic basis" - We feel that there is evidence (from our unit - submitted for publication). That one should operate before clinical signs are present - There is insufficient evidence to support preventive surgery based on radiological findings. I think there is evidence, albeit it not great in the forms of case reports or limited retrospective case series, that preventive surgery is effective   I would eliminate that clause from the statement | |
| *Additional comments or suggestions (Optional):* | - This is an unclear statement. Preventative surgery in what clinical setting? | |

Endurance

| **Statement** | | **Consensus achieved (yes/no) (%)** |
| --- | --- | --- |
| **Choice of assessment depends on MPS IVA/VI patient’s physical and developmental abilities** | | Yes (92) |
| **Comments** | | |
| *If you disagree with the statement, please explain why and suggest an amendment below:* |  | |
| *Additional comments or suggestions (Optional):* | - Yes, we can choose the assessments based on abilities but too much variation in the assessments will not help us when we look at the outcomes as a cohort for the effectiveness of therapy. It will be prudent to agree to the assessments which are doable by the majority of the patients and are likely to be completed and then make minor variations if needed - It would be helpful with a selection of research protocols / MPS customized protocols for different professions so that a summary of various issues can be performed in collaboration between several centers | |

| **Statement** | | **Consensus achieved (yes/no) (%)** |
| --- | --- | --- |
| **Baseline assessment is the most important and ideally two values should be obtained as a minimum. Consistent protocols should be used when performing repeat measurements to minimize variability** | | Yes (95) |
| **Comments** | | |
| *If you disagree with the statement, please explain why and suggest an amendment below:* | - This is great for clinics that are able to do them. Endurance assessments are not done at my institution - Unfortunately, we do not have the appropriate space or time during clinic visits for these assessments. We rely on parent/patient reported outcomes, physical exams, and non-endurance related evaluations to assess treatment efficacy. I do not think the results of a 6MWT test would change our management decisions - This statement is totally out of touch with practical clinical care and makes the critical error of consideration of care models for the MPS patient within a clinical trial environment. This is not practical not necessary to ensure optimal care of the patient | |
| *Additional comments or suggestions (Optional):* | - What does this mean? What is "choice of assessment"? Didn't we just say that there should be a set grid that should be completed with every visit? - I think that "two values should be obtained as a minimum" is too strong. We could suggest having two values of some critical evaluations | |

| **Statement** | | **Consensus achieved (yes/no) (%)** |
| --- | --- | --- |
| **Annual endurance testing using the 6-minute walk test (6MWT) for MPS IVA/VI patients is recommended, as per the American Thoracic Society guidelines** | | Yes (87) |
| **Comments** | | |
| *If you disagree with the statement, please explain why and suggest an amendment below:* | - Not completely disagree, and realize there is no other validated test available, but doubt this is best to use - I find 6MWT for MPS patients, while great for obtaining FDA approval for the drugs, is of questionable benefit aside from justifying usage of drug for some insurance companies. Certainly, it does not assist me in decision making for ERT efficacy - Although 6MWT has been widely used in the clinical trials, in clinical practice patients find it rather too tiring and exhausting afterwards. Quite often patients either can’t do it or don't want to do it. We should consider timed test as the first line test and can be used uniformly across all group of patients and 6MWT as the additional test where patients can manage it - This is great for clinics that are able to do them. Endurance assessments are not done at my institution - Unfortunately, we do not have the appropriate space or time during clinic visits for these assessments. We rely on parent/patient reported outcomes, physical exams, and non-endurance related evaluations to assess treatment efficacy. I do not think the results of a 6MWT test would change our management decisions | |
| *Additional comments or suggestions (Optional):* | - Obviously the 6MWT is best established and thus should be done for now. Yet it is far from ideal as its substantial susceptibility to bias (inter-observer variability, strong impact of motivation etc.) leads to a low internal and external validity even under the very controlled circumstances of a clinical trial and even more in clinical routine. Thus, better alternatives are urgently needed. Hopefully the increasing availability and functions of wearables and health tracking platforms can be utilized for a more reliable/relevant monitoring of endurance - We used 6MWT, but it is not possible in small children with MPS IV and VI and in MPS IV patients in wheel chair. Results in trials are often problematic, they were correlated to age groups (not to exact age, growth and knee and hip mobility) - At least yearly but preferably 6 months - I am not sure this is the best way to assess them but at the moment it is the gold standard. Personally, I think we should be using mobile technology such as fitness trackers which are now cheap and durable - Doubtful if this is the optimal test for endurance when the MPS patient´s performance also depend on joint function and pain - The test provides more information about mobility - Many of these patients are developmentally or physically unable to do reliable testing - This is age dependent | |

| **Statement** | | **Consensus achieved (yes/no) (%)** |
| --- | --- | --- |
| **In MPS IVA/VI patients with limited ambulation who are unable to do the 6MWT, endurance should be assessed via alternative methods such as an adapted timed 25-foot walk test (T25FW)** | | Yes (76) |
| **Comments** | | |
| *If you disagree with the statement, please explain why and suggest an amendment below:* | - Neutral on this statement. Would consider other clinical indicators, such as pain, PFT's, CO2 retention, etc. - I don't disagree - Not completely disagree and realize there is no other validated test available, but doubt this is best to use - See justification against endurance testing above - This is great for clinics who are able to do them. Endurance assessments are not done at my institution. Unfortunately, we do not have the appropriate space or time during clinic visits for these assessments. We rely on parent/patient reported outcomes, physical exams, and non-endurance related evaluations to assess treatment efficacy. I do not think the results of a 6MWT test would change our management decisions - Not sure. My experience is that if ambulation is limited standard measurements are not great. A fitness tracker would be better - It may be an option for patients with limited ambulation, but it had never been tested in MPS to my knowledge - I am not aware that the T25FW test has been validated - Very limited evidence to support this | |
| *Additional comments or suggestions (Optional):* | - As an alternative T25FW may be used, yet it shares the same limitations as 6MWT and should be replaced our supplemented by novel methods - These are less well standardized but can be used - Same as above - Doubtful if this is the optimal test for endurance when the patient´s performance also depend on joint function and pain. The test provides more information about mobility | |

| **Statement** | | **Consensus achieved (yes/no) (%)** |
| --- | --- | --- |
| **Endurance testing in MPS IVA/VI patients is also recommended prior to initiation of ERT and annually thereafter as a measure of treatment efficacy and to provide early evidence of possible neurologic or skeletal issues** | | Yes (87) |
| **Comment** | | |
| *If you disagree with the statement, please explain why and an amendment below:* | - Not completely disagree and realize there is no other validated test available, but doubt this is best to use - See justification against endurance testing above - Very limited evidence to support this - Yes, but one can't get a meaningful 6MWT in a 2 year old - This is great for clinics who are able to do them. Endurance assessments are not done at my institution. Unfortunately, we do not have the appropriate space or time during clinic visits for these assessments. We rely on parent/patient reported outcomes, physical exams, and non-endurance related evaluations to assess treatment efficacy. I do not think the results of a 6MWT test would change our management decisions | |
| *Additional comments or suggestions (Optional):* | - Other factors that would change the mobility during interval period should be taken into account like joint surgeries, acute illness etc. - As able | |

Growth

| **Statement** | | **Consensus achieved (yes/no) (%)** |
| --- | --- | --- |
| **Assessment of growth for MPS IVA/VI patients should be performed at each clinic visit (ideally every 6 months) as part of a regular physical examination and should include: standing height (sitting height if the patient is unable to stand), length (supine position), weight, head circumference (≤3 years), Tanner pubertal stage (until maturity)** | | Yes (95) |
| **Comments** | | |
| *If you disagree with the statement, please explain why and suggest an amendment below:* | - Unclear the benefits of both standing/sitting height and length | |
| *Additional comments or suggestions (Optional):* | - Sitting height may be useful in any case to understand the ratio between trunk and legs - Development of deformities such as scoliosis affect growth and should be taken into account - All these patients have extremely limited growth and therefore measuring it isn’t going serve a useful purpose | |

| **Statement** | | **Consensus achieved (yes/no) (%)** |
| --- | --- | --- |
| **Height and weight of MPS IVA/VI patients should also be measured before initiation of ERT and at every clinic visit thereafter (ideally every 6 months) to evaluate the impact of treatment** | | Yes (95) |
| **Comments** | | |
| *If you disagree with the statement, please explain why and suggest an amendment below:* | - There are multiple reasons for doing this not just the one stated | |
| *Additional comments or suggestions (Optional):* | - Weight is the most important as the dose is weight related unless BMI is to be used, therefore height would have to be recorded. In my experience the height of the patient isn’t affected by the drug. The weight of these patients is the biggest issue, especially the MPS IV patients as their calorific intake is always far higher than is required. These patients have significant mobility issues | |

Urinary keratan sulphate (KS)/glycosaminoglycan (uGAG) levels

| **Statement** | | **Consensus achieved (yes/no) (%)** |
| --- | --- | --- |
| **Where available tandem mass spectrometry may be used to assess levels of urinary KS prior to starting elosulfase alfa and every 6 months thereafter to determine the pharmacodynamic effects of ERT treatment in MPS IVA patients** | | Yes (94) |
| **Comments** | | |
| *If you disagree with the statement, please explain why and suggest an amendment below:* | - Yearly is sufficient for most patients - Benefit of monitoring urine GAG does not correlate with efficacy of treatment | |
| *Additional comments or suggestions (Optional):* | - I agree that it is possible to assess if ERT is decreasing the levels of urinary KS, but I cannot make any other determinations of efficacy beyond that | |

| **Statement** | | **Consensus achieved (yes/no) (%)** |
| --- | --- | --- |
| **Total uGAG levels are often elevated in neonates and infants with MPS IVA, and may overlap with normal values in adults and some teenagers. However, if a specific KS assay is not available, measurement of uGAG levels using standard dye-binding methods may be useful. Preferably, measurements should be performed in the same laboratory and assessed against age-related reference values** | | Yes (85) |
| **Comments** | | |
| *If you disagree with the statement, please explain why and suggest an amendment below:* | - Need to separate the warning about elevated GAG in infants from the fundamental comment about use of uGAG. Same warning about uGAG in the first year also applies for MPS VI - U-GAGs is of importance, but biological markers in blood would be better - Rarely find uGAG levels useful - Although the statement is correct, I do not feel that total uGAG should be used for initial MPS IVA diagnostic purposes particularly in the very young - Suggest "Total uGAG levels are often elevated in neonates and infants with MPS IVA, in adults and some teenagers with MPS IVA total uGAG levels may overlap with normal values. However, if a specific KS assay is not available, measurement of uGAG levels using standard dye-binding methods may be useful. Preferably, measurements should be performed in the same laboratory and assessed against age-related reference values" | |
| *Additional comments or suggestions (Optional):* |  | |

| **Statement** | | **Consensus achieved (yes/no) (%)** |
| --- | --- | --- |
| **Urinary GAG levels should be tested prior to starting galsulfase and every 6 months thereafter to determine the pharmacodynamic effects of ERT in MPS VI patients** | | Yes (97) |
| **Comments** | | |
| *If you disagree with the statement, please explain why and suggest an amendment below:* | - Benefit of monitoring urine GAG does not correlate with efficacy of treatment | |
| *Additional comments or suggestions (Optional):* | - uGAGs are of importance, but biological markers in blood would be better - I agree that it is possible to assess if ERT is decreasing the levels of urinary KS, but I cannot make any other determinations of efficacy beyond that - Best we have but would be nice to have better markers | |

*Urinary glycosaminoglycan (uGAG) level*

| **Statement** | | **Consensus achieved (yes/no) (%)** |
| --- | --- | --- |
| **Measurement of total uGAG levels in MPS VI patients may be performed using standard dye-based quantitative methods, preferably in the same laboratory and assessed against age-related reference values** | | Yes (93) |
| **Comments** | | |
| *If you disagree with the statement, please explain why and suggest an amendment below:* | - May be but not as good as MS/MS - I can't speak to how available the mass spec GAG testing is, but that would seem preferable | |
| *Additional comments or suggestions (Optional):* | - Specific quantitation of dermatan sulfate will be better than total GAGs | |

| **Statement** | | **Consensus achieved (yes/no) (%)** |
| --- | --- | --- |
| **Where available tandem mass spectrometry may be used to assess levels of specific GAGs (such as dermatan sulfate [DS]) in MPS VI patients** | | Yes (97) |
| **Comments** | | |
| *If you disagree with the statement, please explain why and suggest an amendment below:* |  | |
| *Additional comments or suggestions (Optional):* | - u-GAGs are of importance, but biological markers in blood would be better | |

| **Statement** | | **Consensus achieved (yes/no) (%)** |
| --- | --- | --- |
| **Initial cardiac evaluation should be performed at the time of diagnosis in MPS IVA/VI patients and include assessment of vital signs with measurement of oxygen saturation, right arm and leg blood pressure measurements, careful auscultation, full transthoracic two-dimensional and Doppler echocardiogram, and 12-lead electrocardiogram (ECG)** | | Yes (100) |
| **Comments** | | |
| *If you disagree with the statement, please explain why and suggest an amendment below:* |  | |
| *Additional comments or suggestions (Optional):* | - Not sure how practical would it be to perform lower limb BP. Looking at all peripheral pulses may be sufficient - Absolutely, should be part of the basic assessment | |

Cardiac function

| **Statement** | | **Consensus achieved (yes/no) (%)** |
| --- | --- | --- |
| **Longer ECG monitoring (prolonged Holter/Event monitoring) may be considered in older MPS IVA/VI patients especially if they have symptoms of black outs, unexpected falls and dizziness** | | Yes (96) |
| **Comments** | | |
| *If you disagree with the statement, please explain why and suggest an amendment below:* |  | |
| *Additional comments or suggestions (Optional):* | - We have been doing this in adult LSD patients and have found more episodes of arrhythmia than clinically suspected - Especially in the setting of some mitral or aortic valve pathology | |

| **Statement** | | **Consensus achieved (yes/no) (%)** |
| --- | --- | --- |
| **Follow-up in expert centers should be annually initially but may be extended to every 2–3 years if there is no evidence of cardiac abnormality in MPS IVA/VI patients** | | Yes (92) |
| **Comments** | | |
| *If you disagree with the statement, please explain why and suggest an amendment below:* | - We continue to follow patients annually because onset of airway compromise (tracheal redundancy/vascular sling) as well as valvar disease and root dilatation can develop within 1 year - I feel that annual evaluations are optimal | |
| *Additional comments or suggestions (Optional):* | - We do yearly till 1, less frequent thereafter (every 2-3 years) - Is this referring to expert cardiac centers? - This is what we do in some of the older adults who show no abnormality, but they do not get discharged from follow up | |

| **Statement** | | **Consensus achieved (yes/no) (%)** |
| --- | --- | --- |
| **Additional cardiac assessment, including a standard ECG, should be performed prior to any surgical procedures requiring general anesthesia in MPS IVA/VI patients** | | **Yes (92)** |
| **Comments** | | |
| *If you disagree with the statement, please explain why and suggest an amendment below:* | - Depends on how recently this has been performed. Should say within x months - I think the cardiologist should be asked to comment based on their previous assessment. If assessments have been stable for many years, then a repeat assessment just because of surgery may not be necessary | |
| *Additional comments or suggestions (Optional):* | - Absolutely - Never operate without a cardiac CT, cardiac catheter/coronary angiogram, and detailed Echocardiographic assessment of LV outflow tract/mitral valve annulus. If in doubt a stress ECHO can be performed - Decision on additional examination (i.e. echo) case by case - I agree with statement, unless a recent 3 to 4 mo cardiac assessment has been done | |

Neurological examination

| **Statement** | | **Consensus achieved (yes/no) (%)** |
| --- | --- | --- |
| **A detailed neurological examination should be performed in MPS IVA/VI patients at every clinic visit (minimally every 6 months) and, where possible, these should correlate with imaging studies of the spine to detect early spinal stenosis or instability compromising the cervical cord. For patients without clinical or radiographic concern, annual neurological examination may be sufficient** | | Yes (87) |
| **Comments** | | |
| *If you disagree with the statement, please explain why and suggest an amendment below:* | - Evaluation every six months is needed when very young (up to 3-5 years, but not afterwards, once pathology (or lack thereof) is established - A detailed neurological examination minimum of twice a year if the patient is asymptomatic seems excessive to me. "A detailed neurological examination should be performed in MPSIVA/VI patients twice a year at clinic visits..." - I think this needs qualifying - some will take a "detailed neurological examination" to include every modality taught at medical school. This is not necessary and certainly not practical. However, a thorough assessment of tone, power and reflexes is appropriate as well as maybe a screening assessment of sensory modalities and coordination. A good gait assessment will satisfy much of this - Annual not 6 monthly - Depends on what imaging shows | |
| *Additional comments or suggestions (Optional):* | - Considering the progressive behavior of the disease, the neurological assessment should be done every six months with or without radiographic signs | |

| **Statement** | | **Consensus achieved (yes/no) (%)** |
| --- | --- | --- |
| **Flexion/extension cervical spine MRI should be considered for all MPS IVA/VI children with an abnormal neurological examination result** | | No (74) |
| **Comments** | | |
| *If you disagree with the statement, please explain why and suggest an amendment below:* | - Standard MRI is the baseline study. Flexion/extension MRI should be done for selected cases with dissociation of clinical and standard radiographic data - Considered yes, but if sedation or general anesthesia is required, the risk outweighs any possible benefits in my opinion - Static MRI may answer the question of etiology of the examination anomaly. F/E MRI scans may not be easily available in some locations. The degree of motion to be achieved between F and E has not been specified and therefore is not reproducible in my experience (varies with the radiologist and his or her risk tolerance). So, a static MRI should be used first, and a clinical F/E test added to the physical examination - Static MRI may answer the question of etiology of the examination anomaly - F/E MRI scans may not be easily available in some locations. The degree of motion to be achieved between F and E has not be specified and therefore is not reproducible in my experience (varies with the radiologist and his or her risk tolerance) - It could be much risky, mainly if the child is sedated. If this child has abnormal neurological signs a lesion should be evident. Somatosensory evoked potentials may also help - We do not do flexion/extension under GA - Difficult to agree with an "all" statement here - Imaging should be done first in neutral to see if it is safe to flex and extend the patient often under GA. Then, based on the findings, do flexion and extension. But also, it depends on what is abnormal, abnormal needs to be defined. New weakness in LE with normal UE could be related to kyphosis. It would be a waste of time to do cervical spine - A spine MRI should be performed with an abnormal neuro exam, but doubt if flexion/ext MRI should be done before knowing the results of the standard cervical spine MRI | |
| *Additional comments or suggestions (Optional):* | - Extension cervical spine MRI should be avoided as useless; flexion cervical spine MRI should be performed only after careful evaluation of the exam in neutral position and after having excluded severe cervical stenosis or myelopathy | |

| **Statement** | | **Consensus achieved (yes/no) (%)** |
| --- | --- | --- |
| **Symptoms of CTS are often atypical in patients with MPS VI, therefore recommend clinical examination, assessment of range of finger movement and strength, electrophysiology nerve conduction assessment and detailed medical history to be performed at diagnosis and annually thereafter** | | Yes (89) |
| **Comments** | | |
| *If you disagree with the statement, please explain why and suggest an amendment below:* | - A careful clinical/neurological examination should be performed at diagnosis and annually, but neurophysiological investigations only if clinically indicated - The main features would be sweating/wasting and NCS. A history of chewing fingers, increased clumsiness might be helpful but mostly the loss of function is not really noticed until after decompression when the parents report a subtle improvement - It is unclear to me how often NCV studies should be performed | |
| *Additional comments or suggestions (Optional):* | - Interval of assessment can be extended when patients old enough to relay symptoms. - Results may show a worse deficit than clinically apparent - It is to be considered that the electrophysiology nerve conduction assessment can have weaknesses, since the anatomy of patients with MPS is different - Standardization of parameters for nerve conduction study is necessary and must be performed within standard temperature with notation of intensity of stimulation, palmar sensory stimulation and detail of wave form | |

| **Statement** | | **Consensus achieved (yes/no) (%)** |
| --- | --- | --- |
| **Reach-out tests or the Pediatric Orthopedic Society of North America (POSNA) Pediatric Musculoskeletal Functional Health Questionnaire may also be used to assess hand and upper limb function in MPS VI patients** | | No (72) |
| **Comments** | | |
| *If you disagree with the statement, please explain why and suggest an amendment below:* | - Not validated in all countries - I've never used these - Not sure what reach out tests are? Need to be specific in key guidelines - Since the symptomatology can be atypical, I'm not sure that the POSNA is appropriate as it is quite general - And/or the PODCI assessment tool | |
| *Additional comments or suggestions (Optional):* |  | |

*Upper limb function*

Respiratory function and sleep disorder

| **Statement** | | **Consensus achieved (yes/no) (%)** |
| --- | --- | --- |
| **Evaluation of respiratory function by spirometry, including forced vital capacity (FVC) and maximum voluntary ventilation (MVV), should be performed to assess changes in lung volume and obstruction on MPS IVA/VI children over 5 years of age** | | Yes (97) |
| **Comments** | | |
| *If you disagree with the statement, please explain why and suggest an amendment below:* | - Lung volumes, maximum inspiratory and expiratory pressures should also be measured | |
| *Additional comments or suggestions (Optional):* | - With the proviso that technique is variable after 5 years even. I would state that technique can be variable in patients aged 5 - 8 years, if cognitively impaired or in patients with behavioral issues such as ADHD. Hence data may be unreliable. Please see guidelines re respiratory function tests - Lying and sitting FVC is ideal rather than only sitting lung functions - 5 years may not be the right cut off for this test - Because normative values are missing, the results need to be weighted via the personal stamina or any decreasing tolerance to physical exercise. - Not all children over 5 may be able to do these tests - Suggest "once children are old enough to be able to reliably perform the tests" - Unclear if MMV is part of standard of care | |

| **Statement** | | **Consensus achieved (yes/no) (%)** |
| --- | --- | --- |
| **Respiratory function should be assessed annually until MPS IVA/VI children stop growing, and every 2–3 years thereafter provided that respiratory symptoms remain unchanged. Additional testing should be performed if respiratory symptoms change or if intercurrent illnesses occur** | | Yes (91) |
| **Comments** | | |
| *If you disagree with the statement, please explain why and suggest an amendment below:* | - PFTs should be tested annually, it is a marker of declining pulmonary reserve and worsening bony disease/endurance (plus survival, it seems) when it is declining - We do PFT as baseline and whenever patient develops new symptoms - Annually would be better once we could detect changes earlier and would have the chance to provide any intervention - As an adult respiratory intensivist manning a home ventilation service I would suggest annual review of respiratory function | |
| *Additional comments or suggestions (Optional):* | - I would advise annual respiratory function testing | |

| **Statement** | | **Consensus achieved (yes/no) (%)** |
| --- | --- | --- |
| **Normative values are not available, therefore change in absolute volume from MPS IVA/VI patients own baseline will be the best indicator of deterioration or improvement** | | Yes (97) |
| **Comments** | | |
| *If you disagree with the statement, please explain why and suggest an amendment below:* | - This statement applies after the growth period only. It is correct for "deterioration" | |
| *Additional comments or suggestions (Optional):* | - Because normative values are missing, the results need to be weighted via the personal stamina or any decreasing tolerance to physical exercise - Little option but to use patients baseline | |

| **Statement** | | **Consensus achieved (yes/no) (%)** |
| --- | --- | --- |
| **Measuring respiratory rate and arterial oxygen saturation before and after annual endurance testing is recommended in MPS IVA/VI patients** | | Yes (86) |
| **Comments** | | |
| *If you disagree with the statement, please explain why and suggest an amendment below:* | - 6 minutes walking test - Not sure this is helpful - Any evidence of value in clinical studies? - Should be undertaken with annual monitoring of lung function and overnight oximetry | |
| *Additional comments or suggestions (Optional):* | - Arterial oxygen saturation will be tested by oximeter and not by arterial puncture - Would advise ear lobe blood gas before and after annual endurance testing in addition | |

| **Statement** | | **Consensus achieved (yes/no) (%)** |
| --- | --- | --- |
| **Evaluation of gas exchange and respiratory function is also recommended before any planned air travel, to ensure safety during the flight in MPS IVA/VI patients** | | Yes (86) |
| **Comments** | | |
| *If you disagree with the statement, please explain why and suggest an amendment below:* |  | |
| *Additional comments or suggestions (Optional):* | - Probably useful. Never done in my patients - I am not sure if we need this in all patients including the ones with normal or near normal morphology | |

| **Statement** | | **Consensus achieved (yes/no) (%)** |
| --- | --- | --- |
| **MPS IVA/VI patients should be asked to report presence of snoring and morning headaches to identify symptoms of sleep apnea at every clinic visit** | | Yes (100) |
| **Comments** | | |
| *If you disagree with the statement, please explain why and suggest an amendment below:* | - Only if there is respiratory concern. If yearly testing and basic function is normal extra testing for airplane is not needed - Not sure this is helpful and very complicated to organize - Don’t think assessment prior to air travel is required in every circumstance - For planned flights would advise a flight assessment (hypoxic challenge test) even in presence of normal gas exchange at baseline. With severe chest wall restriction | |
| *Additional comments or suggestions (Optional):* | - Snoring and apnea and day time fatigue, tiredness and difficulty to wake up and difficulty to gain weight should be added to rule out or diagnose OSAS - Consider annual oximetry in conjunction with symptom reporting to enable decision around management of OSA/ nocturnal hypoventilation | |

| **Statement** | | **Consensus achieved (yes/no) (%)** |
| --- | --- | --- |
| **Overnight sleep study (polysomnography) is recommended at diagnosis (if possible, and no later than 2 years of age), and every 3 years thereafter or when signs and symptoms of obstructive sleep apnea (OSA) are noted in MPS IVA/VI patients** | | Yes (94) |
| **Comments** | | |
| *If you disagree with the statement, please explain why and suggest an amendment below:* | - Once a year is better especially in the pediatric group. CO2 measurement is mandatory in the overnight psg study for the patients once is the gold standard method to evaluate hypoventilation - Would recommend annual sleep study as above in adult patients | |
| *Additional comments or suggestions (Optional):* | - More frequent in young children - If resources for PSG is limited in the country, geographic region and there are no history or signs and symptoms of OSAS present we may not need to do this in every patient - Likely to present late with signs and symptoms and could regard overnight oximetry annually with annual respiratory assessment | |

Ear-nose-throat (ENT)

| **Statement** | | **Consensus achieved (yes/no) (%)** |
| --- | --- | --- |
| **ENT examination, including tympanometry, should be conducted every 3–6 months during childhood and every 6–12 months thereafter in MPS IVA/VI patients** | | Yes (91) |
| **Comments** | | |
| *If you disagree with the statement, please explain why and suggest an amendment below:* | - 3 monthly seems a bit frequent, most are stable with grommets - ENT examination may be conducted annually if not clinically indicated | |
| *Additional comments or suggestions (Optional):* | - May be a little too often for some patients - Every 6 months seems ok to me in the beginning | |

| **Statement** | | **Consensus achieved (yes/no) (%)** |
| --- | --- | --- |
| **Each ENT examination in MPS IVA/VI patients should include a recorded flexible nasopharyngolaryngoscopy to visualize the upper respiratory tract. If airway obstruction involving a site other than the upper respiratory tract is suspected, rigid endoscopic evaluation under general anesthesia is indicated to assess the whole airway** | | No (69) |
| **Comments** | | |
| *If you disagree with the statement, please explain why and suggest an amendment below:* | - Rigid bronchoscopy requires extreme neck extension. Flexible bronchoscopy can be performed with the neck in neutral position. I suggest the committee consider changing this statement to flexible bronchoscopy - Not every evaluation - Is quite invasive and not sure how it can help. Only when symptoms/complaints - Would leave this very specific rec to individual ENTs judgement. Definitely would not recommend with every visit - Frequent nasopharyngeal endoscopy exam is recommended. But rigid endoscopy exam is performed under general anesthesia. There are risks of general anesthesia such as thoracic hypoplasia and ventilation difficulty for MPS patients. I don't think it is good idea for examination under general anesthesia - The tendency is at the moment, that awake fiber optic evaluation of the upper-airway is left behind, because: 1) this examination gives only limited quality of visibility of the area 2) there is no agreement in the judgment of the findings among ENT-surgeons 3) the awake situation differs strongly from the situation in sleep (muscle relaxation in sleep and change of the tongue position is supine position compared to upright - Both upper airway and lower airways are abnormal in IVA patients. However, rigid bronchoscopy is also quite difficult due to lack of cervical spine mobility and short neck; which means the ENT surgeon cannot extend the neck in order to place the rigid bronchoscopy. With some experience it may be possible in patients who are less affected | |
| *Additional comments or suggestions (Optional):* | - Burden and benefit of this examination has to be judged individually in every single patient - Endoscopic evaluation is always the answer to evaluating respiratory tract obstruction, but flexible endoscopy is preferred over rigid because you get to visualize physiology in action with much less sedation | |

| **Statement** | | **Consensus achieved (yes/no) (%)** |
| --- | --- | --- |
| **Age-adjusted audiometric assessment as a baseline objective hearing evaluation should be conducted at first clinic visit and repeated annually to assess conductive and sensory-neural hearing loss in MPS IVA/VI patients** | | Yes (100) |
| **Comments** | | |
| *If you disagree with the statement, please explain why and suggest an amendment below:* |  | |
| *Additional comments or suggestions (Optional):* | - This could probably be reduced at some point in patients with no hearing loss | |

| **Statement** | | **Consensus achieved (yes/no) (%)** |
| --- | --- | --- |
| **If speech problems are determined during the ENT examination, an assessment by a speech pathologist should be conducted in MPS IVA/VI patients** | | Yes (100) |
| **Comments** | | |
| *If you disagree with the statement, please explain why and suggest an amendment below:* |  | |
| *Additional comments or suggestions (Optional):* |  | |

| **Statement** | | **Consensus achieved (yes/no) (%)** |
| --- | --- | --- |
| **Balance tests should be conducted if the MPS IVA/VI patient has a history of balance problems** | | Yes (95) |
| **Comments** | | |
| *If you disagree with the statement, please explain why and suggest an amendment below:* | - Would not consider as key recommendation. Not sure I would put in the ENT section | |
| *Additional comments or suggestions (Optional):* | - However, this must be very rare as I have not witnessed this in our cohort of patients | |

| **Statement** | | **Consensus achieved (yes/no) (%)** |
| --- | --- | --- |
| **Age-appropriate evaluations by an ophthalmologist to assess ophthalmic function is recommended for MPS IVA/VI patients every 6 months if possible, or at least annually** | | Yes (90) |
| **Comments** | | |
| *If you disagree with the statement, please explain why and suggest an amendment below:* | - I think annually is adequate - I think maybe less frequently in MPS IVA | |
| *Additional comments or suggestions (Optional):* |  | |

Ophthalmological function

| **Statement** | | **Consensus achieved (yes/no) (%)** |
| --- | --- | --- |
| **Ophthalmic assessment for MPS IVA/VI patients may include visual acuity, refraction, slit-lamp examination of cornea, funduscopic evaluation including optic nerve, and measurement of intraocular pressure** | | Yes (100) |
| **Comments** | | |
| *If you disagree with the statement, please explain why and suggest an amendment below:* |  | |
| *Additional comments or suggestions (Optional):* | - This should also include assessment of ocular motility and alignment, and may include contrast sensitivity | |

| **Statement** | | **Consensus achieved (yes/no) (%)** |
| --- | --- | --- |
| **Scotopic and photopic electroretinogram may be performed in MPS IVA patients with clinical suspicion of retinopathy or when considering corneal transplantation** | | Yes (100) |
| **Comments** | | |
| *If you disagree with the statement, please explain why and suggest an amendment below:* |  | |
| *Additional comments or suggestions (Optional):* | - Should also include assessment of visual fields if possible prior to corneal transplantation or if retinopathy is suspected | |

| **Statement** | | **Consensus achieved (yes/no) (%)** |
| --- | --- | --- |
| **Intraocular pressure monitoring and pachymetry may be considered prior to corneal transplant in MPS IVA/VI patients** | | Yes (100) |
| **Comments** | | |
| *If you disagree with the statement, please explain why and suggest an amendment below:* |  | |
| *Additional comments or suggestions (Optional):* | - If ultrasound pachymetry is not possible an alternative would be to use anterior segment OCT to measure corneal thickness - I agree that IOP these are part of routine care, but am not sure why we suggest it in particular prior to corneal transplantation. We know that the IOP will likely be a falsely high reading due to the effect of the increase in thickness in those patients considered for transplant. So, I’m not sure what this statement really adds | |

Evaluation of oral health by dentist

| **Statement** | | **Consensus achieved (yes/no) (%)** |
| --- | --- | --- |
| **Close monitoring of dental development (at least annually) is recommended in MPS IVA/VI patients to prevent caries and attrition of the teeth, and monitoring of occlusion and chewing functions** | | Yes (100) |
| **Comments** | | |
| *If you disagree with the statement, please explain why and suggest an amendment below:* |  | |
| *Additional comments or suggestions (Optional):* | - Especially in MPS VI who have quite a lot of teeth problems | |

| **Statement** | | **Consensus achieved (yes/no) (%)** |
| --- | --- | --- |
| **The need for subacute bacterial endocarditis (SBE) prophylaxis prior to dental procedures in MPS IVA/VI patients should be assessed by a cardiologist** | | Yes (100) |
| **Comments** | | |
| *If you disagree with the statement, please explain why and suggest an amendment below:* |  | |
| *Additional comments or suggestions (Optional):* |  | |

Disease burden

| **Statement** | | **Consensus achieved (yes/no) (%)** |
| --- | --- | --- |
| **Annual assessment of patient-reported outcomes is recommended for: pain severity, quality of life (QoL) as assessed by reproducible and age-appropriate questionnaires (eg EQ-5D-5L), fatigue, and activities of daily living (ADL) as assessed by functional tests (6MWT/T25FW), age-appropriate ADL questionnaires (eg MPS Health Assessment Questionnaire [MPS HAQ]), and assessment of wheelchair/walking aid use** | | Yes (97) |
| **Comments** | | |
| *If you disagree with the statement, please explain why and suggest an amendment below:* | - I'm not sure how these assist in treatment | |
| *Additional comments or suggestions (Optional):* | - One has to be careful in evaluation of these as there will be a placebo effect after start of therapy. Research has proven that more expensive the drug more is the placebo effect. Any other supportive therapy offered should also be factored like pain killers, surgeries, active physio input etc. - An overview of already used questionnaire for patient with MPS should be done in the different centers, for comparison - Again, doing a 6MWT or other testing is error prone in children. Is there an age recommendation here? | |

| **Statement** | | **Consensus achieved (yes/no) (%)** |
| --- | --- | --- |
| **These assessments may have to be adapted both for language, culture and individual physical limitations as they have not been validated in these specific disorders** | | Yes (97) |
| **Comments** | | |
| *If you disagree with the statement, please explain why and suggest an amendment below:* | - For example, the EQ-5D-5L are produced using a standardized translation protocol whether or not they have been validated in these specific disorders | |
| *Additional comments or suggestions (Optional):* | - They should also be adapted to the problem areas of interest in patients with MPS | |

Physical therapy

| **Statement** | | **Consensus achieved (yes/no) (%)** |
| --- | --- | --- |
| **Regular assessments should be conducted for MPS IVA/VI patients by a physical therapist (lower limb), occupational therapist (upper limb) and rehabilitation medicine specialist to assess upper and lower function and provide support as needed** | | Yes (93) |
| **Comments** | | |
| *If you disagree with the statement, please explain why and suggest an amendment below:* | - Fully agree with physio input regularly but all the centers are unlikely to have regular input from OT and rehab team.   Our practice is to get patient reviewed by physical therapist on every visit and further referrals are made depending on the needs   - This is not feasible in our center but if available, I would agree - Would be nice but often not available in clinical practice so assessments often done by clinicians | |
| *Additional comments or suggestions (Optional):* | - I agree but it is difficult to limit the assignment to upper or lower limb, because in various countries different occupations have variant functions/roles - It may be a good way of doing the assessment together, for example physiotherapist and occupational therapist. - Regular assessments should be conducted for MPS IVA/VI patients by a physical therapist (lower limb), occupational therapist (upper limb) and/or rehabilitation medicine specialist to assess upper and lower function and provide support as needed - I would add "whenever possible," as these specialists are not available in every MPS care service | |

| **Statement** | | **Consensus achieved (yes/no) (%)** |
| --- | --- | --- |
| **Physical therapists could also assist in suggesting walking aids and other adaptations that may improve QoL for MPS IVA/VI patients** | | Yes (98) |
| **Comments** | | |
| *If you disagree with the statement, please explain why and suggest an amendment below:* | - This can only be recommended by MDs | |
| *Additional comments or suggestions (Optional):* | - This input in our clinic is of immense help to patients - If you can get an assessment they can be helpful - With guidance from a physiatrist knowledgeable in this area | |

2. Disease-Modifying Interventions

*Enzyme replacement therapy (elosulfase alfa) in MPS IVA*

| **Statement** | | **Consensus achieved (yes/no) (%)** |
| --- | --- | --- |
| **Initiation of life-long ERT with elosulfase alfa at a dose of 2 mg/kg/week through intravenous infusion is recommended in all MPS IVA patients as soon as possible after a confirmed diagnosis** | | Yes (79) |
| **Comments** | | |
| *If you disagree with the statement, please explain why and suggest an amendment below:* | - I am not sure that I agree with "life-long" - There may be very mildly affected patients who hardly benefit from ERT. Reference: Beck M, Glossl J, Grubisic A, Spranger J: Heterogeneity of morquio disease. Clin Genet (1986) 29(4):325-331 - The efficacy of elosulfase and cost benefit is not clear. It seems, that ERT is helpful only in small group of patients, probable very young? - The status of the patient, disease burden and ultimate prognosis at the time of diagnosis needs to be taken into account in the decision to initiate ERT. The patient and family need to share in the decision process. - It depends on severity of the disease and the irreversible lesions - Initiation of lifelong ERT should only be done after a thorough discussion with the patient (and family/guardian) and consideration of the risks and benefits. Some patients with less rapidly progressive disease may choose to delay or not use enzyme replacement therapy if their symptoms can be managed in other ways - e.g. symptomatic care only. Further data on effect of HSCT in MPS VIA is needed to better determine risks and benefits of ERT vs HSCT, alone or in combination - This disorder is predominantly a disorder of the skeletal system and this drug does not get into the bones to make any significant difference. I have managed patients both pre-licensing and post ERT and there is no difference in the patient cohorts. In my opinion this drug does not prevent the significant bone problems and does not prevent decline. The issues that we were managing 15 years ago are still being managed now. The issues have not changed. The drug has not altered the course of the disease. May have slowed it marginally however not enough to prescribe a high cost therapy for such a small benefit, if any | |
| *Additional comments or suggestions (Optional):* | - Here are situations when QOL may mean that ERT is an imposition and is futile in life-limiting situations. Initiation of ERT with elosulfase alfa at a dose of 1 mg/kg/week with intravenous infusion is recommended in all MPS VI patients as soon as possible after a confirmed diagnosis in patients without co-existing life-threatening morbidities - I think it remains to be seen if the treatment should be life-long, but starting ASAP with no clear end-point. I agree strongly - All patients require full assessments. intervention with ERT may not be appropriate in all - In absence of other therapies, I certainly do recommend initiation of ERT, but not with a great deal of conviction with regards to effecting change in the primary area of disease symptoms (bony structures) - Disease status at the time of diagnoses will determine whether patient is likely to benefit by start of ERT or not - I think it is important that the patients are carefully monitored to assess how much improvement the drug is causing or how much deterioration it is preventing - I usually do not say "all", as always there is an exception, as a patient with very advanced disease, restricted to bed, who will have no palpable benefits from ERT - This will depend on the comorbidities and stage of disease progression | |

| **Statement** | | **Consensus achieved (yes/no) (%)** |
| --- | --- | --- |
| **Initiation of life-long ERT with galsulfase at a dose of 1 mg/kg/week through intravenous infusion is recommended in all MPS VI patients as soon as possible after a confirmed diagnosis** | | No (74) |
| **Comments** | | |
| *If you disagree with the statement, please explain why and suggest an amendment below:* | - Some patients have had successful BMT as an alternative. There are situations when QOL may mean that ERT is an imposition and is futile in life-limiting situations. The statement could be modified to say:   Initiation of ERT with galsulfase at a dose of 1 mg/kg/week with intravenous infusion is recommended in all MPS VI patients as soon as possible after a confirmed diagnosis in patients without co-existing life-threatening morbidities   - I am not sure that I agree with "life-long" - With BMT being much safer over last decade (survival over 95-98%), this is a treatment to consider in MPS6 patients as well. (Also in patients with neutralizing antibodies.) Comparison between transplanted and ERT patients is warranted - The efficacy of galsulfase and cost benefit is not clear - The status of the patient, disease burden and ultimate prognosis at the time of diagnosis needs to be taken into account in the decision to initiate ERT. The patient and family need to share in the decision process - I have experience with successful HSCT in MPS IV so not all patients need ERT | |
| *Additional comments or suggestions (Optional):* | - I usually do not say "all", as always there is an exception, as a patient with very advanced disease, restricted to bed, who will have no palpable benefits from ERT | |

Enzyme replacement therapy (galsulfase) in MPS VI

Haematopoietic stem cell transplantation in MPS IVA/VI

| **Statement** | | **Consensus achieved (yes/no) (%)** |
| --- | --- | --- |
| **HSCT should only be considered at diagnosis in exceptional circumstances for young, clinically stable MPS IVA patients who have a matched related (non-carrier) donor, or well-matched unrelated donor or cord blood graft** | | No (62) |
| **Comments** | |  |
| *If you disagree with the statement, please explain why and suggest an amendment below:* | - Young clinically stable patients with MPS IV do as well with ERT, without the inherent risks of HSCT - I suppose it is never wrong to consider anything, but I do not think it is a good treatment option currently - to avoid every week infusions - I don't think it should be considered at all - The evidence in favor of SCT for MPS IVA is insufficient to justify risks of immunocompromise, graft failure, GVHD, and death for this condition - I do not disagree, but the evidence is lacking, and I would not strongly support - I think HSCT may be an option in certain circumstances, should be performed under a research program, in Institutions expert of MPS, after IRB approval of the protocol which should include the long-term follow up. This sentence seems not taking into consideration that it could be done only in the context of an approved research protocol. The approved research protocol is needed because there is not sufficient data proving the superiority of HSCT over ERT - There is no data to support this claim there are merely personal opinions. HSCT has considerable risks and should be considered exploratory and require local ethics approval - I have never known a BMT be carried out in an MPS IV patient, however with increased survival rates in other MPS cohorts a BMT may be a preferable option. However, BMT doesn’t correct bone deformities. - I currently do not recommend HSCT for MPS IVA - I am not aware of evidence related to successful outcome from HSCT in MPSIVA | |
| *Additional comments or suggestions (Optional):* | - Not much data on this - This is to say, that given the missing evidence for effectiveness of HSCT and the limitations of ERT, a trial with HSCT may be justified but only under the above described ideal circumstances - I would suggest "HSCT may be considered in exceptional circumstance" - I feel this wording better expresses the current data which is limited but suggestive of a benefit - Is there a correlative statement that suggests a meeting should occur between a transplant specialist and the family to explore this? Otherwise who is going to determine the suitability of the donor, and initiate these discussions? - There is still little evidence about benefits of HSCT in MPS IV A, but in exceptional circumstances, especially in countries where ERT is not available, this may be an option | |

| **Statement** | | **Consensus achieved (yes/no) (%)** |
| --- | --- | --- |
| **HSCT may be an option at diagnosis for young, clinically stable MPS VI patients who have a matched related (non-carrier) donor, or well-matched unrelated donor or cord blood graft** | | No (69) |
| **Comments** | |  |
| *If you disagree with the statement, please explain why and suggest an amendment below:* | - It's an option, but I do not think it is a good one currently - to avoid every week infusions - As above - Supporting text is identical for MPS IVA and VI--would strongly suggest that the key opinion be the same. - While benefit of SCT for MPS VI is more grounded in evidence, again the degree of risk for complications makes me reluctant to make a 1st line recommendation of SCT for MPS VI - The risks of serious complications and death with HSCT are still too high for this to be a routine option offered to families - I do not disagree, but the evidence is lacking, and I would not strongly support - There is NO data to support this claim there are merely personal opinions. HSCT has considerable risks and should be considered exploratory and require local ethics approval - It is not yet clear whether HSCT offers clinical benefits compared with ERT in MPS VI. Therefore, the decision to undergo transplant should be made with the patient and their family based on their individual situation - I can’t recall a BMT being carried out in an MPS VI patient however, again it’s a disorder of the skeleton for which a BMT will not treat | |
| *Additional comments or suggestions (Optional):* | - This statement is reasonable, but not my personal preference. A similar statement would be appropriate for MPS IV - This is to say, that given the missing evidence for effectiveness of HSCT and the limitations of ERT, a trial with HSCT may be justified but only under the above described ideal circumstances. - I have no experience with HSCT in MPS VI. I would prefer ERT as soon as possible after diagnosis/birth instead of HSCT. But if no other option, I would probably try HSCT (after serious discussion with the parents about the risk of HSCT) - May be an option but in the context of an approved research protocol - I would suggest "HSCT may be an option clinically stable MPS VI....” We have used HSCT in older MPS patients both with and without prior ERT. Our eldest patient with MPS VI is now 15 years post-transplant, and remains stable. He had severe sleep apnea and other complications prior to transplant and has shown similar improvements to patients managed with ERT. Another patient elected to have HSCT after a number of years of ERT having considered the available data for ongoing ERT and HSCT risk/benefits. He had ERT prior to and through the HSCT procedure in a similar way to many MPS 1 patients. Further data and studies need to be considered - Is there a correlative statement that suggests a meeting should occur between a transplant specialist and the family to explore this? Otherwise who is going to determine the suitability of the donor, and initiate these discussions? - Personal experience | |

| **Statement** | | **Consensus achieved (yes/no) (%)** |
| --- | --- | --- |
| **For MPS IVA/VI patients, HSCT should be performed in an institution with a MDT experienced in the care of individuals with MPS and established Institutional Review Board (IRB)-approved protocols** | | Yes (84) |
| **Comments** | |  |
| *If you disagree with the statement, please explain why and suggest an amendment below:* | - Only true if you assume that HSCT is appropriate in the first place - Don't think it should be performed at all - Provided the protocol is considered within the "research" environment - Again, suitability of donors and patient condition are important here as well - I don't think there is a need of IRB approval to perform HSCT in a MPS patient - I am not sure if IRB approval is needed for HSCT in MPS VI | |
| *Additional comments or suggestions (Optional):* | - I do not think that an ethics approval is required for HSCT, this is a clinical decision, but the decision makers need to have clinical experience - HSCT in MPS patients can have different issues than for other conditions e.g. malignancy so an expert MPS center is essential - Not all countries have Institutional Review Board and this needs to be allowed for in the statement e.g. IRB or similar governing body - HSCT should only be carried out in hospitals experienced in the disorders | |

| **Statement** | | **Consensus achieved (yes/no) (%)** |
| --- | --- | --- |
| **HSCT may also be an option for MPS IVA/MPS VI patients who do not tolerate, or cannot access, ERT (for example patients who experience severe adverse events leading to ERT discontinuation) and who meet the above criteria** | | Yes (83) |
| **Comments** | |  |
| *If you disagree with the statement, please explain why and suggest an amendment below:* | - Agree but not terribly enthusiastic about this - Again, risks of SCT outweigh theoretical benefits of treatment, especially for MPS IVA - It is not yet clear whether HSCT offers clinical benefits compared with ERT in MPS VI. Therefore, the decision to undergo transplant should be made with the patient and their family based on their individual situation - I currently would not recommend HSCT for MPS IVA | |
| *Additional comments or suggestions (Optional):* | - Also, patients with a mismatch cord blood available could be candidates for transplants in particular when no other option is available. Outcomes with mismatch cord blood not much worse than fully matched CBT (bit more morbidity) - Always in the context of a research protocol - The numbers of patients for whom ERT is not an option for the above reasons would not persuade me to say that a BMT would be an option with significant benefits, it still remains a skeletal disorder for which a BMT will not correct | |

Anesthetics and surgical interventions

Anesthetics in MPS IVA/VI

| **Statement** | | **Consensus achieved (yes/no) (%)** |
| --- | --- | --- |
| **Pre-, intra- and post-operative care (until extubation is complete) for all procedures requiring general anesthesia, or conscious or deep sedation, should be supervised by an anesthetist with experience in MPS and/or complex airway management. In addition, the anesthetist should have access to Intensive Care support and be surrounded by an experienced team capable of performing emergency tracheotomy if required** | | Yes (98) |
| **Comments** | | |
| *If you disagree with the statement, please explain why and suggest an amendment below:* | - Don't think experience with complex airway substitutes for MPS experience. There is much more to managing MPS that just consideration of the airway | |
| *Additional comments or suggestions (Optional):* | - Patients with MPS IV and especially MPS VI have abnormal airways and a combination of obstructive and restrictive airway disease. They are difficult to intubate and have very little reserve. It is imperative that the anesthetist is experienced, and has appropriate back up in terms of ENT support and PICU/PHDU post-op | |

| **Statement** | | **Consensus achieved (yes/no) (%)** |
| --- | --- | --- |
| **A full assessment of the risks and benefits should take place with the patient and family prior to any procedure. All pre-operative information should be made available to allow decision making** | | Yes (100) |
| **Comments** | | |
| *If you disagree with the statement, please explain why and suggest an amendment below:* |  | |
| *Additional comments or suggestions (Optional):* | - They need to know all risk before the procedure. For example, the need of tracheotomy | |

| **Statement** | | **Consensus achieved (yes/no) (%)** |
| --- | --- | --- |
| **ENT, respiratory, cardiac, and radiological assessment should be performed prior to any procedure requiring anesthesia** | | Yes (93) |
| **Comments** | | |
| *If you disagree with the statement, please explain why and suggest an amendment below:* | - One has to be pragmatic about this - involving all of these teams before every surgery is not possible. If surgery has not been performed for over a year I would do this in a stable patient. What typically happens is that we do this and then the patient has a series of surgeries, so it is not necessary to do it before every procedure - how you phrase that is awkward maybe consider all of the above before surgery rather than do - Depends on how recently these assessments have been performed. Should say these things should be done within x months of surgery. Do not feel radiologic assessments are necessarily needed immediately before surgery. If so which ones? | |
| *Additional comments or suggestions (Optional):* | - Within an appropriate timescale for each patient according to the severity of the comorbidity. - Sleep evaluation should also be performed before anesthesia - And Neurosurgical - It is unclear how helpful radiological assessment will be for most patients | |

| **Statement** | | **Consensus achieved (yes/no) (%)** |
| --- | --- | --- |
| **It is critical to maintain a neutral neck position during all surgeries, and during intubation and extubation to avoid paralysis. Strongly recommend the use of techniques that allow maintenance of the neutral neck position, including use of laryngeal mask airway (LMA) for shorter procedures, or intubation with a video laryngoscope or fiberoptic intubation** | | Yes (87) |
| **Comments** | | |
| *If you disagree with the statement, please explain why and suggest an amendment below:* | - While this is true for many of these patients, this is too rigid. Should state that these precautions are necessary when cervical instability or stenosis are present or suspected - LMA should only be used for short procedure if it has been ascertained that the patient can be intubated promptly if the LMA should fail - In patients with normal anatomy and radiology we need neck extension in some surgeries like T+A - The natural position is not always neutral | |
| *Additional comments or suggestions (Optional):* | - Effective preoperative assessment will identify those patients at greater risk of cervical cord compression. Not all patients are at high risk - Statement is grossly accurate but written wrong from a clinical point of view - Both these groups of patients have spinal cord compression almost always requiring cervical fusion by early adolescence. It is vital to protect the cervical spine when performing airway maneuvers - Need some statement about positioning and cushing/support should account for possible spine deformity to protect the spinal cord (i.e. severe kyphoscoliosis) - Although some extension of the neck may be essential to enable accurate insertion of a TIVAD and this ought to be considered specifically - consideration should be given to cervical fixation surgery in advance of other operations/procedures | |

| **Statement** | | **Consensus achieved (yes/no) (%)** |
| --- | --- | --- |
| **Pre-operative and intra-operative measures to avoid hypotension should be adopted during all surgical procedures in patients with MPS IVA/VI to maintain spinal cord perfusion and therefore protect spinal cord function** | | Yes (98) |
| **Comments** | | |
| *If you disagree with the statement, please explain why and suggest an amendment below:* | - Avoiding hypotension in all patients including ones with normal anatomy and CNS perfusion may increase the operation time and complications rate of some surgeries (ear and nose) where we need hypo-normal tension to stop excessive bleeding | |
| *Additional comments or suggestions (Optional):* | - Again, preoperative MR scanning of the cervical region will identify those at risk of spinal cord hypoperfusion. In those at risk, consideration should be given to the institution of invasive arterial monitoring before induction. Similarly, to avoid post induction hypotension in-theatre induction should be considered | |

| **Statement** | | **Consensus achieved (yes/no) (%)** |
| --- | --- | --- |
| **Intra-operative neurophysiological monitoring (including somatosensory evoked potentials [SSEP], electromyography [EMG] and motor evoked potentials [MEP]) is strongly recommended during all spinal surgeries and other potentially lengthy or complicated procedures, including those that require manipulation of the head and neck** | | Yes (94) |
| **Comments** | | |
| *If you disagree with the statement, please explain why and suggest an amendment below:* | - Agree with the use for spinal surgery but its use in other non-spinal surgical procedures is not proven and may complicate management - I feel the wording should be 'considered for other lengthy or complicated surgery’, not strongly recommended as there is no evidence supporting its use and the use complicates anesthesia in many circumstances | |
| *Additional comments or suggestions (Optional):* | - I recommend only SSEP and MEP. EMG only for carpal tunnel suspicion - It is doubtful if this helps and how wise it is to stop the procedure if it is halfway through and taking the risks of repeat anesthesia at a later time with no certainty that this won’t happen when attempted again. It is extremely important to explain the risks to the patient prior to undertaking the above surgeries | |

| **Statement** | | **Consensus achieved (yes/no) (%)** |
| --- | --- | --- |
| **For other surgeries and procedures, neurophysiologic monitoring should be considered based on pre-existing risk for spinal cord compression and instability, need for spine manipulation, possibility of hemodynamic changes and blood loss, or extended length of time** | | Yes (94) |
| **Comments** | | |
| *If you disagree with the statement, please explain why and suggest an amendment below:* | - This leaves too much to chance. All patients with MPS should have spinal cord monitoring for anesthetized procedures exceeding one hour - Should not become a mandatory requirement for all surgery | |
| *Additional comments or suggestions (Optional):* | - If possible | |

| **Statement** | | **Consensus achieved (yes/no) (%)** |
| --- | --- | --- |
| **Intrathecal and epidural techniques should be used with extreme caution in MPS VI, due to the anatomical challenges of very short stature, as well as spinal abnormalities causing insertion problems and unpredictability of spread of local anesthesia. However, these techniques may be considered to avoid general anesthesia in a high-risk situation or during pregnancy** | | Yes (88) |
| **Comments** | | |
| *If you disagree with the statement, please explain why and suggest an amendment below:* |  | |
| *Additional comments or suggestions (Optional):* | - Any such technique should be planned with care and the availability of MR imaging of the spinal column to assess the potential for complications during such procedures. Insertion techniques using U/S or x-ray imaging are recommended - Would add to both MPS IVA and VI--use of epidural anesthesia in the post-operative period for pain control should be avoided or used with extreme caution to avoid spine injury during movement in the ICU or ward setting | |

| **Statement** | | **Consensus achieved (yes/no) (%)** |
| --- | --- | --- |
| **Intrathecal and epidural techniques are high-risk in patients with MPS IVA and should be avoided wherever possible** | | Yes (83) |
| **Comments** | | |
| *If you disagree with the statement, please explain why and suggest an amendment below:* | - Again, although spinal stenosis is frequently encountered the degree may vary. Assessment of any spinal stenosis will assist decision making - This is a blanket statement - there are some less severe MPS IV patients who might well be very suited to spinal anesthesia - not least because it enables the patient to remain conscious | |
| *Additional comments or suggestions (Optional):* | - Same comments as above | |

| **Statement** | | **Consensus achieved (yes/no) (%)** |
| --- | --- | --- |
| **Hip reconstruction can be considered in pediatric MPS IVA patients who exhibit hip pain, reduced walking and endurance related to hip disease, as well as abnormal radiographic findings** | | Yes (86) |
| **Comments** | | |
| *If you disagree with the statement, please explain why and suggest an amendment below:* | - It's hard to isolate the symptoms related to hip from other complex problems of spine/lower limbs disease and decision making should include other factors besides radiographic findings - I agree but it is important that any reconstructive surgery is also likely to lead to improved quality of life and not just improvement of X-ray appearances. Also, it is imperative that any reconstructive surgery does not make any subsequent hip replacement procedure more difficult or higher risk. See also answer to question below, regarding hip replacement in pediatric patients - My limited experience with this has not been positive and I would be concerned that such a patient would not be able to comply with the enhanced physical therapy needed postoperatively to keep mobile - Can be considered but should not necessarily be done. There is relatively little evidence that surgery is of benefit | |
| *Additional comments or suggestions (Optional):* | - Considered yes, but we do not know the best procedure or even if our interventions around the hip are successful, so we should proceed cautiously | |

Limb Surgeries in MPS IVA

| **Statement** | | **Consensus achieved (yes/no) (%)** |
| --- | --- | --- |
| **Hip replacement can be considered in adult MPS IVA patients who exhibit hip pain, reduced walking and endurance related to hip disease, as well as abnormal radiographic findings** | | Yes (100) |
| **Comments** | | |
| *If you disagree with the statement, please explain why and suggest an amendment below:* |  | |
| *Additional comments or suggestions (Optional):* | - Growth modulation is recommended in all MPS IVA patients who have evidence of genu valgum and should be performed as early as possible during the period of growth - Hip replacement should also be considered in pediatric patients where reconstructive surgery is unlikely to lead to a significant improvement in quality of life and pain relief/functional gain. I have seen some stunning functional and QOL improvements in such pediatric MPS patients after THA - Weight issues should also be discussed | |

| **Statement** | | **Consensus achieved (yes/no) (%)** |
| --- | --- | --- |
| **Growth modulation is recommended in all MPS IVA patients who have evidence of genu valgum and should be performed as early as possible during the period of growth** | | Yes (77) |
| **Comments** | | |
| *If you disagree with the statement, please explain why and suggest an amendment below:* | - Moderate genu valgum is well tolerable in children with MPS IVA. Recurrence of deformity is highly expected after growth modulation. Indications for surgery should be done considering remaining growth and clinical data as well as general condition and other musculoskeletal programs - The text relates to surgery, yet surgery is not mentioned in the statement. When I first read this, I thought it referred to growth hormone and that has a very uncertain place in management. It should read: Growth modulation surgery is recommended in all MPS IVA patients who have evidence of genu valgum and should be performed as early as possible during the period of growth - Should be considered. Not effective in older children - I agree with correction of genu valgum and disagree that growth modulation is the correct choice in all patients - I won't recommend the surgery as early as possible, because there is a high rate of recurrence in a young patient. If there is no clinical need to correct the deformity (gait problems, pain) I would recommend the epiphyseodesis around the age of 8 | |
| *Additional comments or suggestions (Optional):* | - "As early as possible" may imply when they child is very young. Your supporting text states "prior to age 10". You may want to rephrase this statement to something like "early in the period of growth to allow adequate time for correction" - However, should proceed with caution as the family need to be aware that the likelihood for having to repeat the surgery is high - "As early as possible" means already at the age of 4-6. It may be necessary to leave the devices in place for 2-3 years or more to assess a good correction. The procedure can be repeated in case of recurrence of the deformity | |

| **Statement** | | **Consensus achieved (yes/no) (%)** |
| --- | --- | --- |
| **Hip replacement can be considered in adult MPS VI patients who exhibit hip pain, reduced walking and endurance related to hip disease, as well as abnormal radiographic findings** | | Yes (100) |
| **Comments** | | |
| *If you disagree with the statement, please explain why and suggest an amendment below:* |  | |
| *Additional comments or suggestions (Optional):* | - See comments above on type IV | |

Limb Surgeries in MPS VI

| **Statement** | | **Consensus achieved (yes/no) (%)** |
| --- | --- | --- |
| **Hip reconstruction is not routinely indicated but may be considered in pediatric MPS VI patients who exhibit hip pain, reduced walking and endurance related to hip disease, as well as abnormal radiographic findings** | | Yes (92) |
| **Comments** | | |
| *If you disagree with the statement, please explain why and suggest an amendment below:* | - Would consider to earlier, like in MPS1 patients, but realize there is not enough evident at the moment - See comments above on type IV | |
| *Additional comments or suggestions (Optional):* | - We should always consider if our interventions will help and if they are indicated in the patient as a whole, considering other medical issues and overall fragility | |

| **Statement** | | **Consensus achieved (yes/no) (%)** |
| --- | --- | --- |
| **Growth modulation is recommended in MPS VI patients who have signs of genu valgum and should be performed as early as possible during the period of growth** | | Yes (87) |
| **Comments** | | |
| *If you disagree with the statement, please explain why and suggest an amendment below:* | - See above for MPS IV - Should be considered - See MPS IVA | |
| *Additional comments or suggestions (Optional):* | - See above comments | |

| **Statement** | | **Consensus achieved (yes/no) (%)** |
| --- | --- | --- |
| **Decompression of the spinal cord is recommended in MPS IVA patients who have evidence of spinal cord compression based on clinical and radiographic findings** | | Yes (97) |
| **Comments** | | |
| *If you disagree with the statement, please explain why and suggest an amendment below:* | - It depends on the case and other systemic manifestation | |
| *Additional comments or suggestions (Optional):* | - The statement should take into account other life-threatening morbidities. In particular airway and cardiac. Can the statement start with “If safe to do so” - Unless compression is so advanced at diagnosis that expected benefit likely to be minimal - Or preferably even on radiographic criteria alone - Decompression alone in a pediatric patient with MPS IVA may lead to further instability. Decompression may need to be combined with fusion | |

Spinal surgeries in MPS IVA

| **Statement** | | **Consensus achieved (yes/no) (%)** |
| --- | --- | --- |
| **Spinal stabilization of the craniocervical junction with either cervical fusion or occipital-cervical fusion is recommended in MPS IVA patients who have evidence of instability** | | Yes (97) |
| **Comments** | | |
| *If you disagree with the statement, please explain why and suggest an amendment below:* | - I´m not sure that this procedure is indicated without clinical symptoms - Depending on degree of instability. May be reasonable to watch & wait if minor upper C-spine instability, particularly if minimal compression | |
| *Additional comments or suggestions (Optional):* | - If safe to do so - Especially if there is worsening spinal cord compression - "Instability" with clinical and/or NMR and/or neurophysiological symptoms - Age and severity of instability should be considered | |

| **Statement** | | **Consensus achieved (yes/no) (%)** |
| --- | --- | --- |
| **Correction of thoracolumbar kyphoscoliosis is recommended in MPS IVA patients who present with progressive radiographic deformity, intractable pain and neurological deterioration** | | Yes (100) |
| **Comments** | | |
| *If you disagree with the statement, please explain why and suggest an amendment below:* |  | |
| *Additional comments or suggestions (Optional):* | - Thinking about this more - in the guidelines, balance of risk could preface the discussion with this statement - Unusual scenario - Not and progressive radiographic deformity, intractable pain or neurological deterioration | |

Spinal surgeries in MPS VI

| **Statement** | | **Consensus achieved (yes/no) (%)** |
| --- | --- | --- |
| **Spinal stabilization of the craniocervical junction with either cervical fusion or occipital-cervical fusion is recommended in MPS VI patients who have evidence of instability** | | Yes (100) |
| **Comments** | | |
| *If you disagree with the statement, please explain why and suggest an amendment below:* | - As per type 4 above | |
| *Additional comments or suggestions (Optional):* | - However, it is important to note that these patients’ risk of luxation is lower than that of MPS IV Patients - Age and severity of instability should be considered | |

| **Statement** | | **Consensus achieved (yes/no) (%)** |
| --- | --- | --- |
| **Decompression of the spinal cord is recommended in MPS VI patients who have evidence of spinal cord compression based on clinical and radiographic findings** | | Yes (97) |
| **Comments** | | |
| *If you disagree with the statement, please explain why and suggest an amendment below:* | - I’m not sure | |
| *Additional comments or suggestions (Optional):* |  | |

| **Statement** | | **Consensus achieved (yes/no) (%)** |
| --- | --- | --- |
| **Correction of thoracolumbar kyphoscoliosis is recommended in MPS VI patients who present with progressive radiographic changes, intractable pain and clinical deterioration as defined by gait, lung function and changes in the degree of kyphosis** | | Yes (97) |
| **Comments** | | |
| *If you disagree with the statement, please explain why and suggest an amendment below:* | - Unusual scenario. Interesting that you have stated a clinical deterioration/changing gait as indications for surgery in this group but neurological deterioration for the MPS IVA patients. Should we not use the same terminology? | |
| *Additional comments or suggestions (Optional):* |  | |

Ophthalmic surgery in MPS IVA

| **Statement** | | **Consensus achieved (yes/no) (%)** |
| --- | --- | --- |
| **While significant corneal clouding is rare in MPS IVA patients, corneal transplantation can be considered for patients with significant visual loss attributed to corneal opacification** | | Yes (95) |
| **Comments** | | |
| *If you disagree with the statement, please explain why and suggest an amendment below:* | - It is rare, and I would think first in a cataract as the main cause of visual loss | |
| *Additional comments or suggestions (Optional):* |  | |

Ophthalmic surgery in MPS VI

| **Statement** | | **Consensus achieved (yes/no) (%)** |
| --- | --- | --- |
| **Corneal transplantation can be considered for MPS VI patients with significant visual loss attributed to corneal opacification** | | Yes (100) |
| **Comments** | | |
| *If you disagree with the statement, please explain why and suggest an amendment below:* |  | |
| *Additional comments or suggestions (Optional):* |  | |

Carpal tunnel decompression in MPS VI

| **Statement** | | **Consensus achieved (yes/no) (%)** |
| --- | --- | --- |
| **Decompression of the median nerve and tensosynovectomy of all flexor tendons in the carpal tunnel is recommended in MPS VI patients who display flexion contractures and distal interphalangeal (DIP) joints and/or proximal interphalangeal (PIP) joints (clawing), as well as clinical symptoms of hand pain and/or numbness in the thumb to middle finger, or in patients with positive nerve conduction studies** | | Yes (89) |
| **Comments** | | |
| *If you disagree with the statement, please explain why and suggest an amendment below:* | - Before decompression of the carpal tunnel, nerve conduction studies have to be performed - Positive NCS are always needed unless symptoms are 'classical' for median nerve compression. I rarely do tenosynovectomy - I agree that decompression of median nerve should be done with an abnormal nerve conduction, but cannot answer issue related to tensosynovectomy | |
| *Additional comments or suggestions (Optional):* | - But assessment of the ulnar nerve is often overlooked and may be equally important - There needs to be standardization of nerve conduction studies in this population. Tensosynovectomy needed in some but not all flexor tendons in the carpal tunnel | |

| **Statement** | | **Consensus achieved (yes/no) (%)** |
| --- | --- | --- |
| **A1 and A3 pulley release is recommended in MPS VI patients who display obvious trigger finger** | | Yes (94) |
| **Comments** | | |
| *If you disagree with the statement, please explain why and suggest an amendment below:* | - I have had little recurrence following A1 release alone assuming that on table there is free tendon excursion | |
| *Additional comments or suggestions (Optional):* |  | |

Cardio-thoracic surgery in MPS IVA

| **Statement** | | **Consensus achieved (yes/no) (%)** |
| --- | --- | --- |
| **Cardiac (aortic, mitral) valve replacement should be considered in patients with MPS IVA who display symptomatic and severe valve stenosis or regurgitation** | | Yes (95) |
| **Comments** | | |
| *If you disagree with the statement, please explain why and suggest an amendment below:* | - It depends on the case and other clinical manifestation | |
| *Additional comments or suggestions (Optional):* | - Did not see very often n MPS IVA - only once - Patients are high surgical risks (especially given risks of spinal hypoperfusion/airway concerns/spinal cord instability). If possible, intra-vascular non-invasive valvar replacement should be strongly considered if possible - Qualified, as this may apply to very severe patients some of whom may be more appropriately palliated due to the advanced nature of multisystem disease. However, in the situation that the cardiac valve disease is the main issue and other areas are OK then this would be appropriate - There are many factors that play into the decision making and each individual patient is different. The mitral and aortic valves require detailed assessment but also the aortic root and ascending aorta (which can become aneurysmal) as well as the coronary arties. In principle any severe valvar lesion should be considered for surgery, the decision to progress for surgery or re-operation as is sometimes necessary depends on the severity of the co-morbidities. These include respiratory, ENT, neuro/spine, renal, liver), muscle strength, cardiac catheter (coronary artery disease with stenosis, pulmonary artery pressures), myocardial function (both systolic and diastolic due to the diastolic heart failure), arrhythmias, the estimated life-expectancy at the stage when the patients become symptomatic from cardiac disease in the setting of the MPS subtype, how debilitating the symptoms are and the patients' wishes. This should always be discussed formally by the MDT after meeting a cardiac surgeon and assessing the risks and patient individually. Subsequently a detailed multidisciplinary management plan delineating all affected organ specialties ought to be drawn up, i.e. need for MLTB/Tracheostomy peri-op, joint pediatric/adult cardiac anesthetist peri-op management, pre-op assessment of neck and spine by neuro/spinal surgeon for stability, detailed high-resolution CT lungs and heart, neuro-monitoring intra-operatively, surgical strategy to deal with small mitral annulus and small aortic root and outflow tract etc. - Decision/timing case by case | |

Feedback 29: Cardio-thoracic surgery in MPS VI

| **Statement** | | **Consensus achieved (yes/no) (%)** |
| --- | --- | --- |
| **Cardiac (aortic, mitral) valve replacement should be considered in patients with MPS VI who display symptomatic and severe valve stenosis or regurgitation** | | Yes (100) |
| **Comments** | | |
| *If you disagree with the statement, please explain why and suggest an amendment below:* |  | |
| *Additional comments or suggestions (Optional):* | - Cardiac (aortic, mitral) valve replacement should be considered in patients with MPS VI who display symptomatic and severe valve stenosis or regurgitation - Patients are high surgical risks (especially given risks of spinal hypo perfusion / airway concerns / spinal cord instability). If possible, intra-vascular non-invasive valvar replacement should be strongly considered if possible - See above, no different - Decision/Timing case by case | |

| **Statement** | | **Consensus achieved (yes/no) (%)** |
| --- | --- | --- |
| **Left ventricular apical aneurysms occur rarely in patients with MPS VI but should be resected whenever possible** | | Yes (85) |
| **Comments** | | |
| *If you disagree with the statement, please explain why and suggest an amendment below:* | - The tissue quality in general is poor and therefore less is more. The critical issue in these patients focusses on the valvar pathology, the aneurysm disease of the ventricle is secondary and if true aneurysm could in theory in very selected patients be treated with plication. The main principle would be decreasing the intracavitary pressure unless a large dyskinetic aneurysm occurs in which case a concomitant procedure after excluding coronary stenosis should be performed - There is no evidence regarding that assumption | |
| *Additional comments or suggestions (Optional):* | - The risks and benefits of surgery need to be considered and to do if can’t be managed conservative | |

| **Statement** | | **Consensus achieved (yes/no) (%)** |
| --- | --- | --- |
| **Tracheostomy is recommended in MPS VI patients that exhibit severe upper airway obstruction, which cannot be treated by an alternative approach, or in patients with severe sleep apnea that is not treatable by CPAP or tonsillectomy and/or adenoidectomy** | | Yes (95) |
| **Comments** | | |
| *If you disagree with the statement, please explain why and suggest an amendment below:* |  | |
| *Additional comments or suggestions (Optional):* | - As a last option, yes - I don't know if it should be recommended - or it should be "offered". Whether or not to go down that route can be a very individual choice - In my experience with this patient cohort they are easier and safer managed peri-and post-operative with a tracheostomy, and even though there is a risk of therefore ending up with a permanent tracheostomy in our experience that never happened, and they were successfully de-cannulated after a few weeks. This is often a critical airway, which in a patient with potential prolonged intubation/airway swelling/compromised respiratory function etc. seems to be a much safer option than not managing the airway with a tracheostomy | |

| **Statement** | | **Consensus achieved (yes/no) (%)** |
| --- | --- | --- |
| **CPAP therapy is recommended for MPS IVA patients who display the presence of OSA which persists after tonsillectomy and/or adenoidectomy** | | Yes (97) |
| **Comments** | | |
| *If you disagree with the statement, please explain why and suggest an amendment below:* | - MPS IVA shows respiratory problems due to tracheal stenosis. CPAP is effective for upper airway stenosis, but tracheal stenosis | |
| *Additional comments or suggestions (Optional):* | - This should be monitored and evaluated with polysomnography. Also, the lower airway should be evaluated at least once prior to initiation of CPAP in order to rule out other c surgical treatable obstructions of the upper airway (as could be Laryngoplasty, epiglottopexy, and reduction of the tongue base) - MPS IVA patients are more prone to present alveolar hypoventilation, different from other MPS types where OSA is more prevalent. Alveolar hypoventilation should always be rule out and non-invasive ventilation is the recommended treatment | |

Respiratory interventions and sleep disorders in MPS IVA

| **Statement** | | **Consensus achieved (yes/no) (%)** |
| --- | --- | --- |
| **NIPPV therapy is recommended for MPS IVA patients who display nocturnal hypoventilation and are unresponsive to CPAP, or display daytime hypoventilation with increased PaCO2 and/or serum HCO3 levels** | | Yes (91) |
| **Comments** | |  |
| *If you disagree with the statement, please explain why and suggest an amendment below:* |  | |
| *Additional comments or suggestions (Optional):* | - NIPPV needs to be spelled out | |

| **Statement** | | **Consensus achieved (yes/no) (%)** |
| --- | --- | --- |
| **Oxygen supplementation during sleep is recommended for MPS IVA patients who exhibit sleep apnea with nocturnal hypoxemia, and who do not tolerate CPAP or NIPPV masks** | | Yes (77) |
| **Comments** | | |
| *If you disagree with the statement, please explain why and suggest an amendment below:* | - Not if they are hypercapneic - Oxygen is minimally effective for OSA or OHS. Alternative therapies that are effective for OSA exist and should be tried. Oxygen therapy masks the desaturation of OSA but may make it worse and usually does not improve patient symptoms - In theory it can be offered but I am not sure if it improves the saturation - There is a high risk of developing hypercapnia. Tracheostomy might be considered in these cases - In these cases, is mandatory an objective evaluation of CO2 once hypoventilation is the more probable cause of nocturnal hypoxemia and supplementary oxygen may made the situation worse. High flow nasal cannula may, eventually, be an option once nocturnal hypoventilation workup is negative - Would advise overnight oximetry with early am blood gas measurement or overnight TCCO2 (TOSCA) recording to monitor impact of supplemental oxygen on CO2 if unable to tolerate NIV - There is the risk or worsening hypercapnia which if unable to use NIV may need to be counselled | |
| *Additional comments or suggestions (Optional):* | - Oxygen supplementation in this circumstance (patient fails CPAP or NIPPV), should be monitored in a sleep lab prior to recommending unsupervised use at home. In the sleep lab, all physiological parameters are measured both with supplemental O2 and without - This should be monitored and evaluated with polysomnography. The risk for hypercapnia hypoventilation needs to be monitored | |

| **Statement** | | **Consensus achieved (yes/no) (%)** |
| --- | --- | --- |
| **MPS IVA patients should be monitored for development of hypercapnia after starting oxygen therapy with measurement of PaCO2 and/or serum HCO3** | | Yes (97) |
| **Comments** | | |
| *If you disagree with the statement, please explain why and suggest an amendment below:* | - Oxygen is not an option for MPS IVA patients! PaCO2 and serum HCO3 are not good markers to evaluate hypoventilation (any type of) since it takes a long time to be shown in blood gases or serum. Transcutaneous CO2 is the gold standard although the use of O2 in MPS is at least controversial | |
| *Additional comments or suggestions (Optional):* | - As above. Oxygen can then be considered to be administered via NIV or unable to tolerate NIV patient/carers counselled of consequences and impact on CO2 | |

| **Statement** | | **Consensus achieved (yes/no) (%)** |
| --- | --- | --- |
| **NIPPV therapy is recommended for MPS VI who display nocturnal hypoventilation and are unresponsive to CPAP, or display daytime hypoventilation with increased PaCO2 and/or serum HCO3 levels** | | Yes (94) |
| **Comments** | | |
| *If you disagree with the statement, please explain why and suggest an amendment below:* | - Comment same as before - Some comments made in the prior section - I don't think I understand this question, I thought that non-invasive positive pressure ventilation (NIPPV) includes continuous positive airway pressure (CPAP) and bilevel positive airway pressure (BiPAP), in this question do you mean BiPAP? | |
| *Additional comments or suggestions (Optional):* | - Consider in presence of nocturnal hypoventilation irrespective of lack of response to CPAP or not | |

Respiratory Interventions and Sleep Disorders in MPS VI

| **Statement** | | **Consensus achieved (yes/no) (%)** |
| --- | --- | --- |
| **CPAP is recommended therapy for MPS VI patients who display the presence of OSA which persists after tonsillectomy and/or adenoidectomy** | | Yes (100) |
| **Comments** | | |
| *If you disagree with the statement, please explain why and suggest an amendment below:* |  | |
| *Additional comments or suggestions (Optional):* | - This should be monitored and evaluated with polysomnography. Also, the lower airway should be evaluated at least once prior to initiation of CPAP in order to rule out other c surgical treatable obstructions of the upper airway (as could be Laryngoplasty, epiglottopexy, reduction of the tongue base) | |

| **Statement** | | **Consensus achieved (yes/no) (%)** |
| --- | --- | --- |
| **Oxygen supplementation during sleep is recommended for MPS VI patients that display sleep apnea with nocturnal hypoxemia, and who do not tolerate CPAP or NIPPV masks** | | Yes (83) |
| **Comments** | | |
| *If you disagree with the statement, please explain why and suggest an amendment below:* | - Same as before - There is a high risk of developing hypercapnia - Same comments make before - Consider oximetry and transcutaneous CO2 monitoring overnight and early am blood gas if using oxygen during sleep to identify any worsening hypercapnia - AS for MPS IV patients, advise overnight monitoring of CO2 to assess impact of uncontrolled oxygen on ventilation if unable to use NIV | |
| *Additional comments or suggestions (Optional):* | - Oxygen supplementation in this circumstance (patient fails CPAP or NIPPV), should be monitored in a sleep lab prior to recommending unsupervised use at home. In the sleep lab, all physiological parameters are measured both with supplemental O2 and without - Same as before | |

| **Statement** | | **Consensus achieved (yes/no) (%)** |
| --- | --- | --- |
| **MPS VI patients should be monitored for development of hypercapnia after starting oxygen therapy with measurement of PaCO2 and/or serum HCO3** | | Yes (97) |
| **Comments** | | |
| *If you disagree with the statement, please explain why and suggest an amendment below:* | - Same comments from the prior section | |
| *Additional comments or suggestions (Optional):* | - All very obvious, we haven’t seen these issues after transplantation | |

ENT Surgery in MPS IVA

| **Statement** | | **Consensus achieved (yes/no) (%)** |
| --- | --- | --- |
| **Tonsillectomy and/or adenoidectomy is recommended for MPS IVA patients who display recurrent otitis media, or snoring and/or obstructive sleep apnea (OSA) as early as possible following diagnosis without waiting for disease progression** | | Yes (94) |
| **Comments** | | |
| *If you disagree with the statement, please explain why and suggest an amendment below:* | - For severe OSA patients or patients with pulmonary hypertension CPAP treatment should be performed before surgery - I typically do not recommend T&A for only history of recurrent OM | |
| *Additional comments or suggestions (Optional):* | - While T&A is strongly recommended, the entire medical hx of the patient should be considered and weighed into the decision of the timing of scheduling a T&A - Recurrent otitis media is not an indication for T&A surgery and should be excluded from the sentence | |

| **Statement** | | **Consensus achieved (yes/no) (%)** |
| --- | --- | --- |
| **Insertion of ventilation tubes is recommended for MPS IVA patients with otitis media with effusion and/or recurrent otitis media to maintain hearing and/or prevent recurrent acute otitis media** | | Yes (100) |
| **Comments** | | |
| *If you disagree with the statement, please explain why and suggest an amendment below:* |  | |
| *Additional comments or suggestions (Optional):* | - Ventilation tubes have been recommended and insertion is frequent, however I doubt about their usefulness: they do not seem to improve symptoms and displace very often. Children with ventilation tubes are not opposed to go to the swimming pool | |

| **Statement** | | **Consensus achieved (yes/no) (%)** |
| --- | --- | --- |
| **Uvulopalatopharyngoplasty and/or mandibular advancement surgeries should be considered in MPS IVA patients who display the presence of OSA which persists after tonsillectomy and/or adenoidectomy** | | No (55) |
| **Comments** | | |
| *If you disagree with the statement, please explain why and suggest an amendment below:* | - Uvulopalatopharyngoplasty and/or mandibular advancement surgeries are major surgeries that may or may not benefit MPS IVA patients. There are less invasive ways of improving their OSA symptoms - Not recommended - Need some publications, even case reports - Risks of surgery balanced with limited benefit of uvuloplasty make this intervention less than desirable for MPS patients - I do not have any experience of UPPP for MPS IV. But respiratory distress in MPS IV is due to tracheal stenosis, so that UPPP is not - Due to the changed anatomic features in these patients the accessibility to the soft palate will be likely very limited. No surgery without visibility of the surgical field! - Possibly useful, there is no sufficient experience. This kind of intervention probably carries high risk of severe adverse events like death - Very controversial. Results of UVVP even in normal adult patients are no good. The site of obstruction is multilevel, especially in this population. Mandibular advancement is not enough since the have macroglossia in the majority of the cases - A step too far | |
| *Additional comments or suggestions (Optional):* | - Should qualify "up to age 11." After this age, UPPP and MAD surgery have much less success and CPAP should be considered first line after T&A - Could be instead of should be will be more suitable here | |

| **Statement** | | **Consensus achieved (yes/no) (%)** |
| --- | --- | --- |
| **Partial tongue reduction could be considered in MPS IVA patients who display the presence of OSA which persists after tonsillectomy and/or adenoidectomy** | | No (42) |
| **Comments** | | |
| *If you disagree with the statement, please explain why and suggest an amendment below:* | - Tongue reduction is a dangerous procedure, massive swelling of the tongue after operation is a not uncommon complication - Partial tongue reduction surgeries are major surgeries that may or may not benefit MPS IVA patients. There are less invasive ways of improving their OSA symptoms - Procedure is minimally effective and not first line after T&A - Not recommended for MPS patients - Need publications, even case reports - I do not think it is effective for MPS. Pharyngeal mucosa as well as tongue also thickened - Tongue reduction eliminates the anterior and lateral part of the tongue, but the effect of the tongue base is very limited. Any surgery would need to address this area - Possibly useful, there is no sufficient experience. This kind of intervention probably carries high risk of severe adverse events like death - Not an option in these population - In 17 years of managing these patients I have never had to have this type of discussion with families - The enlarged tongue is not typically the cause of OSA | |
| *Additional comments or suggestions (Optional):* |  | |

| **Statement** | | **Consensus achieved (yes/no) (%)** |
| --- | --- | --- |
| **Tracheostomy is recommended in MPS IVA patients that do not respond to any of the treatment modalities mentioned above** | | Yes (77) |
| **Comments** | | |
| *If you disagree with the statement, please explain why and suggest an amendment below:* | - Should be considered - Well, tracheostomy is always the last option. And stated this way, as a patient who has been given every available treatment by without any improvement, then tracheostomy offers a way of treating severe, life-threatening OSA. We have seen the rate of tracheostomies decrease dramatically since starting ERT in most MPS disease categories. - Always need to try CPAP before tracheostomy - MPS IV showed respiratory distress due to tracheal stenosis around thoracic inlet - Difficult to perform and often difficult to maintain due to abnormal and tortuous trachea often seen in MPS IVA patients especially as they become older | |
| *Additional comments or suggestions (Optional):* | - Again, I would prefer the term "offered" - It is to expect that the tracheostomy is technical difficult, due to the short neck and very limited possibility to retrocline the head (instability of the atlanto-axial joint). The size of the used cannula and the shape (length) will likely need to be adjusted to the patient’s personal features - Any decision to insert tracheostomy should be a multi-disciplinary team discussion with patient and carers to understand the longer-term implication, care needs and potential for tracheostomy ventilation | |

ENT Surgery in MPS VI

| **Statement** | | **Consensus achieved (yes/no) (%)** |
| --- | --- | --- |
| **Tonsillectomy and/or adenoidectomy is recommended in MPS VI patients who display upper airway obstruction, recurrent otitis media, snoring and/or OSA as early as possible following diagnosis, without waiting for disease progression** | | Yes (91) |
| **Comments** | | |
| *If you disagree with the statement, please explain why and suggest an amendment below:* | - While T&A is strongly recommended, the entire medical history of the patient should be considered and weighed into the decision of the timing of scheduling a T&A - Same comments made before - I typically do not recommend T&A for only history of recurrent OM | |
| *Additional comments or suggestions (Optional):* | - Same as MPS IVA, exclude | |

| **Statement** | | **Consensus achieved (yes/no) (%)** |
| --- | --- | --- |
| **Uvulopalatopharyngoplasty and/or mandibular advancement surgeries should be considered in MPS VI patients, who display the presence of OSA which persists after tonsillectomy and/or adenoidectomy** | | No (65) |
| **Comments** | | |
| *If you disagree with the statement, please explain why and suggest an amendment below:* | - Uvulopalatopharyngoplasty and/or mandibular advancement surgeries are major surgeries that may or may not benefit MPS VI patients. There are less invasive ways of improving their OSA symptoms - See MPS IVA - Need to have some published data in MPS. Would not make this very specific recommendations in key recommendations - See explanation for MPS IVA above - No evidence for these procedures. Should be done in controlled and individual case by case need only - Pharyngeal mucosa as well as tongue also thickened, I do not think it is effective for MPS - Possibly useful, there is no sufficient experience. This kind of intervention probably carries high risk of severe adverse events like death - A step too far | |
| *Additional comments or suggestions (Optional):* | - See comments for IVA - Due to the changed anatomic features in these patients the accessibility to the soft palate will be likely very limited. No surgery without visibility of the surgical field! - Same as MPS IVA | |

| **Statement** | | **Consensus achieved (yes/no) (%)** |
| --- | --- | --- |
| **Partial tongue reduction could be considered in MPS VI patients, who display the presence of OSA which persists after tonsillectomy and/or adenoidectomy** | | No (64) |
| **Comments** | | |
| *If you disagree with the statement, please explain why and suggest an amendment below:* | - Tongue reduction is a dangerous procedure, massive swelling of the tongue after operation is a not uncommon complication - Partial tongue reduction surgeries are major surgeries that may or may not benefit MPS VI patients. There are less invasive ways of improving their OSA symptoms - See comments for MPS IVA - Same as above - As above - I do not have any experience of partial tongue reduction - Possibly useful, there is no sufficient experience. This kind of intervention probably carries high risk of severe adverse events like death - Already comment before - Never had to do this and find it abhorrent practice | |
| *Additional comments or suggestions (Optional):* | - Tongue reduction eliminates the anterior and lateral part of the tongue, but the effect of the tongue base is very limited | |

| **Statement** | | **Consensus achieved (yes/no) (%)** |
| --- | --- | --- |
| **Insertion of ventilation tubes is recommended in MPS VI patients with otitis media with effusion and/or recurrent otitis media to maintain hearing and/or prevent recurrent acute otitis media** | |  |
| **Comments** | | |
| *If you disagree with the statement, please explain why and suggest an amendment below:* | - I never saw improvements of symptoms after ventilation tubes insertion. Also, they frequently displace and the patients with ventilation tubes are not allowed to go to the swimming pool | |
| *Additional comments or suggestions (Optional):* |  | |
