# Supplementary material for: Recommendations for the management of MPS VI: systematic evidence- and consensus-based guidance
Source: Orphanet J Rare Dis. 2019 May 29;14:118. doi: 10.1186/s13023-019-1080-y (PMC6541999; doi:10.1186/s13023-019-1080-y)
Supplement: Supplementary file 5 — Modified-Delphi voting Round 2. Full results of the second round of the modified-Delphi voting, which was used to demonstrate consensus of the guidance statements. (DOCX 41 kb) [file 13023_2019_1080_MOESM5_ESM.docx]

## Additional File 5: modified-Delphi voting Round 2 results

## **Results**

A total of 71 survey submissions were received from 53 individual hospitals/institutions across 18 countries, this included 19 responses from members of the Steering Committee. Following the analysis of the results, two respondents did not meet the minimum experience threshold and their submissions were excluded, therefore giving a total of 69 respondents to round 2 of the modified-Delphi survey.

Table 1. Summary of respondents to Round 2 of modified-Delphi voting by specialism, following exclusion of submissions that did not meet the minimum experience threshold

| **List of specialisms** | **Number of respondents** |
| --- | --- |
| Bone marrow transplant expert/Hematopoietic stem cell transplant expert | 3 |
| Ear-nose-throat specialist | 5 |
| Geneticist | 12 |
| Hand surgeon | 3 |
| Neurosurgeon | 3 |
| Orthopedic surgeon | 7 |
| Pediatrician | 15 |
| Pulmonologist/Respiratory physician | 4 |
| Radiologist | 2 |
| Other: Adult inherited metabolic disorders | 1 |
| Other: Adult metabolic medicine | 1 |
| Other: Anesthetist | 3 |
| Other: Critical care physician and pulmonologist specializing in home ventilation (CPAP/NIV) | 1 |
| Other: Genetic Counsellor/Support officer | 1 |
| Other: Hematologist | 1 |
| Other: Internist | 1 |
| Other: Pain specialist | 1 |
| Other: Pediatric neurologist | 1 |
| Other: Pediatric neuropsychologist | 1 |
| Other: Pediatric rehabilitation specialist | 1 |
| Other: Plus pharmacologist and toxicologist | 1 |
| Other: Surgeon working with rare diseases | 1 |
| **Total** | **69** |

Table 2. Summary of respondents to Round 2 of modified-Delphi voting by country, following exclusion of submissions that did not meet the minimum experience threshold

| **List of countries** | **Number of respondents** |
| --- | --- |
| Argentina | 1 |
| Australia | 4 |
| Austria | 1 |
| Brazil | 5 |
| Canada | 8 |
| Colombia | 2 |
| Czech Republic | 1 |
| Germany | 4 |
| Italy | 3 |
| Japan | 1 |
| Netherlands | 3 |
| Portugal | 1 |
| Russia | 1 |
| South Africa | 1 |
| Spain | 1 |
| Turkey | 3 |
| UK | 12 |
| USA | 17 |
| **Total** | **69** |

Table 3. modified-Delphi voting results for general principles for the management of MPS IVA/VI

| **Statement** | **Number of respondents** | **Percentage consensus** | **Consensus achieved (yes/no)** |
| --- | --- | --- | --- |
| General principles for the management of MPS IVA/VI | | | |
| Management of pain should be a fundamental part of the care of patients with MPS IVA/MPS VI, with the aim of improving QoL and maintaining mobility. Refer to general guidelines for pain management | 46 | 100% | Yes |

Table 4. modified-Delphi voting results for routine monitoring and assessments in MPS IVA/VI

| **Statement** | **Number of respondents** | **Percentage consensus** | **Consensus achieved (yes/no)** |
| --- | --- | --- | --- |
| Radiology | | | |
| Standing or sitting plain radiography of the cervical and thoracolumbar spine to examine for spinal deformities is recommended in patients with MPS IVA/VI at diagnosis and every 2–3 years thereafter, or sooner if clinically indicated | 41 | 85% | Yes |
| Magnetic resonance imaging (MRI) of the brain is recommended at diagnosis in patients with MPS IVA/VI, and should be repeated as needed in individuals with clinical suspicion of hydrocephalus | 40 | 80% | Yes |
| Flexion/extension computerized tomography (CT) of the craniocervical junction may be considered in patients with MPS IVA/VI if MRI is not available or if sedation is not possible | 39 | 92% | Yes |
| [Neurology](file:///\\10.0.0.5\shared\Lucid%20Partners%20UK\Client\BioMarin\2018\2.%20MPS%20Consensus%20programme\13.%20Delphi\Delphi%20Results\Round%202\MPS%20Consensus%20Program_Results%20from%20Round%202%20modified-Delphi%20Voting_v1.0_120418.docx#FB3) | | | |
| Standard MRI of the cervical spine should be performed to assess for presence of spinal cord compression in patients with MPS IVA/VI. In the absence of significant spinal cord compression, proceed with flexion/extension MRI to confirm the presence of worsening spinal cord compression with motion | 41 | 78% | Yes |
| Upper limb function | | | |
| Standardized clinical examination, assessment of active and passive range of movement and nerve conduction studies (NCS) are recommended to assess hand and upper limb function in patients with MPS VI | 44 | 89% | Yes |
| Ear-nose-throat (ENT) | | | |
| ENT examination in patients with MPS IVA/VI should include visualization of the upper respiratory tract to determine diagnosis, management and assist in pre-operative planning. Endoscopic examinations should be recorded and kept, to monitor disease progression | 39 | 92% | Yes |
| Fiberoptic examination in patients with MPS IVA/VI should be performed at diagnosis and at least annually thereafter, or as clinically indicated. For those individuals who require general anesthesia, ENT examination should be performed during pre-operative evaluation conducted for other surgical procedures | 36 | 83% | Yes |
| Upper airway CT focused on airway anatomy, preferably with reconstruction, may be useful to identify the area of the abnormality and possible cause of obstruction in patients with MPS IVA/VI with suspected obstruction or malacia | 37 | 92% | Yes |

Table 5. modified-Delphi voting results for disease-modifying interventions

| **Statement** | **Number of respondents** | **Percentage consensus** | **Consensus achieved (yes/no)** |
| --- | --- | --- | --- |
| Enzyme replacement therapy (galsulfase) in MPS VI | | | |
| Initiation of long-term ERT with galsulfase at a dose of 1 mg/kg/week with intravenous infusion is recommended in patients with MPS VI as soon as possible after a confirmed diagnosis | 35 | 89% | Yes |
| [Hematopoietic stem cell transplantation in MPS IVA/VI](file:///\\10.0.0.5\shared\Lucid%20Partners%20UK\Client\BioMarin\2018\2.%20MPS%20Consensus%20programme\13.%20Delphi\Delphi%20Results\Round%202\MPS%20Consensus%20Program_Results%20from%20Round%202%20modified-Delphi%20Voting_v1.0_120418.docx#FB7) | | | |
| Due to the lack of evidence, HSCT cannot be recommended for patients with MPS IVA and at this time is considered an investigational procedure | 35 | 91% | Yes |
| With consideration of the associated risk of morbidity and mortality associated with this procedure, HSCT may be an option for patients with MPS VI who have a matched related donor (or unrelated donor), or cord blood graft | 28 | 86% | Yes |
| Due to the risk of mortality, it is critical that HSCT is only performed in an institution with a multidisciplinary team experienced in the care of patients with MPS VI | 35 | 91% | Yes |

## Respondent feedback for each Key Action Statement

*General principles for management*

| **Statement** | | **Consensus achieved (yes/no) (%)** |
| --- | --- | --- |
| **Management of pain should be a fundamental part of the care of patients with MPS IVA/MPS VI, with the aim of improving QoL and maintaining mobility. Refer to general guidelines for pain management** | | Yes (100) |
| **Comments** | | |
| *If you disagree with the statement, please explain why and suggest an amendment* |  | |
| *Additional comments or suggestions* | - Pain is one of the most important areas that commits QoL. All efforts should be done to minimize - I recognize that joint pain is a frequent symptom faced by MPS IVA/VI patients; however, aside from nonsteroidal inflammatory medications and (very rarely) opioids, there are very few pharmacologic interventions that can truly relieve the pain. I have never referred my patients to a pain management specialist - perhaps as I fill out this consensus I am learning new things as well - Adequate pain management is a fundamental part of any disease including MPS IVA and VI to improve the quality of life - General pain clinics are often not a good fit for these patients and they often benefit from more specialized care in relation to chronic pain - Drugs dosage must be personalized in MPS patients because they may have a slower than normal metabolism. It would also be beneficial that we will be able to eliminate the cause of pain - This may require collaboration with a pain team as required | |

*Recommended routine monitoring and assessments in MPS IVA/VI*

| **Statement** | | **Consensus achieved (yes/no) (%)** |
| --- | --- | --- |
| **Standing or sitting plain radiography of the cervical and thoracolumbar spine to examine for spinal deformities is recommended in patients with MPS IVA/VI at diagnosis and every 2–3 years thereafter, or sooner if clinically indicated** | | Yes (85) |
| **Comments** | | |
| *If you disagree with the statement, please explain why and suggest an amendment below:* | - It is unsafe to expose adults to this level of radiation if there is no clinical indication - Once baseline established, clinical follow up will be more important than radiographic surveillance - I have some level of agreement with this statement, but in a young child, I think 2-3 is too long and 1-2 years is more appropriate - While C/T/L radiography is fast, it does not allow visualization of the spinal cord. So, we have moved to MR imaging of the spine to query for kyphoscoliosis as well as spinal cord stenosis/compression - There has to be good clinical indication to expose patients to radiation. As it is a lifelong condition any routine X-ray imaging will lead to significant life time exposure to radiation - The indications for radiography is more related to the clinical aspect than to a rigid program of periodical examinations | |
| *Additional comments or suggestions (Optional):* | - Strongly agree at the time of diagnosis, but later on, X-ray control is clinically dependent, MRI may be better approach? - Cervical spine impact is less than thoracolumbar. I prefer MRI - Also at discretion of spinal/orthopaedic teams | |

| **Statement** | | **Consensus achieved (yes/no) (%)** |
| --- | --- | --- |
| **MRI of the brain is recommended at diagnosis in patients with MPS IVA/VI, and should be repeated as needed in individuals with clinical suspicion of hydrocephalus** | | Yes (80) |
| **Comments** | | |
| *If you disagree with the statement, please explain why and suggest an amendment below:* | - If merely looking for hydrocephalus, CT is more readily available, is faster and is less likely to require sedation - Hydrocephalus is less common than in MPS I and II? - Only if clinical suspicion - We would like to see/monitor the evolution of possible changes (after intervention) - MRI of the brain has little to no benefit in MPS IV - I don't think brain MRI scan absolutely needs to be performed at diagnosis unless there is a clinical indication. Young patients require sedation or anaesthesia for brain MRI - Hydrocephalus is very rare in MPS IVA especially at diagnosis which is often in childhood. Brain MR lesions are unusual. MR of the spine is certainly indicated, and it may be appropriate to extend one of the spinal protocols to include a scout view of the whole head but routine MR of the brain in MPS IVA I don't think can be justified in all areas - In MPS VI I think this would be appropriate - MRI scan of a patient at diagnosis might not be needed unless there are findings suggestive of brain involvement such as headache, increased head circumference, seizures etc. | |
| *Additional comments or suggestions (Optional):* | - Hydrocephalus or other symptoms that rise suspicion on a new central neurological condition - At the same time, we request flexion/extension views on MRI - these sequences are also doable for a patient under anesthesia as well and have supplanted plain films and CT scans (see above/below) | |

*Radiology in MPS IVA/VI*

| **Statement** | | **Consensus achieved (yes/no) (%)** |
| --- | --- | --- |
| **Flexion/extension computerized tomography (CT) of the craniocervical junction may be considered in patients with MPS IVA/VI if MRI is not available or if sedation is not possible** | | Yes (92) |
| **Comments** | | |
| *If you disagree with the statement, please explain why and suggest an amendment below:* | - Initial screening could be with conventional radiography - Given modern day concerns of radiation exposure from CT scans for the young, as well as increased resolution/visualization for MR imaging, we do not perform CT scanning unless the patient cannot undergo MRI (sedation vs. implanted devices not compatible) - Yes, if it can be explained by the information it will offer to help with the management of the patient. Again, a base line CT is reasonable but routine CT i.e. every 3 yearly in place of MRI will not be sensible | |
| *Additional comments or suggestions (Optional):* | - The statement in isolation is not correct but when in context with other statements is acceptable. If this was my first round of Delphi I would not have known the context, I would have disagreed and suggested it should be altered to include wording "may be considered in patients with spinal stenosis or clinical symptoms suggesting cord involvement” - I would do in all patients at least once to detect instability - Computerized tomography can help to evaluate C1C2 instability, sometime better than MRI | |

Neurology in MPS IVA/VI

| **Statement** | | **Consensus achieved (yes/no) (%)** |
| --- | --- | --- |
| **Standard MRI of the cervical spine should be performed to assess for presence of spinal cord compression in patients with MPS IVA/VI. In the absence of significant spinal cord compression, proceed with flexion/extension MRI to confirm the presence of worsening spinal cord compression with motion** | | Yes (78) |
| **Comments** | | |
| *If you disagree with the statement, please explain why and suggest an amendment below:* | - F/E MRI is limited accessibility. Plain supine MRI combined with radiographic F/E should be another option - I prefer CT scan in flexion and extension – it’s faster and avoids any compression of the spinal cord for longer time in case of instability with cord compression - The criteria for assessment of the degree of compression are unclear for flexion/extension MRI. Additional studies are needed to avoid misinterpretation - I would only proceed to flex/ex MRI if there were concerns of instability on plain films or other clinical concerns. Not routinely warranted - This should certainly be considered as a part of pre-anesthetic assessment when going for surgery under general anesthetic and if there are clinical symptoms and signs. I am not sure about it being done as a routine monitoring. A decision to operate will involve how the patient is affected and won’t be based purely on radiology - Not sure why you restricted the procedure to the "absence of significant cord compression." This is the particular patient you most need to assess instability and impact on the cord anatomy - If clinically indicated - These facilities may not always be available. MRI and Fx/Ex plain radiographs may be sufficient to analyze C1/C2 subluxation - I have not experience about MRI in sedation, so I fear spinal cord injury during this procedure | |
| *Additional comments or suggestions (Optional):* | - Is this in asymptomatic patients? - Only if the patient is not under sedation and with active movements of the patient - As guided by neurosurgery too | |

*Upper limb function in MPS IVA/VI*

| **Statement** | | **Consensus achieved (yes/no) (%)** |
| --- | --- | --- |
| **Standardized clinical examination, assessment of active and passive range of movement and nerve conduction studies (NCS) are recommended to assess hand and upper limb function in patients with MPS VI** | | Yes (89) |
| **Comments** | | |
| *If you disagree with the statement, please explain why and suggest an amendment below:* | - Not sure about routine NCS - Agree to clinical examination, then NCS if clinically indicated - I agree with examination, passive/active ROM. However, we do not routinely order NCS. There may be utility for NCS if the patient is demonstrating signs significant for carpal tunnel syndrome or myelopathy, but even then, MR imaging may be more useful to identify myelopathy - Agree with clinical examination. NCS when clinical indication - Nerve conduction studies to be performed if clinically indicated | |
| *Additional comments or suggestions (Optional):* | - Since there are not very strong clinical signs of CTS I would do NCV | |

Ear-nose-throat (ENT) surgeries in MPS IVA/VI

| **Statement** | | **Consensus achieved (yes/no) (%)** |
| --- | --- | --- |
| **ENT examination in patients with MPS IVA/VI should include visualization of the upper respiratory tract to determine diagnosis, management and assist in pre-operative planning. Endoscopic examinations should be recorded and kept, to monitor disease progression** | | Yes (92) |
| **Comments** | | |
| *If you disagree with the statement, please explain why and suggest an amendment below:* | - I neither agree nor disagree - ENT evaluation not so helpful in clarifying diagnosis - Invasive procedures to be performed when clinically indicated | |
| *Additional comments or suggestions (Optional):* | - It should be mentioned that the examination in the awake patient in a sitting position is likely to give an "optimistic" estimation of the upper airway compared to the situation of the patient asleep (during anaesthesia) especially in prone position. During the later situation the upper airway collapses due to the muscle relaxation and the resulting posterior placement of the tongue - We do laryngoscopy in all patients - Video/photographic documentation, if at all possible during endoscopic assessment is invaluable both for follow up assessments as well as for anaesthetic pore-op planning - Again, as guided by ENT, anaesthesia and respiratory | |

| **Statement** | | **Consensus achieved (yes/no) (%)** |
| --- | --- | --- |
| **Fiberoptic examination in patients with MPS IVA/VI should be performed at diagnosis and at least annually thereafter, or as clinically indicated. For those individuals who require general anesthesia, ENT examination should be performed during pre-operative evaluation conducted for other surgical procedures** | | Yes (83) |
| **Comments** | | |
| *If you disagree with the statement, please explain why and suggest an amendment below:* | - Assuming the diagnosis is made at a young age, the airway is likely to be normal and remain that way for some time. Baseline evaluation at diagnosis with re-evaluation around age 10, then yearly or biannually thereafter would be reasonable - The staff should consider the high risk before performing the fiberoptic examination in some patients. So, the indication should be individualized - I would not subject patients to routine fiberoptic examination. Is very reasonable when planning on anesthetic intervention - Do not perform routine fiberoptic evaluations in ENT examination. This exam is performed if we have evidence of significant airway compromise, i.e. abnormal sleep study not easily corrected by cPAP. Our anesthesiologist performs exam during fiberoptic intubation - These invasive investigations should be performed when clinically indicated - Individuals with near normal upper airways may not need a fiberoptic exam every year if not undergoing a procedure and no or little indication of upper airway obstruction | |
| *Additional comments or suggestions (Optional):* | - It should be mentioned that the examination in the awake patient in a sitting position is likely to give an "optimistic" estimation of the upper airway compared to the situation of the patient asleep (during anaesthesia) especially in prone position. During the later situation the upper airway collapses due to the muscle relaxation and the resulting posterior placement of the tongue - If it’s a non-classical patient with no airway narrowing I would not repeat annually but every 2 years and pre-operative - Pre-operative flexible nasendosocpy allows direct visualization of the larynx to assess ease of intubation. Additionally, if ENT are present at pre-operative planning, it allows for discussion of further options to secure the airway in the event of an emergency including a tracheostomy - Again, as guided by ENT, anaesthesia & respiratory | |

| **Statement** | | **Consensus achieved (yes/no) (%)** |
| --- | --- | --- |
| **Upper airway CT focused on airway anatomy, preferably with reconstruction, may be useful to identify the area of the abnormality and possible cause of obstruction in patients with MPS IVA/VI who have suspected obstruction or malacia** | | Yes (92) |
| **Comments** | | |
| *If you disagree with the statement, please explain why and suggest an amendment below:* | - Leave out "preferably with reconstruction". CT is useful to evaluate patient with significant airway disease, abnormal PFT, sleep study, etc. - The movement study such as fiberoptic upper airway evaluation is far more informative and should be performed when it is available - I don't radiate children unless it will change management | |
| *Additional comments or suggestions (Optional):* | - Airway tortuosity and laryngotracheobronchomalacia are exceedingly common in especially adolescent/older MPS IVA/VI patients and the CT reconstruction is very useful - I do at least once before surgery - Even if the larynx is easy to visualize on nasendoscopy, distal tortuosity cannot be assessed. 3D CT scans facilitate peri-operative planning | |

***Disease-modifying Interventions***

Enzyme replacement therapy (galsulfase) in MPS VI

| **Statement** | | **Consensus achieved (yes/no) (%)** |
| --- | --- | --- |
| **Initiation of long-term ERT with galsulfase at a dose of 1 mg/kg/week with intravenous infusion is recommended in patients with MPS VI as soon as possible after a confirmed diagnosis** | | Yes (89) |
| **Comments** | | |
| *If you disagree with the statement, please explain why and suggest an amendment below:* | - I disagree with this "broad" statement. The degree of disease impairment, burden and the expected impact of ERT should be discussed and weighed before considering treatment initiation - I would suggest alteration to "Initiation of long term ERT with Galsulfase should be considered in patients with MPS VI as soon as...." In some patients with very slowly progressive disease a period of observation may be necessary to adequately assess the risk/benefit ratio of enzyme replacement therapy. In patients with more rapidly progressive or classical disease consideration may be given to him and stem cell transplantation as an alternative therapy possibly with enzyme replacement therapy being given initially to improve the patient's health and reduce risks of the HSCT procedure - HSCT should also be considered - In general, yes, but my hesitancy would be with attenuated phenotypes. In very attenuated patients there may be an argument for watching and waiting as the rate of progression may be very slow and quality of life may in fact be reduced by commencing ERT. However, in my experience even apparently attenuated patients often have a significant hidden disease burden which justifies the use of ERT | |
| *Additional comments or suggestions (Optional):* | - Strongly agree only in small children to demonstrate ERT efficacy - Generally agree but unless very severely affected and not in the parent’s best interests - Following assessment of clinical picture and discussion with patient with regards to their view on long-term treatment - Yes, agree in principle with and assumption that patient is affected by the condition. Asymptomatic patients or with no clinical features should be monitored - This may also depend on patient/family wishes and severity of disease, i.e. if too severe and unlikely to respond- perhaps a limited trial is warranted | |

Hematopoietic stem cell transplantation in MPS IVA/VI

| **Statement** | | **Consensus achieved (yes/no) (%)** |
| --- | --- | --- |
| **Due to the lack of evidence, HSCT cannot be recommended for patients with MPS IVA, and at this time is considered an investigational procedure** | | Yes (91) |
| **Comments** | |  |
| *If you disagree with the statement, please explain why and suggest an amendment below:* | - It might be considered where ERT not possible (reactions, or cost) and only when data are subsequently gathered about efficacy - Some case reports are more optimistic, HSCT may be discussed with parents of small children? - I would not say that it is investigational, it just doesn't help the bone disease, and there is no cognitive disease to treat | |
| *Additional comments or suggestions (Optional):* | - I still don't like the "non-randomized survey" language here, as the data regarding transplant is not survey based, but is a report of clinical experience. I also don't like the statement "In this situation only, the associated risk of HSCT is considered justified by some physicians". This assumes that there are no benefits with transplant that cannot be achieved with ERT, and I don't know that is true | |

| **Statement** | | **Consensus achieved (yes/no) (%)** |
| --- | --- | --- |
| **With consideration of the risk of morbidity and mortality associated with this procedure, HSCT may be an option for patients with MPS VI who have a matched related donor, or with an unrelated donor or cord blood graft** | | Yes (86) |
| **Comments** | |  |
| *If you disagree with the statement, please explain why and suggest an amendment below:* | - If no ERT is available, yes - I believe HSCT may be potentially life threatening therefore I would not recommend - Long-term impact of HSCT in MPS VI is not clear and unlikely to be different than long-term ERT impact but with higher risk - Need consideration of the "significant" risk. Check with BMT specialists, but not sure "with unrelated donor or” is correct. Expect that it should be unrelated donor cord blood graft. My impression is that we still need some level of match even for an unrelated cord blood graft | |
| *Additional comments or suggestions (Optional):* | - Match related donor should not be a carrier - Some improvement was observed after HSCT with good donor - Still investigational with the hope that it could be more effective than ERT | |

| **Statement** | | **Consensus achieved (yes/no) (%)** |
| --- | --- | --- |
| **Due to the risk of mortality, it is critical that HSCT is only performed in an institution with a multidisciplinary team experienced in the care of patients with MPS VI** | | Yes (91) |
| **Comments** | |  |
| *If you disagree with the statement, please explain why and suggest an amendment below:* | - I think that "only" should be replaced by "preferably" - I believe HSCT may be potentially life threatening therefore I would not recommend - Since I do not agree that HSCT should strongly be considered as a treatment option for MPS VI it would be nonsensical for me to answer this. In principle I agree with the isolated statement but not the implications of the statement | |
| *Additional comments or suggestions (Optional):* | - Agree and especially where ERT is difficult due to cost or reactions, and where the donor is well matched - The issue here is, how does one define "a multidisciplinary team experienced in the care of patients with MPS VI"? - If the option is HSCT, so it must be performed in a specialized center with experience in MPS | |
